# Supplementary material for: Combined health risks of cigarette smoking and low levels of physical activity: a prospective cohort study in England with 12-year follow-up
Source: BMJ Open. 2019 Nov 27;9(11):e032852. doi: 10.1136/bmjopen-2019-032852 (PMC6887020; doi:10.1136/bmjopen-2019-032852)
Supplement: Supplementary data [file bmjopen-2019-032852supp001.pdf]

Supplementary material

Method: calculation of Bayes factors

We used a conservative approach with alternative hypotheses represented by a half-normal distribution and expected effect sizes to RR=3 based on previous research that demonstrated a large multiplicative effect of smoking and BMI on circulatory disease mortality (1), and RR=2 and RR=1.5 to test for medium and small synergistic effects. BFs ≥ 3 can be interpreted as evidence for the alternative hypothesis (and against the null), BFs $\leq 1/3$ as evidence for the null hypothesis, and BFs between 1/3 and 3 suggest the data are insensitive to distinguish the alternative hypothesis from the null (2).

Results: sensitivity analyses

Imputing outcomes at Wave 8 for participants who dropped out and did not report the presence of the outcome prior to dropout produced a very similar pattern of results, but the larger sample size meant 95% CIs were narrower and *p* values were smaller (Supplementary Table 2, Supplementary Figure 1). Multiplicative interactions between smoking and physical activity remained non-significant for all outcomes, with the exception of chronic lung disease which became statistically significant (p=0.044).

Restricting the sample to those with complete data at baseline (Wave 2) and final follow-up (Wave 8) also produced a similar pattern of results, although RRs were attenuated for the diagnosed diseases (CHD, stroke, cancer, chronic lung disease) (Supplementary Table 3, Supplementary Figure 2). The only notable change was that the RR for CHD in low active current smokers fell below that of low active former smokers, but this was likely caused by the reduced sample size leading to imprecise estimates for this group (*n*=121, 12 cases of incident CHD). Interactions between smoking and physical activity remained non-significant for all outcomes.

Excluding current smokers with lower levels of dependence (i.e. those who smoked <15 cigarettes per day, *n*=369) did not notably alter the results (Supplementary Table 4, Supplementary Figure 3), although as was observed when missing data were imputed, the interaction between smoking and physical activity for risk of developing chronic lung disease became statistically significant (p=0.033).

Jackson SE, *et al. BMJ Open* 2019; 9:e032852. doi: 10.1136/bmjopen-2019-032852

References

1. Freedman DM, Sigurdson AJ, Rajaraman P, Doody MM, Linet MS, Ron E. The Mortality Risk of Smoking and Obesity Combined. Am J Prev Med. 2006 Nov;31(5):355–62.

2. Dienes Z. Using Bayes to get the most out of non-significant results. Front Psychol [Internet]. 2014 Jul 29 [cited 2018 Jul 4];5. Available from: https://www.ncbi.nlm.nih.gov/pmc/articles/PMC4114196/

Jackson SE, *et al. BMJ Open* 2019; 9:e032852. doi: 10.1136/bmjopen-2019-032852
