# Supplementary material for: Combined health risks of cigarette smoking and low levels of physical activity: a prospective cohort study in England with 12-year follow-up
Source: BMJ Open. 2019 Nov 27;9(11):e032852. doi: 10.1136/bmjopen-2019-032852 (PMC6887020; doi:10.1136/bmjopen-2019-032852)
Supplement: Supplementary data [file bmjopen-2019-032852supp002.pdf]

Supplementary tables

Supplementary Table 1. Baseline characteristics of participants included in the analysed sample compared with those who were excluded

	Excluded (*n*=3007)	Included (*n*=6425)	*p*
Age in years, mean (SD)	65.60 (13.28)	65.88 (9.34)	0.229
Sex, % (*n*)			
Men	40.7 (1223)	45.2 (2902)	<0.001
Women	59.3 (1784)	54.8 (3523)	-
Ethnicity, % (*n*)			
White	95.0 (2847)	98.8 (6345)	<0.001
Non-white	5.0 (149)	1.2 (80)	-
Wealth quintile, % (*n*)			
1 (poorest)	28.8 (643)	14.6 (940)	<0.001
2	24.0 (536)	18.5 (1188)	-
3	18.0 (403)	20.8 (1338)	-
4	15.3 (341)	22.3 (1432)	-
5 (richest)	14.0 (313)	23.8 (1527)	-
Alcohol intake, % (*n*)			
Never/rarely	23.6 (406)	18.9 (1213)	<0.001
Regularly	42.4 (728)	45.3 (2909)	-
Frequently	34.0 (585)	35.8 (2303)	-
BMI, mean (SD)	17.72 (14.48)	27.91 (4.87)	<0.001
Fair/poor self-rated health[1], % (*n*)	35.1 (1008)	24.5 (1575)	<0.001
Limiting long-standing illness[1], % (*n*)	40.9 (1230)	32.9 (2111)	<0.001
Coronary heart disease[1], % (*n*)	9.9 (299)	8.6 (553)	0.035
Stroke[1], % (*n*)	5.1 (153)	2.4 (152)	<0.001
Cancer[1], % (*n*)	6.9 (208)	7.7 (496)	0.167
Chronic lung disease[1], % (*n*)	7.4 (223)	7.3 (466)	0.777
Clinically relevant depressive symptoms[1], % (*n*)	20.3 (565)	13.5 (860)	<0.001
Smoking status, % (*n*)			
Never smoker	36.1 (1085)	37.2 (2387)	<0.001
Former smoker	44.6 (1341)	48.9 (3141)	-
Current smoker	19.2 (578)	14.0 (897)	-
Level of physical activity, % (*n*)			
High	53.5 (1536)	65.9 (4233)	<0.001
Low	46.5 (1336)	34.1 (2192)	-

BMI = body mass index; SD = standard deviation. Note: due to missing data, numbers do not sum to the total and percentages do not sum to 100 for the group of excluded participants for some variables.

Supplementary Table 2. Bayes factors for multiplicative interactions between smoking status and physical activity

	Large effects (RR=3)		Medium effects (RR=2)		Small effects (RR=1.5)	
	BF	Interpretation	BF	Interpretation	BF	Interpretation
Fair/poor self-rated health	0.23	Moderate evidence for H0	0.36	Data were insensitive	0.56	Data were insensitive
Limiting long-standing illness	0.28	Moderate evidence for H0	0.43	Data were insensitive	0.65	Data were insensitive
Coronary heart disease	0.48	Data were insensitive	0.68	Data were insensitive	0.88	Data were insensitive
Stroke	0.62	Data were insensitive	0.84	Data were insensitive	1.02	Data were insensitive
Cancer	0.30	Moderate evidence for H0	0.45	Data were insensitive	0.65	Data were insensitive
Chronic lung disease	0.04	Strong evidence for H0	0.06	Strong evidence for H0	0.11	Moderate evidence for H0
Clinically relevant depressive symptoms	0.29	Moderate evidence for H0	0.43	Data were insensitive	0.65	Data were insensitive

BF=Bayes factor, H0=null hypothesis, RR=relative risk.

Supplementary Table 3. Main effects of smoking status and physical activity and the interaction between smoking status and physical activity for risks of incident health problems over 12-year follow-up: imputed outcome data for participants who dropped out before Wave 8

	Smoking status			Physical activity		Interaction[1]	
	Never smoker	Former smoker	Current smoker	High active	Low active	Former smoker x low active	Current smoker x low active
Fair/poor self-rated health							
% (*n*)	48.9 (1168)	58.5 (1839)	69.7 (625)	48.9 (2071)	71.2 (1562)	-	-
RR [95% CI]	1	1.20 [1.09-1.32]	1.42 [1.25-1.63]	1	1.46 [1.33-1.60]	-	-
p		<0.001	<0.001		<0.001		
RR_{adj} [95% CI]	1	1.15 [1.02-1.30]	1.52 [1.27-1.81]	1	1.23 [1.05-1.44]	0.95 [0.78-1.15]	0.97 [0.74-1.27]
p		0.023	<0.001		0.012	0.567	0.810
Limiting long-standing illness							
% (*n*)	62.8 (1499)	69.8 (2192)	72.9 (654)	62.1 (2630)	78.2 (1715)	-	-
RR [95% CI]	1	1.11 [1.01-1.22]	1.16 [1.03-1.31]	1	1.26 [1.16-1.37]	-	-
p		0.025	0.019		<0.001		
RR_{adj} [95% CI]	1	1.08 [0.96-1.21]	1.18 [1.00-1.39]	1	1.11 [0.96-1.28]	0.98 [0.82-1.17]	1.06 [0.82-1.37]
p		0.195	0.053		0.166	0.786	0.666
Coronary heart disease							
% (*n*)	14.1 (335)	20.3 (639)	25.3 (227)	14.0 (594)	27.7 (607)	-	-
RR [95% CI]	1	1.45 [1.20-1.75]	1.79 [1.32-2.44]	1	1.97 [1.71-2.27]	-	-
p		<0.001	0.001		<0.001		
RR_{adj} [95% CI]	1	1.21 [0.96-1.53]	2.05 [1.43-2.94]	1	1.37 [1.02-1.82]	1.04 [0.71-1.54]	1.05 [0.69-1.61]
p		0.098	<0.001		0.034	0.829	0.810
Stroke							
% (*n*)	13.7 (327)	18.4 (579)	23.3 (209)	13.4 (567)	25.0 (547)	-	-
RR [95% CI]	1	1.34 [1.04-1.73]	1.70 [1.30-2.22]	1	1.86 [1.55-2.24]	-	-
p		0.027	0.001		<0.001		
RR_{adj} [95% CI]	1	1.20 [0.89-1.63]	2.03 [1.42-2.92]	1	1.42 [1.03-1.97]	0.87 [0.60-1.25]	1.01 [0.67-1.53]
p		0.216	<0.001		0.035	0.441	0.963

CI = confidence interval; RR = relative risk from bivariate models; RR_{adj} = relative risk adjusted for age, sex, ethnicity, wealth, alcohol intake, body mass index, and physical activity (for smoking status) or smoking status (for physical activity).

[1] Multiplicative interaction between smoking status and physical activity. Results can be interpreted as the difference in RR between high active and low active former/current smokers, relative to the difference in RR between high and low active never smokers. Thus, an interaction term above 1 indicates the disparity between high and low active groups was greater for former/current smokers than never smokers, and an interaction term below 1 indicates the disparity between high and low active groups was smaller for former/current smokers than never smokers.

Note: For each outcome, the sample is restricted to those who did not report the presence of the outcome at baseline. Results therefore indicate the prevalence and relative risk of new-onset health problems over the follow-up period.

Supplementary Table 3. (continued)

	Smoking status			Physical activity		Interaction[1]	
	Never smoker	Former smoker	Current smoker	High active	Low active	Former smoker x low active	Current smoker x low active
Cancer							
% (*n*)	18.0 (430)	21.9 (688)	24.6 (221)	17.7 (749)	26.9 (589)	-	-
RR [95% CI]	1	1.22 [1.06-1.40]	1.36 [1.07-1.73]	1	1.52 [1.21-1.90]	-	-
p		0.006	0.013		0.002		
RR_{adj} [95% CI]	1	1.13 [0.93-1.37]	1.57 [1.15-2.16]	1	1.34 [0.98-1.83]	0.96 [0.69-1.32]	0.95 [0.56-1.61]
p		0.207	0.006		0.065	0.792	0.831
Chronic lung disease							
% (*n*)	6.9 (164)	14.1 (443)	32.5 (292)	9.0 (382)	23.6 (517)	-	-
RR [95% CI]	1	2.05 [1.56-2.72]	4.76 [3.10-7.32]	1	2.61 [1.79-3.81]	-	-
p		<0.001	<0.001		0.001		
RR_{adj} [95% CI]	1	2.14 [1.42-3.24]	6.27 [3.88-10.09]	1	2.66 [1.65-4.27]	0.74 [0.49-1.12]	0.63 [0.40-0.99]
p		0.001	<0.001		<0.001	0.157	0.044
Clinically relevant depressive symptoms							
% (*n*)	52.8 (1259)	58.0 (1823)	62.9 (564)	52.4 (2219)	65.1 (1428)	-	-
RR [95% CI]	1	1.10 [0.98-1.24]	1.19 [1.02-1.39]	1	1.24 [1.13-1.36]	-	-
p		0.111	0.029		<0.001		
RR_{adj} [95% CI]	1	1.10 [0.96-1.26]	1.18 [0.98-1.43]	1	1.07 [0.91-1.27]	1.01 [0.84-1.22]	1.09 [0.82-1.43]
p		0.184	0.087		0.396	0.905	0.556

CI = confidence interval; RR = relative risk from bivariate models; RR_{adj} = relative risk adjusted for age, sex, ethnicity, wealth, alcohol intake, body mass index, and physical activity (for smoking status) or smoking status (for physical activity).

[1] Multiplicative interaction between smoking status and physical activity. Results can be interpreted as the difference in RR between high active and low active former/current smokers, relative to the difference in RR between high and low active never smokers. Thus, an interaction term above 1 indicates the disparity between high and low active groups was greater for former/current smokers than never smokers, and an interaction term below 1 indicates the disparity between high and low active groups was smaller for former/current smokers than never smokers.

Note: For each outcome, the sample is restricted to those who did not report the presence of the outcome at baseline. Results therefore indicate the prevalence and relative risk of new-onset health problems over the follow-up period.

Supplementary Table 4. Main effects of smoking status and physical activity and the interaction between smoking status and physical activity for risks of incident health problems over 12-year follow-up: sample restricted to participants with data at Wave 2 and Wave 8

	Smoking status			Physical activity		Interaction[1]	
	Never smoker	Former smoker	Current smoker	High active	Low active	Former smoker x low active	Current smoker x low active
Fair/poor self-rated health							
% (*n*)	31.5 (351)	39.0 (494)	45.4 (129)	32.9 (668)	47.9 (306)	-	-
RR [95% CI]	1	1.24 [1.06-1.45]	1.44 [1.14-1.84]	1	1.45 [1.24-1.71]	-	-
p		0.008	0.003		<0.001		
RR_{adj} [95% CI]	1	1.16 [0.96-1.41]	1.53 [1.13-2.05]	1	1.19 [0.90-1.56]	1.06 [0.74-1.52]	1.11 [0.65-1.91]
p		0.134	0.005		0.223	0.734	0.693
Limiting long-standing illness							
% (*n*)	47.5 (488)	52.6 (599)	50.0 (135)	48.2 (915)	57.1 (307)	-	-
RR [95% CI]	1	1.11 [0.96-1.28]	1.05 [0.83-1.33]	1	1.18 [1.01-1.39]	-	-
p		0.166	0.662		0.040		
RR_{adj} [95% CI]	1	1.09 [0.92-1.30]	1.08 [0.82-1.43]	1	1.07 [0.82-1.38]	0.98 [0.69-1.39]	1.18 [0.69-2.02]
p		0.321	0.572		0.626	0.926	0.543
Coronary heart disease							
% (*n*)	5.7 (73)	7.9 (114)	8.6 (32)	6.1 (138)	9.6 (81)	-	-
RR [95% CI]	1	1.39 [1.03-1.88]	1.52 [0.99-2.35]	1	1.56 [1.17-2.07]	-	-
p		0.033	0.056		0.002		
RR_{adj} [95% CI]	1	1.20 [0.82-1.76]	1.72 [0.99-2.98]	1	1.12 [0.66-1.88]	1.37 [0.72-2.63]	0.94 [0.38-2.35]
p		0.359	0.053		0.677	0.339	0.893
Stroke							
% (*n*)	5.4 (72)	6.5 (100)	8.1 (32)	5.6 (131)	7.9 (73)	-	-
RR [95% CI]	1	1.21 [0.89-1.65]	1.50 [0.98-2.31]	1	1.43 [1.06-1.92]	-	-
p		0.234	0.065		0.019		
RR_{adj} [95% CI]	1	1.02 [0.69-1.51]	1.42 [0.78-2.57]	1	1.07 [0.63-1.81]	1.15 [0.58-2.27]	1.59 [0.64-3.92]
p		0.918	0.247		0.812	0.682	0.315

CI = confidence interval; RR = relative risk from bivariate models; RR_{adj} = relative risk adjusted for age, sex, ethnicity, wealth, alcohol intake, body mass index, and physical activity (for smoking status) or smoking status (for physical activity).

[1] Multiplicative interaction between smoking status and physical activity. Results can be interpreted as the difference in RR between high active and low active former/current smokers, relative to the difference in RR between high and low active never smokers. Thus, an interaction term above 1 indicates the disparity between high and low active groups was greater for former/current smokers than never smokers, and an interaction term below 1 indicates the disparity between high and low active groups was smaller for former/current smokers than never smokers.

Note: For each outcome, the sample is restricted to those who did not report the presence of the outcome at baseline. Results therefore indicate the prevalence and relative risk of new-onset health problems over the follow-up period.

Supplementary Table 4. (continued)

	Smoking status			Physical activity		Interaction[1]	
	Never smoker	Former smoker	Current smoker	High active	Low active	Former smoker x low active	Current smoker x low active
Cancer							
% (*n*)	9.8 (125)	11.6 (173)	11.5 (44)	10.1 (228)	12.9 (114)	-	-
RR [95% CI]	1	1.19 [0.93-1.51]	1.17 [0.82-1.68]	1	1.28 [1.01-1.62]	-	-
p		0.162	0.388		0.044		
RR_{adj} [95% CI]	1	1.22 [0.91-1.64]	1.20 [0.74-1.94]	1	1.38 [0.93-2.06]	0.82 [0.49-1.39]	1.07 [0.51-2.25]
p		0.192	0.460		0.111	0.467	0.864
Chronic lung disease							
% (*n*)	2.6 (34)	5.0 (75)	14.4 (52)	3.4 (78)	9.4 (83)	-	-
RR [95% CI]	1	1.94 [1.28-2.93]	5.54 [3.54-8.66]	1	2.75 [2.00-3.78]	-	-
p		0.002	<0.001		<0.001		
RR_{adj} [95% CI]	1	2.67 [1.41-5.03]	7.02 [3.49-14.12]	1	4.09 [2.01-8.32]	0.47 [0.20-1.09]	0.45 [0.18-1.13]
p		0.002	<0.001		<0.001	0.077	0.088
Clinically relevant depressive symptoms							
% (*n*)	43.7 (283)	44.7 (334)	48.8 (104)	42.9 (480)	49.3 (241)	-	-
RR [95% CI]	1	1.02 [0.84-1.24]	1.12 [0.85-1.47]	1	1.15 [0.95-1.39]	-	-
p		0.832	0.430		0.147		
RR_{adj} [95% CI]	1	1.06 [0.84-1.33]	0.98 [0.68-1.41]	1	1.01 [0.74-1.38]	1.02 [0.67-1.55]	1.37 [0.77-2.41]
p		0.639	0.917		0.965	0.931	0.284

CI = confidence interval; RR = relative risk from bivariate models; RR_{adj} = relative risk adjusted for age, sex, ethnicity, wealth, alcohol intake, body mass index, and physical activity (for smoking status) or smoking status (for physical activity).

[1] Multiplicative interaction between smoking status and physical activity. Results can be interpreted as the difference in RR between high active and low active former/current smokers, relative to the difference in RR between high and low active never smokers. Thus, an interaction term above 1 indicates the disparity between high and low active groups was greater for former/current smokers than never smokers, and an interaction term below 1 indicates the disparity between high and low active groups was smaller for former/current smokers than never smokers.

Note: For each outcome, the sample is restricted to those who did not report the presence of the outcome at baseline. Results therefore indicate the prevalence and relative risk of new-onset health problems over the follow-up period.

Supplementary Table 5. Main effects of smoking status and physical activity and the interaction between smoking status and physical activity for risks of incident health problems over 12-year follow-up: excluding current smokers with low levels of nicotine dependence (<15 cigarettes/day)

	Smoking status			Physical activity		Interaction[1]	
	Never smoker	Former smoker	Current smoker	High active	Low active	Former smoker x low active	Current smoker x low active
Fair/poor self-rated health							
% (*n*)	40.9 (529)	49.0 (744)	63.8 (134)	42.2 (946)	59.3 (461)	-	-
RR [95% CI]	1	1.20 [1.05-1.37]	1.56 [1.23-1.98]	1	1.41 [1.22-1.61]	-	-
p		0.008	<0.001		<0.001		
RR_{adj} [95% CI]	1	1.14 [0.97-1.35]	1.75 [1.29-2.36]	1	1.19 [0.94-1.49]	0.99 [0.74-1.34]	0.99 [0.58-1.67]
p		0.115	<0.001		0.144	0.953	0.955
Limiting long-standing illness							
% (*n*)	57.1 (720)	62.7 (905)	70.8 (148)	58.2 (1282)	69.2 (491)	-	-
RR [95% CI]	1	1.10 [0.97-1.24]	1.24 [0.99-1.56]	1	1.19 [1.04-1.36]	-	-
p		0.143	0.067		0.012		
RR_{adj} [95% CI]	1	1.07 [0.93-1.24]	1.36 [1.03-1.79]	1	1.07 [0.86-1.32]	0.97 [0.73-1.29]	0.97 [0.58-1.63]
p		0.348	0.032		0.548	0.847	0.908
Coronary heart disease							
% (*n*)	8.8 (117)	11.7 (176)	14.9 (34)	9.0 (200)	14.9 (127)	-	-
RR [95% CI]	1	1.33 [1.04-1.70]	1.70 [1.13-2.55]	1	1.65 [1.30-2.09]	-	-
p		0.023	0.011		<0.001		
RR_{adj} [95% CI]	1	1.13 [0.82-1.55]	1.94 [1.10-3.41]	1	1.20 [0.79-1.83]	1.19 [0.70-2.02]	1.15 [0.49-2.69]
p		0.459	0.021		0.392	0.533	0.750
Stroke							
% (*n*)	8.2 (113)	9.9 (159)	12.6 (30)	7.9 (182)	13.1 (120)	-	-
RR [95% CI]	1	1.22 [0.95-1.56]	1.53 [1.00-2.35]	1	1.65 [1.29-2.10]	-	-
p		0.129	0.049		<0.001		
RR_{adj} [95% CI]	1	1.12 [0.80-1.57]	1.69 [0.87-3.26]	1	1.41 [0.93-2.13]	0.81 [0.47-1.39]	1.59 [0.65-3.89]
p		0.498	0.121		0.109	0.437	0.306

CI = confidence interval; RR = relative risk from bivariate models; RR_{adj} = relative risk adjusted for age, sex, ethnicity, wealth, alcohol intake, body mass index, and physical activity (for smoking status) or smoking status (for physical activity).

[1] Multiplicative interaction between smoking status and physical activity. Results can be interpreted as the difference in RR between high active and low active former/current smokers, relative to the difference in RR between high and low active never smokers. Thus, an interaction term above 1 indicates the disparity between high and low active groups was greater for former/current smokers than never smokers, and an interaction term below 1 indicates the disparity between high and low active groups was smaller for former/current smokers than never smokers.

Note: For each outcome, the sample is restricted to those who did not report the presence of the outcome at baseline. Results therefore indicate the prevalence and relative risk of new-onset health problems over the follow-up period.

Supplementary Table 5. (continued)

	Smoking status			Physical activity		Interaction[1]	
	Never smoker	Former smoker	Current smoker	High active	Low active	Former smoker x low active	Current smoker x low active
Cancer							
% (*n*)	13.4 (178)	15.8 (247)	19.8 (48)	13.8 (309)	18.4 (164)	-	-
RR [95% CI]	1	1.18 [0.96-1.45]	1.48 [1.05-2.10]	1	1.33 [1.08-1.63]	-	-
p		0.112	0.027		0.006		
RR_{adj} [95% CI]	1	1.11 [0.85-1.43]	1.85 [1.18-2.91]	1	1.29 [0.91-1.82]	1.01 [0.65-1.57]	0.81 [0.39-1.67]
p		0.446	0.008		0.149	0.974	0.565
Chronic lung disease							
% (*n*)	3.3 (44)	7.8 (120)	26.3 (61)	5.0 (111)	13.0 (114)	-	-
RR [95% CI]	1	2.34 [1.65-3.34]	7.89 [5.22-11.91]	1	2.60 [1.98-3.41]	-	-
p		<0.001	<0.001		<0.001		
RR_{adj} [95% CI]	1	2.80 [1.64-4.79]	11.40 [6.10-21.31]	1	3.49 [1.87-6.50]	0.57 [0.27-1.17]	0.40 [0.17-0.93]
p		<0.001	<0.001		<0.001	0.123	0.033
Clinically relevant depressive symptoms							
% (*n*)	53.5 (418)	56.4 (535)	63.0 (104)	52.4 (668)	62.5 (389)	-	-
RR [95% CI]	1	1.06 [0.90-1.24]	1.18 [0.90-1.55]	1	1.19 [1.02-1.40]	-	-
p		0.512	0.236		0.028		
RR_{adj} [95% CI]	1	1.10 [0.90-1.34]	1.18 [0.82-1.70]	1	1.09 [0.84-1.41]	0.95 [0.68-1.34]	1.11 [0.64-1.95]
p		0.370	0.375		0.527	0.783	0.705

CI = confidence interval; RR = relative risk from bivariate models; RR_{adj} = relative risk adjusted for age, sex, ethnicity, wealth, alcohol intake, body mass index, and physical activity (for smoking status) or smoking status (for physical activity).

[1] Multiplicative interaction between smoking status and physical activity. Results can be interpreted as the difference in RR between high active and low active former/current smokers, relative to the difference in RR between high and low active never smokers. Thus, an interaction term above 1 indicates the disparity between high and low active groups was greater for former/current smokers than never smokers, and an interaction term below 1 indicates the disparity between high and low active groups was smaller for former/current smokers than never smokers.

Note: For each outcome, the sample is restricted to those who did not report the presence of the outcome at baseline. Results therefore indicate the prevalence and relative risk of new-onset health problems over the follow-up period.
