# Supplementary material for: Combined health risks of cigarette smoking and low levels of physical activity: a prospective cohort study in England with 12-year follow-up
Source: BMJ Open. 2019 Nov 27;9(11):e032852. doi: 10.1136/bmjopen-2019-032852 (PMC6887020; doi:10.1136/bmjopen-2019-032852)
Supplement: Supplementary data [file bmjopen-2019-032852supp003.pdf]

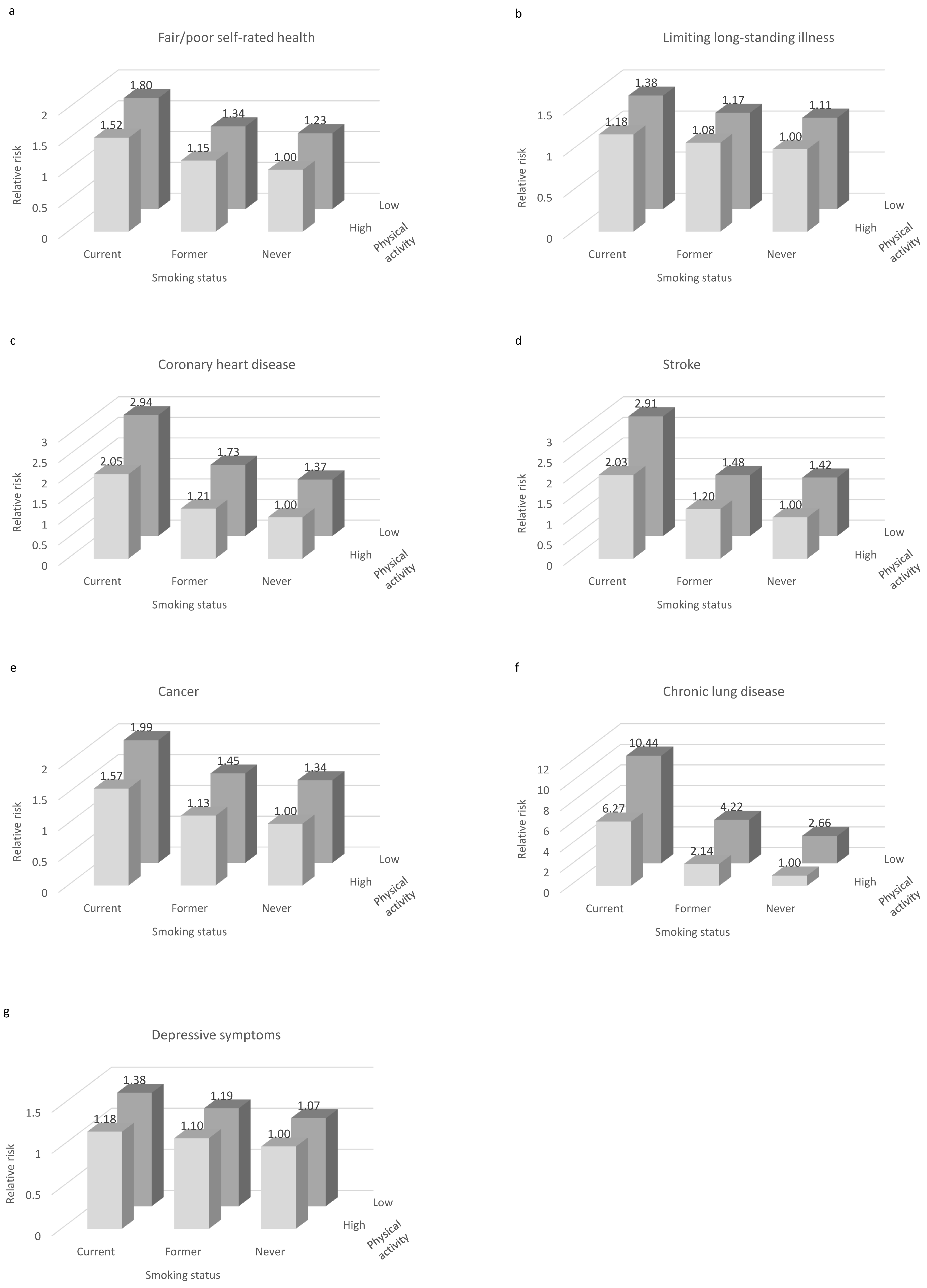
a
Fair/poor self-rated health
1.52 1.80 1.15 1.34 1.00 1.23
Relative risk
0 0.5 1 1.5 2
Current Former Never
Smoking status
Low High
Physical activity
b
Limiting long-standing illness
1.18 1.38 1.08 1.17 1.00 1.11
Relative risk
0 0.5 1 1.5
Current Former Never
Smoking status
Low High
Physical activity
c
Coronary heart disease
2.05 2.94 1.21 1.73 1.00 1.37
Relative risk
0 0.5 1 1.5 2 2.5 3
Current Former Never
Smoking status
Low High
Physical activity
d
Stroke
2.03 2.91 1.20 1.48 1.00 1.42
Relative risk
0 0.5 1 1.5 2 2.5 3
Current Former Never
Smoking status
Low High
Physical activity
e
Cancer
1.57 1.99 1.13 1.45 1.00 1.34
Relative risk
0 0.5 1 1.5 2
Current Former Never
Smoking status
Low High
Physical activity
f
Chronic lung disease
6.27 10.44 2.14 4.22 1.00 2.66
Relative risk
0 2 4 6 8 10 12
Current Former Never
Smoking status
Low High
Physical activity
g
Depressive symptoms
1.18 1.38 1.10 1.19 1.00 1.07
Relative risk
0 0.5 1 1.5
Current Former Never
Smoking status
Low High
Physical activity

Supplementary Figure 1. Relative risks of developing (a) fair/poor self-rated health, (b) limiting long-standing illness, (c) coronary heart disease, (d) stroke, (e) cancer, (f) chronic lung disease, and (g) clinically relevant depressive symptoms over 12-year follow-up by baseline smoking/physical activity status, among older adults free of these conditions at baseline: imputed outcome data for participants who dropped out before Wave 8
