# Supplementary material for: Combined health risks of cigarette smoking and low levels of physical activity: a prospective cohort study in England with 12-year follow-up
Source: BMJ Open. 2019 Nov 27;9(11):e032852. doi: 10.1136/bmjopen-2019-032852 (PMC6887020; doi:10.1136/bmjopen-2019-032852)
Supplement: Supplementary data [file bmjopen-2019-032852supp004.pdf]

a

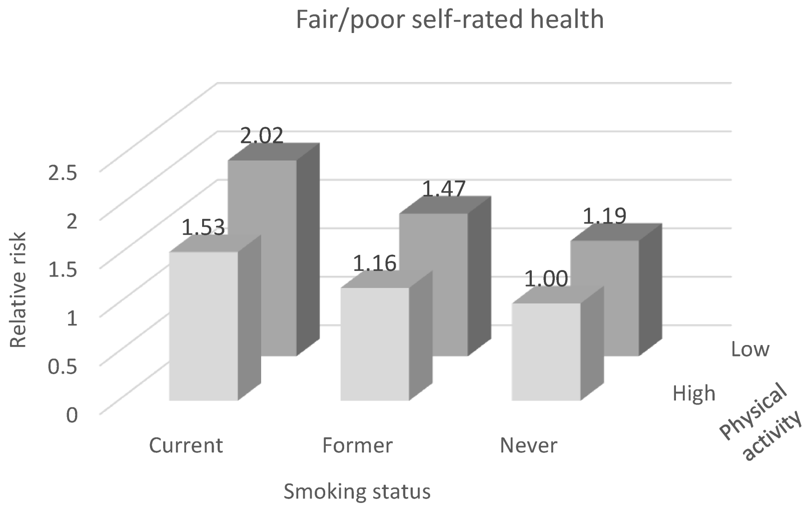
Fair/poor self-rated health
Relative risk
0
0.5
1
1.5
2
2.5
1.53
2.02
1.16
1.47
1.00
1.19
Current
Former
Never
Smoking status
Low
High
Physical activity

b

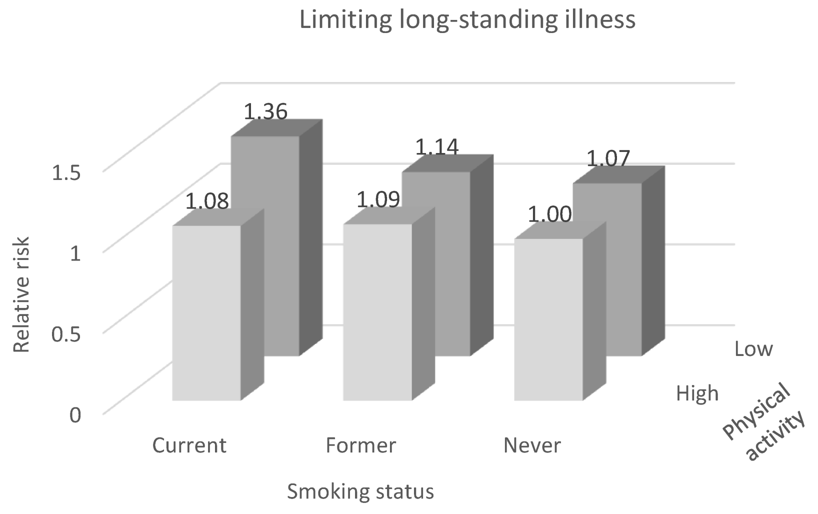
Limiting long-standing illness
Relative risk
0
0.5
1
1.5
1.08
1.36
1.09
1.14
1.00
1.07
Current
Former
Never
Smoking status
Low
High
Physical activity

c

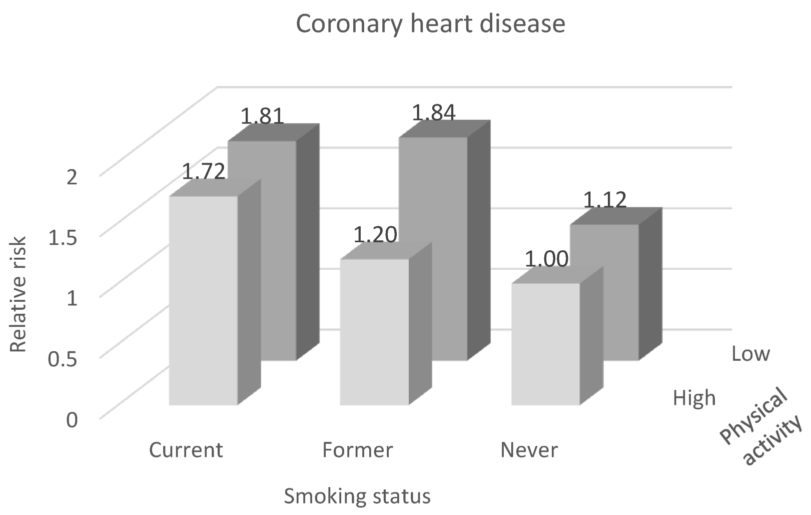
Coronary heart disease
Relative risk
0
0.5
1
1.5
2
1.72
1.81
1.20
1.84
1.00
1.12
Current
Former
Never
Smoking status
Low
High
Physical activity

d

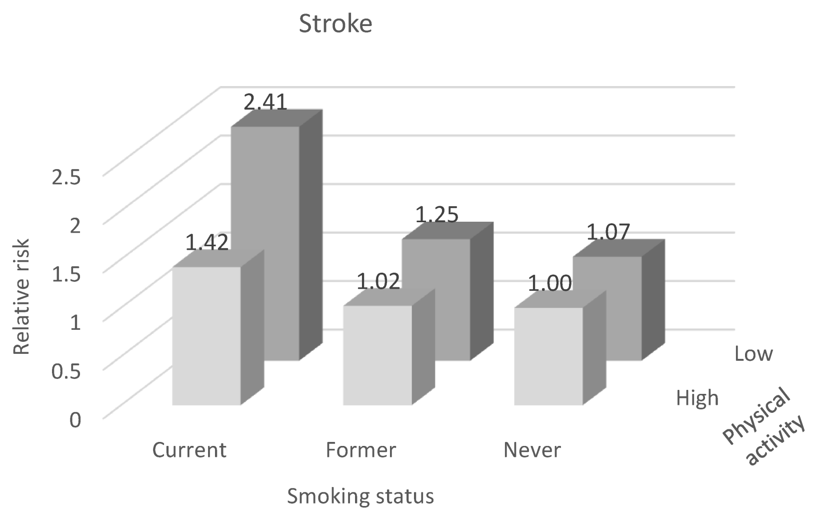
Stroke
Relative risk
0
0.5
1
1.5
2
2.5
1.42
2.41
1.02
1.25
1.00
1.07
Current
Former
Never
Smoking status
Low
High
Physical activity

e

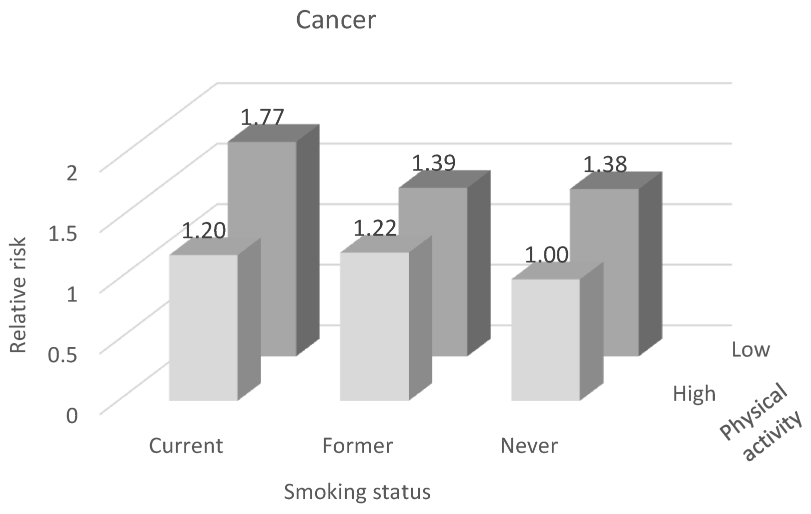
Cancer
Relative risk
0
0.5
1
1.5
2
1.20
1.77
1.22
1.39
1.00
1.38
Current
Former
Never
Smoking status
Low
High
Physical activity

f

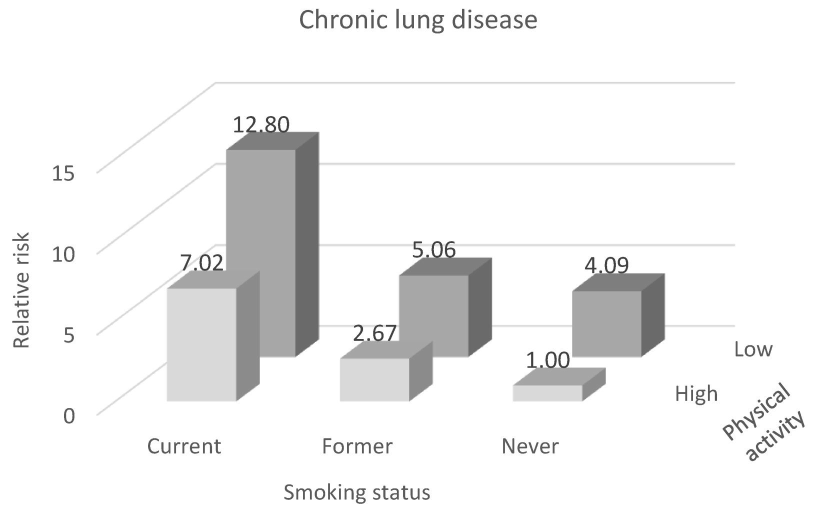
Chronic lung disease
Relative risk
0
5
10
15
7.02
12.80
2.67
5.06
1.00
4.09
Current
Former
Never
Smoking status
Low
High
Physical activity

g

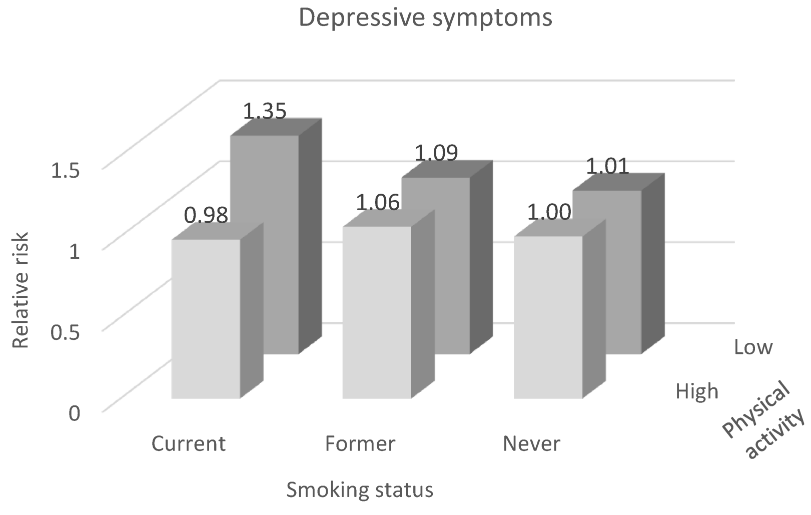
Depressive symptoms
Relative risk
0
0.5
1
1.5
0.98
1.35
1.06
1.09
1.00
1.01
Current
Former
Never
Smoking status
Low
High
Physical activity

Supplementary Figure 2. Relative risks of developing (a) fair/poor self-rated health, (b) limiting long-standing illness, (c) coronary heart disease, (d) stroke, (e) cancer, (f) chronic lung disease, and (g) clinically relevant depressive symptoms over 12-year follow-up by baseline smoking/physical activity status, among older adults free of these conditions at baseline: sample restricted to participants with data at Wave 2 and Wave 8
