# Supplementary material for: Combined health risks of cigarette smoking and low levels of physical activity: a prospective cohort study in England with 12-year follow-up
Source: BMJ Open. 2019 Nov 27;9(11):e032852. doi: 10.1136/bmjopen-2019-032852 (PMC6887020; doi:10.1136/bmjopen-2019-032852)
Supplement: Supplementary data [file bmjopen-2019-032852supp005.pdf]

a

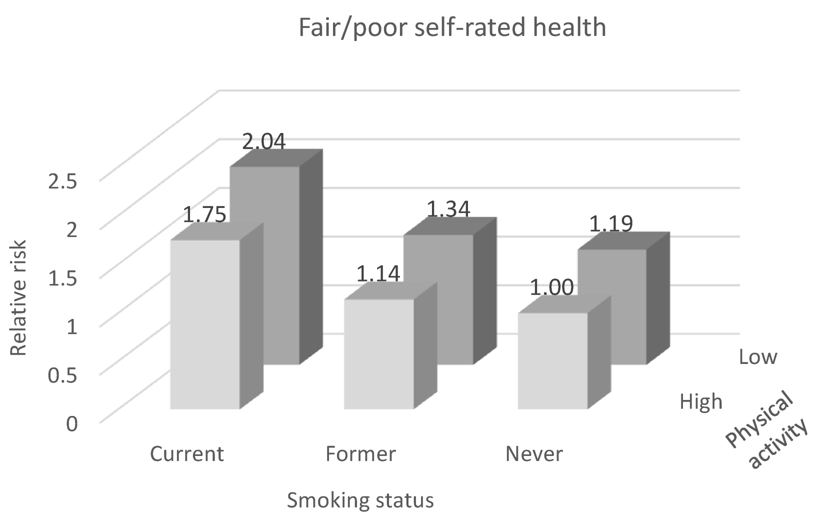
Fair/poor self-rated health
Relative risk
0
0.5
1
1.5
2
2.5
1.75
2.04
1.14
1.34
1.00
1.19
Current
Former
Never
Smoking status
Low
High
Physical activity

b

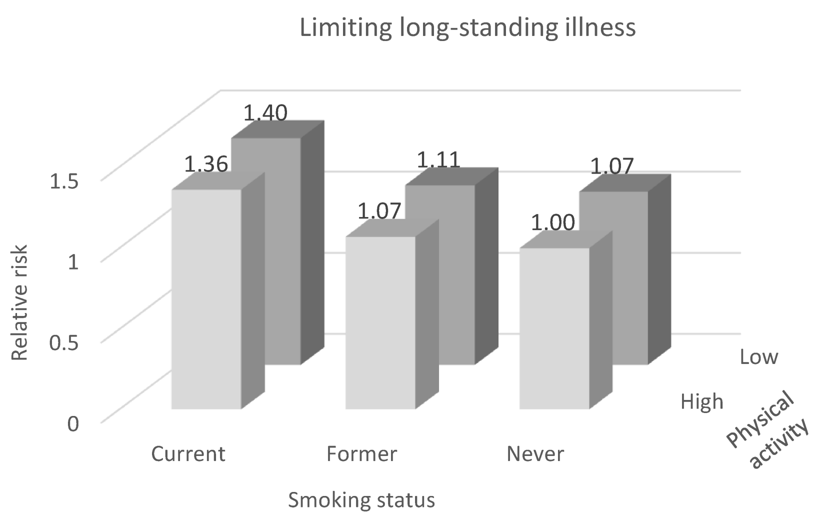
Limiting long-standing illness
Relative risk
0
0.5
1
1.5
1.36
1.40
1.07
1.11
1.00
1.07
Current
Former
Never
Smoking status
Low
High
Physical activity

c

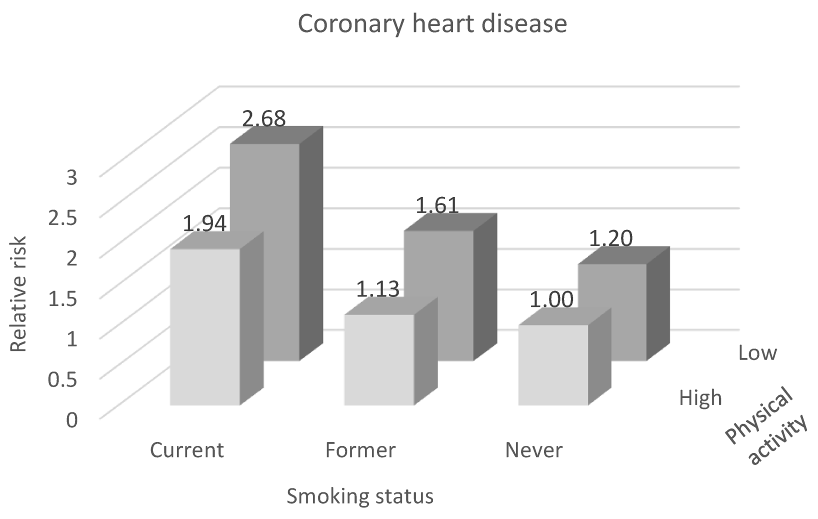
Coronary heart disease
Relative risk
0
0.5
1
1.5
2
2.5
3
1.94
2.68
1.13
1.61
1.00
1.20
Current
Former
Never
Smoking status
Low
High
Physical activity

d

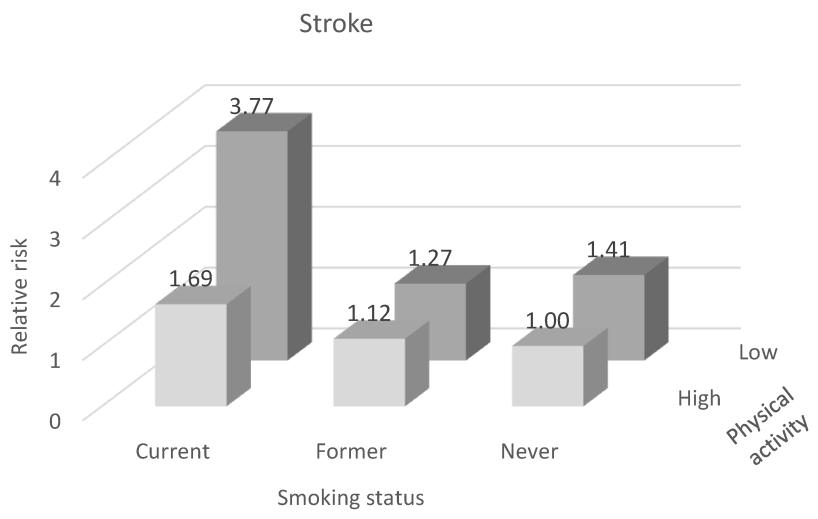
Stroke
Relative risk
0
1
2
3
4
1.69
3.77
1.12
1.27
1.00
1.41
Current
Former
Never
Smoking status
Low
High
Physical activity

e

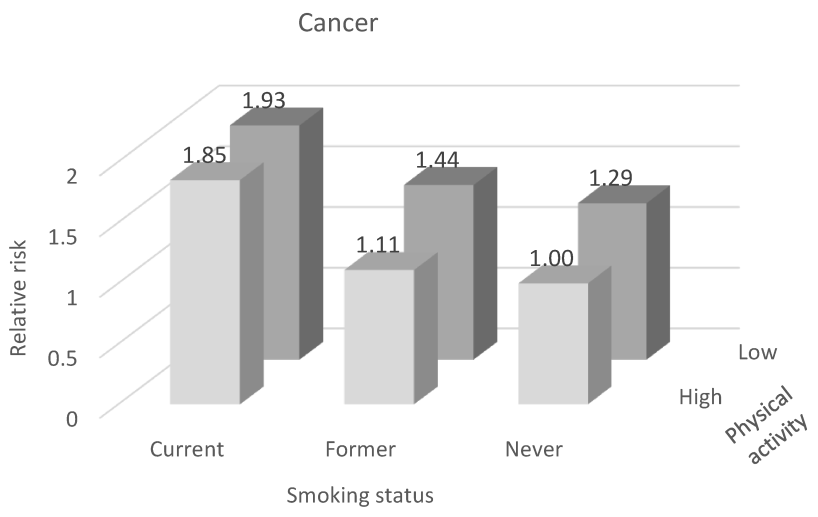
Cancer
Relative risk
0
0.5
1
1.5
2
1.85
1.93
1.11
1.44
1.00
1.29
Current
Former
Never
Smoking status
Low
High
Physical activity

f

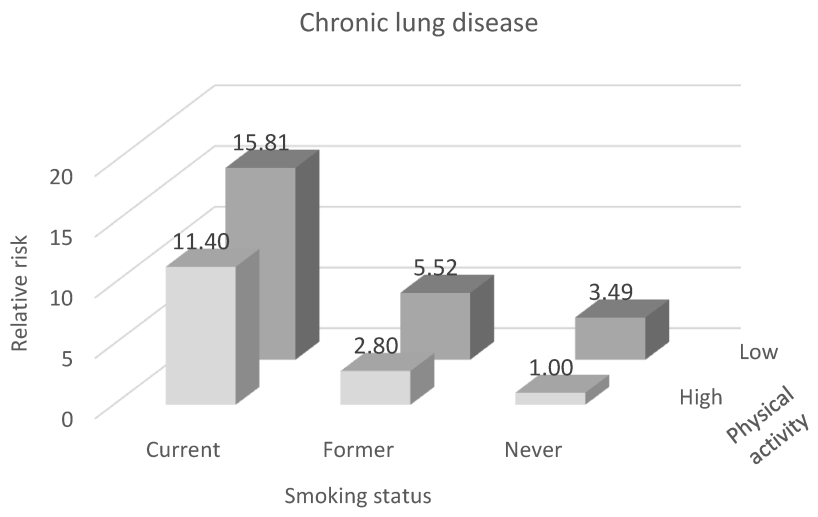
Chronic lung disease
Relative risk
0
5
10
15
20
11.40
15.81
2.80
5.52
1.00
3.49
Current
Former
Never
Smoking status
Low
High
Physical activity

g

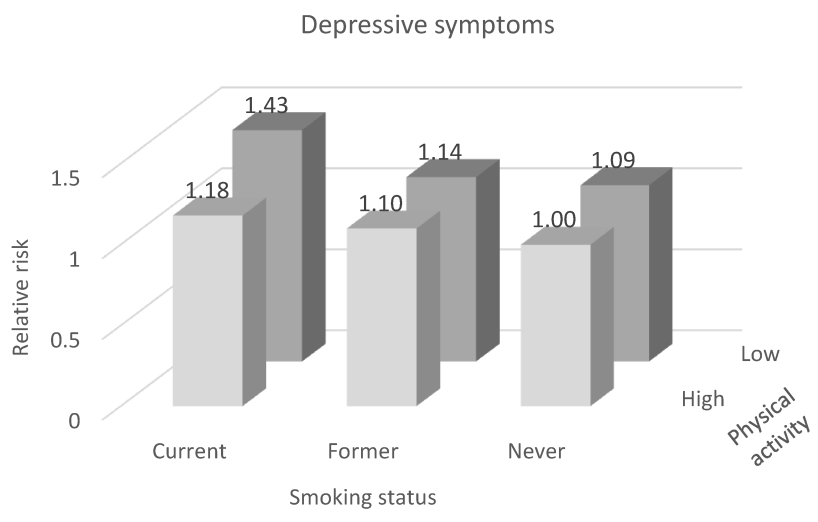
Depressive symptoms
Relative risk
0
0.5
1
1.5
1.18
1.43
1.10
1.14
1.00
1.09
Current
Former
Never
Smoking status
Low
High
Physical activity

Supplementary Figure 3. Relative risks of developing (a) fair/poor self-rated health, (b) limiting long-standing illness, (c) coronary heart disease, (d) stroke, (e) cancer, (f) chronic lung disease, and (g) clinically relevant depressive symptoms over 12-year follow-up by baseline smoking/physical activity status, among older adults free of these conditions at baseline: excluding current smokers with low levels of nicotine dependence (<15 cigarettes/day)
