# Supplementary material for: Combined health risks of cigarette smoking and low levels of physical activity: a prospective cohort study in England with 12-year follow-up
Source: BMJ Open. 2019 Nov 27;9(11):e032852. doi: 10.1136/bmjopen-2019-032852 (PMC6887020; doi:10.1136/bmjopen-2019-032852)
Supplement: Author's manuscript [file bmjopen-2019-032852.draft_revisions.pdf]

BMJ Open is committed to open peer review. As part of this commitment we make the peer review history of every article we publish publicly available.

When an article is published we post the peer reviewers' comments and the authors' responses online. We also post the versions of the paper that were used during peer review. These are the versions that the peer review comments apply to.

The versions of the paper that follow are the versions that were submitted during the peer review process. They are not the versions of record or the final published versions. They should not be cited or distributed as the published version of this manuscript.

BMJ Open is an open access journal and the full, final, typeset and author-corrected version of record of the manuscript is available on our site with no access controls, subscription charges or pay-per-view fees (http://bmjopen.bmj.com).

If you have any questions on BMJ Open's open peer review process please email info.bmjopen@bmj.com

BMJ Open

The combined health risks of cigarette smoking and low levels of physical activity: a prospective cohort study with 12-year follow-up

Journal:	*BMJ Open*
Manuscript ID	bmjopen-2019-032852
Article Type:	Research
Date Submitted by the Author:	11-Jul-2019
Complete List of Authors:	Jackson, Sarah ; University College London, Epidemiology and Public Health;<br>Brown, Jamie; University College London, Psychology & Language Sciences<br>Ussher, Michael; St George's University of London, Division of Population Health Sciences and Education<br>Shahab, Lion; UCL, Dept of Epidemiology and Public Health<br>Steptoe, Andrew; University College London, Epidemiology and Public Health<br>Smith, Lee; Anglia Ruskin University - Cambridge Campus, Department of Life Sciences
Keywords:	smoking, physical activity, cancer, cardiovascular disease, lung disease, depresison

SCHOLARONE™
Manuscripts

The combined health risks of cigarette smoking and low levels of physical activity: a prospective cohort study with 12-year follow-up

Sarah E. Jackson[1], Jamie Brown[1,2], Michael Ussher[3,4], Lion Shahab[1], Andrew Steptoe[1], Lee Smith[5]

[1] Department of Behavioural Science and Health, University College London, London, UK

[2] Department of Clinical, Educational and Health Psychology, University College London, UK

[3] Population Health Research Institute, St George's, University of London, London, UK

[4] Institute of Social Marketing and Health, University of Stirling, Stirling, UK

[5] The Cambridge Centre for Sport and Exercise Sciences, Anglia Ruskin University, UK

Corresponding author: Dr Sarah Jackson, Department of Behavioural Science and Health, University College London, 1-19 Torrington Place, London WC1E 7HB, UK. s.e.jackson@ucl.ac.uk +44 (0)207 679 8312

For peer review only

For peer review only - http://bmjopen.bmj.com/site/about/guidelines.xhtml

Abstract

Objectives: To (i) estimate the combined risks of cigarette smoking and physical inactivity for chronic disease, disability, and depressive symptoms, and (ii) determine whether risks associated with these behaviours are additive or synergistic.

Design and setting: Longitudinal observational population study using data from Waves 2 (2004/05) through 8 (2016/17) of the English Longitudinal Study of Ageing, a prospective study of community-dwelling older adults in England.

Participants: 6,425 men and women aged ≥52 years (mean [SD] 65.88 [9.34] years) at baseline.

Main outcome measures: Smoking status (never; former; current) and level of physical activity (high, defined as moderate/vigorous physical activity [MVPA] more than once a week; low, defined as MVPA once a week or less) were self-reported at Wave 2 baseline. Self-rated health, limiting long-standing illness, chronic conditions (coronary heart disease [CHD], stroke, cancer, chronic lung disease), and depressive symptoms were reported in each biennial wave.

Results: Both smoking and low levels of physical activity were associated with increased risk of incident health problems over the 12-year follow-up period. Current smokers with low levels of physical activity had especially high risks of developing fair/poor self-rated health, CHD, stroke, cancer, and chronic lung disease compared with highly active never smokers (RR_{adj} range 1.89-14.00). While additive effects were evident, tests of multiplicative interactions revealed no evidence of large synergistic effects of smoking and low physical activity (Bayes factor range 0.04-0.61), although data were insensitive to detect smaller effects.

Conclusions: Among older adults in England, there were no large synergistic effects of smoking and low levels of physical activity on risk of developing chronic disease or depressive symptoms over 12 years. However, additive effects of smoking and low levels of physical activity were evident, underscoring the importance of each of these lifestyle risk behaviours for disease onset.

Key words: smoking; physical activity; self-rated health; coronary heart disease; stroke; cancer; lung disease; depression

For peer review only - http://bmjopen.bmj.com/site/about/guidelines.xhtml

For peer review only

Strengths and limitations of this study

- This study represents the first effort comprehensively to examine the combined risks of smoking and low levels of physical activity in a large prospective cohort study.
- Adjustment for a range of relevant covariates took into account potential confounders of the associations between exposures and outcomes.
- Findings were robust to three sensitivity analyses taking different analytic approaches.
- Reliance on self-reported data introduced potential for bias.
- We did not model dynamic effects (i.e. the impact of changes in smoking status and physical activity across the time period on disease outcomes) which may have masked some associations.

For peer review only

Introduction

Smoking and low levels of physical activity are among the leading causes of preventable morbidity and mortality worldwide (1). Each is associated with substantially increased risk of developing a host of chronic diseases, including coronary heart disease (CHD), cancer, and chronic lung diseases (2–5). Quitting smoking and taking up physical activity leads to improvements in overall health and longevity, even relatively late in life (2,6–8). Associations between low levels of physical activity and poorer mental health outcomes, including depression and anxiety disorders, have consistently been reported (9,10) and physical inactivity (defined as not meeting the recommended physical activity guidelines for good health) appears to be causally related to mental health conditions (3). The evidence on smoking is mixed, with some studies suggesting that the association with poor mental health can largely be explained by common causes, such as genes that predispose to both smoking and depression (11,12) and others finding evidence for a causal relationship (13,14).

The combined health risks associated with smoking and physical inactivity have not been comprehensively examined. This is important because health risk behaviours tend to cluster within individuals (15–17). Studies in large, representative samples have shown the majority of adults in England and the US have multiple lifestyle risk factors (e.g. smoking, physical inactivity, excessive alcohol intake, low fruit and vegetable consumption) (15,17), and there is evidence to suggest that combinations of lifestyle risk factors have a greater adverse impact on health than would be expected from the added individual effects alone (18–22). If lifestyle risk factors work synergistically (i.e. greater than the sum of the risks associated with each behaviour individually, indicating the behaviours act as effect modifiers for each other) rather than additively (i.e. the combined risk is greater than the individual risks associated with each behaviour) to influence disease risk, there may be potential to increase the public health impact of behaviour change interventions by targeting multiple behaviours (23–25). However, the extant literature on the benefits of multiple behaviour change interventions is mixed, and their effectiveness likely depends upon particular behaviour combinations and whether there is genuine synergy between them (24). A Cochrane review of randomised controlled trials (RCTs) of physical activity in addition to smoking cessation treatment found mixed results, with the majority failing to provide evidence that physical activity aids smoking cessation (26). However, most of these trials had small samples or a physical activity component insufficiently intense to achieve the desired level of activity (26). Examination of the risks associated with smoking and physical activity in combination is important in order to determine synergistic health effects of these risk factors and

For peer review only - http://bmjopen.bmj.com/site/about/guidelines.xhtml

evaluate the potential usefulness of further research targeting this combination of behaviours in interventions for primary prevention.

There is some evidence from cross-sectional studies to suggest smoking and physical activity interact to influence the risk of adverse physical and mental health outcomes. For example, in a large sample of adult smokers, physical activity was found to moderate the association between nicotine dependence and depression (27). The results indicated smokers with high nicotine dependence and low physical activity were more likely to be depressed than would be expected on the basis of individual effects of smoking and physical inactivity. Similarly, a survey of undergraduate smokers found that those with a lower level of physical activity had higher odds of depression (28). However, the cross-sectional study design makes it very difficult to interpret the direction of associations. For example, it is possible being depressed leads to the uptake of smoking and a loss of interest in physical activity, as opposed to being the result of these behaviours. A number of RCTs have examined the impact of physical activity on smoking and cessation outcomes, and provided strong evidence exercise reduces nicotine cravings and withdrawal symptoms (26,29,30), although a positive impact on relapse has not clearly been demonstrated (31). Regarding physical health effects, two small experimental studies have examined the impact of physical activity on cardiovascular biomarkers in smokers, and observed improvements in the cardiovascular risk profile over three months (32,33). To the best of our knowledge, no studies have evaluated synergistic effects of smoking and physical activity on depression or chronic disease in a large, representative sample using a prospective design.

Using data collected over 12 years from a large population-based sample of older adults living in England, this study therefore aimed to examine the risks of chronic disease and poor mental health associated with cigarette smoking and low levels of physical activity combined. Specifically, we aimed to answer the following research questions:

1. To what extent is the combination of smoking and low physical activity associated with increased risk of the incidence of poor self-rated health, limiting long-standing illness, CHD, stroke, cancer, chronic lung disease, and depressive symptoms over 12-year follow-up among older adults who are healthy at baseline, over and above the risks associated with smoking or low physical activity alone, or neither smoking nor low physical activity?
2. Are the combined risks of smoking and low physical activity for these outcomes additive or synergistic?

For peer review only - http://bmjopen.bmj.com/site/about/guidelines.xhtml

Method

Design

This investigation used data from the English Longitudinal Study of Ageing (ELSA) covering a 12-year period. ELSA is a population-based longitudinal panel study of a representative sample of men and women aged 50 and older living in England. The study began in 2002 (Wave 1), with participants recruited from an annual cross-sectional survey of households. Data are collected every two years via computer-assisted personal interview and self-completion questionnaires. In alternate (even) waves there is an additional health examination, in which objective measures are obtained. For the present study, baseline data were drawn from Wave 2 (2004/05; the first wave in which height and weight were measured, allowing inclusion of body mass index (BMI) in the analyses), collected when participants were aged ≥52 years. Follow-up data were collected biennially through to Wave 8 (2016/17; the most recent wave of available data).

Measures

Measurement of exposures

Smoking status was defined as current, former, or never smoker on the basis of responses to two yes/no questions: 1) "*Have you ever smoked cigarettes?*" 2) "*Do you smoke cigarettes at all nowadays?*" This measure has been validated against salivary cotinine levels in the Health Survey for England (34).

Physical activity was assessed with three items that asked participants how often they took part in activities that were vigorous (e.g. jogging, cycling), moderately energetic (e.g. gardening, walking at moderate pace), or mildly energetic (e.g. laundry, home repairs). Response options were: more than once a week, once a week, 1-3 times a month, hardly ever/never. Activity examples provided to respondents correspond to metabolic equivalent of task ≥6, ≥3.5 to <6, and ≥2 to <3.5 respectively for vigorous, moderate, and mild activities. For the purpose of analysis, we categorised physical activity into two categories: high physical activity (moderate and/or vigorous activity more than once a week) vs. low physical activity (moderate/vigorous activity once a week or less). This measure has been validated against objective, accelerometer-measured hours of moderate-vigorous intensity activity and demonstrates convergent validity in grading a wide range of psychosocial, physical, and biochemical outcomes (7,35–39).

For peer review only - http://bmjopen.bmj.com/site/about/guidelines.xhtml

Measurement of outcomes

We included as outcomes two measures of subjective health (self-rated health, limiting long-standing illness), four diagnosed chronic conditions (CHD, stroke, cancer, chronic lung disease), and one measure of mental health (clinically relevant depressive symptoms). Full details of these measures are provided in the Supplementary Material. For each outcome of interest, we analysed the proportion of participants free from that outcome at Wave 2 baseline who reported the presence of the outcome in Wave 3, 4, 5, 6, 7 or 8 (coded 1). Therefore, our dependent variables incorporated all new-onset adverse health outcomes reported by participants across the 12-year follow-up period. For our primary analyses, participants retained in the study at Wave 8 who did not report the presence of the outcome in any wave were coded 0. Participants lost to follow-up before Wave 8 who did not report the presence of the outcome in any wave were coded as missing, because it was not possible to determine their status.

Measurement of covariates

Demographic variables included baseline age, sex, ethnicity (white vs. non-white), and household non-pension wealth. Past-year alcohol intake was categorised as never/rare (never – once or twice a year), regular but infrequent (once every couple of months – twice a week), or frequent (3 days a week – almost every day). BMI was calculated as weight in kilograms/(height in metres)^2 based on objective measurements.

Statistical analysis

The analysis plan was pre-registered on Open Science Framework (https://osf.io/g9p2b/). We amended our pre-specified definition of physical activity categories upon seeing the distribution of the data, because our original dichotomy of moderate/vigorous physical activity at least once a week resulted in an implausibly high proportion of the sample being classified as high active (~80%). For transparency, results based on the original categorisation are available on Open Science Framework.

We used one-way independent analyses of variance (ANOVA; continuous variables) and Pearson's chi-square tests (categorical variables) to analyse differences in baseline characteristics by smoking status (never/former/current) and level of physical activity (high/low).

We used log-binomial regression to calculate the relative risks (RR) with 95% confidence intervals (CIs) associated with smoking and physical activity of incident fair/poor self-rated health, limiting long-standing

For peer review only - http://bmjopen.bmj.com/site/about/guidelines.xhtml

illness, CHD, stroke, cancer, chronic lung disease, and depressive symptoms over 12-year follow-up among participants who did not report the outcome of interest at baseline. We constructed five models for each outcome. The first and second calculated unadjusted RRs associated with smoking status (reference category: never smoker) and physical activity (reference category: high active), respectively. The third tested main effects of smoking status and physical activity, and the multiplicative interaction between smoking status and physical activity, controlling for covariates. The fourth and fifth calculated unadjusted and adjusted RRs, respectively, associated with each combination of smoking status and level of physical activity: (i) never smoker/high active (reference category); (ii) never smoker/low active; (iii) former smoker/high active; (iv) former smoker/low active; (v) current smoker/high active; and (vi) current smoker/low active.

We performed three sensitivity analyses. The first imputed missing outcomes data for those who dropped out of ELSA before Wave 8 and did not report the presence of any of these conditions in their completed waves. A multiple imputation model was run with all exposures and covariates entered as predictors. Five imputed datasets were created, each was analysed separately, and the results were combined to produce pooled estimates of effects. The second sensitivity analysis restricted the sample to those with complete data at Wave 2 and Wave 8 to assess healthy survivor effects. The third excluded current smokers who smoke <15 cigarettes per day (indicative of a lower level of nicotine dependence) in order to address the potential issue of differential rates of smoking cessation in relation to level of physical activity (40). One would expect a lower rate of successful quitting during the follow-up period among more dependent smokers, so it was thought that excluding those who were less dependent may provide a better reflection of the combined health risks of smoking and low physical activity rather than an artefact of more successful quitting among active smokers generally.

In order to evaluate the extent to which our data supported the null hypothesis (i.e. no synergistic relationship between smoking and physical activity for risk of incident health problems), the experimental hypothesis (i.e. synergy between smoking and physical activity), or were insensitive, we calculated Bayes factors (BFs) for the multiplicative interaction results (see Supplementary Material for details).

All analyses were conducted in SPSS v.24, with the exception of the BFs which were calculated using an online calculator (http://www.lifesci.sussex.ac.uk/home/Zoltan_Dienes/inference/Bayes.htm).

Public and patient involvement

For peer review only - http://bmjopen.bmj.com/site/about/guidelines.xhtml

No patients were involved in setting the research questions or outcome measures, nor were they involved in the design and implementation of the study. There are no plans to involve patients in dissemination.

Results

Sample characteristics

There were 9,432 individuals in Wave 2 of ELSA, of whom 7,666 (81.3%) participated in the health examination in which objective measurements of height and weight were obtained. We excluded 1,241 individuals (16.2%) with missing data, leaving a final sample for analysis of 6,425 participants.

Table 1 presents descriptive characteristics measured at Wave 2 baseline overall and by smoking status and level of physical activity. The sample comprised 2,902 men and 3,523 women aged ≥52 years (mean [SD] 65.88 [9.34] years). Participants were predominantly white (98.8%) and the upper quintiles of wealth were overrepresented. The majority (81.1%) reported regular or frequent alcohol intake and the mean BMI was in the overweight range (27.91 [4.87] kg/m^2). The prevalence of chronic disease and depressive symptoms ranged from 2.4% (stroke) to 32.9% (limiting long-standing illness). Some 14.0% of participants were current smokers, 48.9% were former smokers, and 37.2% were never smokers. Just over a third (34.1%) were classified as having low physical activity.

Associations with incident health problems

For each outcome, Table 2 summarises the unadjusted and adjusted RRs associated with smoking status and physical activity, and interactions between smoking status and physical activity. Table 3 shows the unadjusted and adjusted RRs associated with each combination of smoking status and physical activity.

Main effects of smoking status

In unadjusted models (Table 2), both former and current smokers had significantly higher risks of developing fair/poor self-rated health, CHD, and chronic lung disease than never smokers (RR range 1.20-2.34 for former smokers, RR range 1.45-6.28 for current smokers). Risk of stroke was significantly higher among current smokers than never smokers (RR 1.58), but did not differ significantly between former and

For peer review only - http://bmjopen.bmj.com/site/about/guidelines.xhtml

never smokers (RR 1.22). Smoking status was not significantly associated with risk of developing a limiting long-standing illness, cancer, or clinically relevant depressive symptoms (RR range 1.10-1.28).

After adjustment for age, sex, ethnicity, wealth, alcohol intake, BMI, and level of physical activity (Table 2), the risk of developing chronic lung disease remained significantly higher among former (RR_{adj} 2.77) and current smokers (RR_{adj} 8.33), and risks of developing fair/poor self-rated health, CHD, and stroke were significantly higher among current smokers (RR_{adj} range 1.55-1.93), relative to never smokers. The risk of developing cancer approached statistical significance for current versus never smokers (RR_{adj} 1.44).

Main effects of physical activity

In unadjusted models (Table 2), participants with low physical activity had significantly higher risks of developing fair/poor self-rated health, limiting long-standing illness, CHD, stroke, cancer, chronic lung disease, and clinically relevant depressive symptoms than those with high physical activity (RR range 1.19-2.67).

After adjustment for age, sex, ethnicity, wealth, alcohol intake, BMI, and smoking status (Table 2), the risk of developing chronic lung disease remained significantly higher among those with low versus high physical activity (RR_{adj} 3.50), but other associations were attenuated and became non-significant (RR_{adj} range 1.06-1.40).

Additive and synergistic effects of smoking status and physical activity

After adjustment for covariates, significant differences in risks of developing fair/poor self-rated health, CHD, stroke, cancer, and chronic lung disease were observed across different combinations of smoking status and levels of physical activity (Table 3, Figure 1).

Relative to never smokers with high physical activity, current smokers with low physical activity had the highest risks of each of these outcomes (RR_{adj} range 1.89-14.00). Risks of fair/poor self-rated health, CHD, stroke, and chronic lung disease were also significantly elevated among current smokers with high physical activity (RR_{adj} range 1.55-8.33), and the risk of cancer approached significance (RR_{adj} 1.44), although relative risks were lower than those for current smokers with low physical activity.

Risks of fair/poor self-rated health, CHD, cancer, and chronic lung disease were also significantly elevated for those with low physical activity who had stopped smoking, although risks relative to never smokers with

For peer review only - http://bmjopen.bmj.com/site/about/guidelines.xhtml

high physical activity were comparatively lower than were observed for current smokers (RR_{adj} range 1.35-5.42). Chronic lung disease was the only outcome for which significantly elevated risk was observed among former smokers with high physical activity (RR_{adj} 2.77) or never smokers with low physical activity (RR_{adj} 3.50), relative to never smokers with high physical activity.

The risks of limiting long-standing illness and clinically relevant depressive symptoms did not differ significantly across smoking/physical activity groups, although point estimates followed a similar pattern (Table 3, Figure 1).

While additive effects were evident, with the health risks associated with the combination of current smoking and low physical activity higher than those associated with one or other of these behaviours in isolation (Table 3, Figure 1), tests of multiplicative interactions revealed no evidence of synergistic effects of smoking and low physical activity (Table 2). The only outcome for which the interaction approached statistical significance was chronic lung disease (p=0.070), where the effect was in the opposite direction to what we hypothesised, i.e. relative to never smokers, the increase in risk associated with inactivity appears smaller in current smokers.

BFs based on large synergistic effects between smoking status and physical activity indicated the data provided strong evidence for the null hypothesis for chronic lung disease and moderate evidence for the null hypothesis for incident fair/poor self-rated health, limiting long-standing illness, cancer, and depressive symptoms, but were insensitive to detect large effects for CHD and stroke (Supplementary Table 1). BFs based on medium and small synergistic effects favoured the null hypothesis but indicated the data were insensitive for all outcomes except chronic lung disease.

Sensitivity analyses

Sensitivity analyses taking three different analytic approaches produced a very similar pattern of results (see Supplementary Material, Supplementary Tables 2-4 and Supplementary Figures 1-3 for full details).

Discussion

In this large prospective study of older adults, we examined the risks of incident self-rated health, limiting long-standing illness, CHD, stroke, cancer, chronic lung disease, and depressive symptoms over 12-year

For peer review only - http://bmjopen.bmj.com/site/about/guidelines.xhtml

follow-up associated with smoking and low levels of physical activity among individuals free of these conditions at baseline. We observed additive effects of smoking and low physical activity on these outcomes, with older adults who reported both current smoking and low physical activity at higher risk of developing these conditions than those who engaged in one or neither of these lifestyle risk behaviours. However, there was no evidence of synergistic effects of smoking and low physical activity on the incidence of these conditions.

It has been proposed that targeting multiple behaviours could increase the public health impact of behaviour change interventions (23–25), but evidence on the effectiveness of this strategy is inconsistent (24). For example, studies focusing on physical activity and diet have shown interventions that focus on a single behaviour are more effective in increasing the target behaviours, while those that target both behaviours result in greater weight loss (41). Dieting while trying to stop smoking is associated with worse smoking outcomes (42), and it is generally recommended smokers do not attempt to diet until several months after quitting (42). It is likely that the effectiveness of multiple behaviour change interventions relies on there being a synergistic relationship between the target behaviours. The failure of the present study to find evidence of synergy between smoking and low physical activity on risk of chronic disease and depressive symptoms suggests targeting this combination of behaviours is unlikely to be more effective in reducing the risk of these adverse health outcomes than focusing on each behaviour separately. This is consistent with findings of RCTs that have examined effects of physical activity as an adjunct to smoking cessation treatment. A 2014 Cochrane review (26) identified 20 RCTs (total n=5,870) that compared an exercise-only intervention or a combined exercise and smoking cessation intervention with a cessation only intervention. Just two of the 20 trials found a beneficial effect of including an exercise component on long-term cessation (26).

However, despite the lack of evidence for synergy between these behaviours, there are other reasons why targeting smoking and physical activity in a multiple behaviour change intervention may be beneficial. For example, changes in physical activity as a result of an intervention may interact differently with smoking compared with more spontaneous changes in physical activity (as reported in cohort studies) and especially so if the intervention is actively used to promote cessation (e.g. as a means for reducing cigarette cravings (30)). It is also possible that smoking and physical activity may interact in different ways depending on the timing of changes in the two behaviours (43).

For peer review only - http://bmjopen.bmj.com/site/about/guidelines.xhtml

While the present results provide no evidence for synergistic effects of smoking and low physical activity on health, there were clear additive effects. Current smokers were at higher risk of incident health problems than former or never smokers. People with low physical activity were at higher risk of incident health problems than those who engaged in regular moderate/vigorous intensity physical activity. The combination of current smoking and low physical activity conferred the highest risk of each outcome: notably, individuals who reported both behaviours had more than twice the risk of developing CHD, three times higher risk of having a stroke, and 14 times higher risk of developing chronic lung disease over 12-year follow-up than never smokers who engaged in regular physical activity. These results emphasise the importance of promoting both abstinence from smoking and regular physical activity, and intervening to encourage behaviour change for people with unhealthy lifestyles.

This study had several strengths. The sample was drawn from a large, nationally-representative cohort of older adults. The prospective design facilitated assessment of the temporal relationship between smoking and physical inactivity and future disease onset. Assuming the health risk behaviours have a cumulative (dose-response) effect on health outcomes, the older age of the sample meant we had a better chance of detecting an effect given longer exposure in this population group. Adjustment for a range of relevant covariates took into account potential confounders of the associations between exposures and outcomes. Findings were robust to three sensitivity analyses taking different analytic approaches.

There were also a number of limitations. First, the items used to assess smoking status did not specify regular smoking, meaning the group of former smokers encompassed a wide range of smoking histories, from very occasional use to heavy smoking. As such, our results may underestimate the health risks associated with former (regular) smoking. Second, physical activity was self-reported, introducing scope for bias. A recent study documented notable discrepancy between objective measures and self-reports of physical activity, including an age-related decline in activity levels captured by accelerometery that was not observed in self-reports (44). Third, chronic disease outcomes were based on self-reports of doctor diagnosis, and it is possible some may have been forgotten or not reported. However, validation studies comparing self-reports against medical records generally show high agreement (45). Fourth, while we included participants who reported the onset of health problems in any wave, regardless of whether they were retained in ELSA through to final follow-up at Wave 8, we excluded from our primary analyses those who did not report health problems or depressive symptoms prior to dropout. This group likely included individuals suffering from the conditions we were studying, but who died before the diseases were identified or could be reported in an ELSA interview. As such, our results may underestimate the impact of

For peer review only - http://bmjopen.bmj.com/site/about/guidelines.xhtml

our exposures on the health outcomes of interest, although a sensitivity analysis based on imputed data produced similar estimates of associations. In addition, we did not model dynamic effects (i.e. the impact of changes in smoking status and physical activity across the time period on disease outcomes) which may have masked some associations. Fifth, although we had a large sample, the number of incident diagnoses was relatively small meaning we likely lacked statistical power to detect significant effects. Indeed, Bayes factors indicated that while the data supported the null hypothesis (i.e. no synergistic effects of smoking and physical activity), there was some data insensitivity which meant we were unable to rule out small and medium-sized effects. Finally, while we adjusted for a range of potential confounders, there were no data available on substance misuse (aside from alcohol intake, which we controlled for) or diet quality. These variables have been associated to varying degrees with our exposures (15–17,46) and outcomes of interest (47–50). Further research is required to validate our findings with adjustment for these variables.

Conclusions

The present results are not suggestive of large synergistic effects of smoking and low levels of physical activity on risk of developing chronic disease or clinically relevant depressive symptoms (although smaller synergistic effects cannot be ruled out). However, additive effects of smoking and low activity were evident, underscoring the importance of each of these behaviours for disease onset.

For peer review only

List of abbreviations

ANOVA – analysis of variance

BF – Bayes factor

BMI – body mass index

CHD – coronary heart disease

CI – confidence interval

ELSA – English Longitudinal Study of Ageing

RCT – randomised controlled trial

RR – relative risk

For peer review only

For peer review only - http://bmjopen.bmj.com/site/about/guidelines.xhtml

Declarations

Ethics approval and consent to participate

Ethical approval for the English Longitudinal Study of Ageing was provided by the London Multi-Centre Research Ethics Committee (MREC/01/2/91) and participants provided full informed consent.

Availability of data and material

The raw ELSA data are freely available to download from the UK Data Service.

Competing interests

J Brown has received unrestricted research funding from Pfizer, who manufacture smoking cessation medications. L Shahab has received a research grant and honoraria for a talk and travel expenses from manufacturers of smoking cessation medications (Pfizer and Johnson & Johnson). All authors declare no financial links with tobacco companies or e-cigarette manufacturers or their representatives. All other authors declare no competing interests.

Funding

This work was supported by Cancer Research UK (C1417/A22962) and the Economic and Social Research Council (ES/R005990/1). The funders had no final role in the study design; in the collection, analysis and interpretation of data; in the writing of the report; or in the decision to submit the paper for publication. All researchers listed as authors are independent from the funders and all final decisions about the research were taken by the investigators and were unrestricted. All authors had full access to all of the data (including statistical reports and tables) in the study and can take responsibility for the integrity of the data and the accuracy of the data analysis.

Authors' contributions

SE Jackson and L Smith conceived the study. All authors contributed to the study design. SE Jackson analysed and interpreted the data, and drafted the manuscript. All authors revised the manuscript critically for intellectual content, and read and approved the final manuscript.

For peer review only - http://bmjopen.bmj.com/site/about/guidelines.xhtml

References

1. Lopez AD, Mathers CD, Ezzati M, Jamison DT, Murray CJ, editors. Global Burden of Disease and Risk Factors [Internet]. Washington (DC): World Bank; 2006 [cited 2018 Oct 2]. Available from: http://www.ncbi.nlm.nih.gov/books/NBK11812/

2. Warburton DER, Nicol CW, Bredin SSD. Health benefits of physical activity: the evidence. Can Med Assoc J. 2006 Mar 14;174(6):801–9.

3. Penedo FJ, Dahn JR. Exercise and well-being: a review of mental and physical health benefits associated with physical activity. Curr Opin Psychiatry. 2005 Mar;18(2):189.

4. U.S. Department of Health and Human Services. The Health Consequences of Smoking—50 Years of Progress: A Report of the Surgeon General. Atlanta, GA: U.S. Department of Health and Human Services, Centers for Disease Control and Prevention, National Center for Chronic Disease Prevention and Health Promotion, Office on Smoking and Health; 2014.

5. Mokdad AH, Marks JS, Stroup DF, Gerberding JL. Actual causes of death in the United States, 2000. Jama. 2004;291(10):1238–1245.

6. Anthonisen NR, Skeans MA, Wise RA, Manfreda J, Kanner RE, Connett JE. The Effects of a Smoking Cessation Intervention on 14.5-Year Mortality: A Randomized Clinical Trial. Ann Intern Med. 2005 Feb 15;142(4):233–9.

7. Hamer M, Lavoie KL, Bacon SL. Taking up physical activity in later life and healthy ageing: the English longitudinal study of ageing. Br J Sports Med. 2014 Feb 1;48(3):239–43.

8. Jha P, Ramasundarahettige C, Landsman V, Rostron B, Thun M, Anderson RN, et al. 21st-Century Hazards of Smoking and Benefits of Cessation in the United States. N Engl J Med. 2013 Jan 24;368(4):341–50.

9. Hallgren M, Nguyen T-T-D, Herring MP, McDowell CP, Gordon BR, Stubbs B, et al. Associations of physical activity with anxiety symptoms and disorders: Findings from the Swedish National March Cohort. Gen Hosp Psychiatry. 2019 Mar 10;58:45–50.

10. Schuch FB, Vancampfort D, Firth J, Rosenbaum S, Ward PB, Silva ES, et al. Physical Activity and Incident Depression: A Meta-Analysis of Prospective Cohort Studies. Am J Psychiatry. 2018 Jul 1;175(7):631–48.

11. Kendler KS, Neale MC, MacLean CJ, Heath AC, Eaves LJ, Kessler RC. Smoking and Major Depression: A Causal Analysis. Arch Gen Psychiatry. 1993 Jan 1;50(1):36–43.

12. Fergusson DM, Goodwin RD, Horwood LJ. Major depression and cigarette smoking: results of a 21-year longitudinal study. Psychol Med. 2003 Nov;33(8):1357–67.

13. Boden JM, Fergusson DM, Horwood LJ. Cigarette smoking and depression: tests of causal linkages using a longitudinal birth cohort. Br J Psychiatry. 2010 Jun;196(6):440–6.

For peer review only - http://bmjopen.bmj.com/site/about/guidelines.xhtml

14. Wootton RE, Richmond RC, Stuijfzand BG, Lawn RB, Sallis HM, Taylor GMJ, et al. Causal effects of lifetime smoking on risk for depression and schizophrenia: Evidence from a Mendelian randomisation study. bioRxiv. 2018 Dec 20;381301.

15. Poortinga W. The prevalence and clustering of four major lifestyle risk factors in an English adult population. Prev Med. 2007 Feb 1;44(2):124–8.

16. Chiolero A, Wietlisbach V, Ruffieux C, Paccaud F, Cornuz J. Clustering of risk behaviors with cigarette consumption: A population-based survey. Prev Med. 2006 May 1;42(5):348–53.

17. Berrigan D, Dodd K, Troiano RP, Krebs-Smith SM, Barbash RB. Patterns of health behavior in U.S. adults. Prev Med. 2003 May 1;36(5):615–23.

18. Marrero JA, Fontana RJ, Fu S, Conjeevaram HS, Su GL, Lok AS. Alcohol, tobacco and obesity are synergistic risk factors for hepatocellular carcinoma. J Hepatol. 2005 Feb 1;42(2):218–24.

19. Freedman DM, Sigurdson AJ, Rajaraman P, Doody MM, Linet MS, Ron E. The Mortality Risk of Smoking and Obesity Combined. Am J Prev Med. 2006 Nov;31(5):355–62.

20. Li TY, Rana JS, Manson JE, Willett WC, Stampfer MJ, Colditz GA, et al. Obesity as Compared With Physical Activity in Predicting Risk of Coronary Heart Disease in Women. Circulation. 2006 Jan 31;113(4):499–506.

21. Slattery ML, Potter JD. Physical activity and colon cancer: confounding or interaction? Med Sci Sports Exerc. 2002 Jun;34(6):913–9.

22. Schlecht NF, Franco EL, Pintos J, Negassa A, Kowalski LP, Oliveira BV, et al. Interaction between Tobacco and Alcohol Consumption and the Risk of Cancers of the Upper Aero-Digestive Tract in Brazil. Am J Epidemiol. 1999 Dec 1;150(11):1129–37.

23. Prochaska JJ, Prochaska JO. A Review of Multiple Health Behavior Change Interventions for Primary Prevention. Am J Lifestyle Med. 2011 May 1;5(3):208–21.

24. Spring B, Moller AC, Coons MJ. Multiple health behaviours: overview and implications. J Public Health Oxf Engl. 2012 Mar;34 Suppl 1:i3-10.

25. Alageel S, Gulliford MC, McDermott L, Wright AJ. Multiple health behaviour change interventions for primary prevention of cardiovascular disease in primary care: systematic review and meta-analysis. BMJ Open. 2017 Jun 1;7(6):e015375.

26. Ussher MH, Taylor AH, Faulkner GEJ. Exercise interventions for smoking cessation. Cochrane Database Syst Rev [Internet]. 2014 [cited 2019 Mar 15];(8). Available from: https://www.cochranelibrary.com/cdsr/doi/10.1002/14651858.CD002295.pub5/full

27. Loprinzi PD, Walker JF, Kane C, Cardinal BJ. Physical activity moderates the association between nicotine dependence and depression among U.S. smokers. Am J Health Promot AJHP. 2014 Oct;29(1):37–42.

For peer review only

For peer review only - http://bmjopen.bmj.com/site/about/guidelines.xhtml

28. Vickers KS, Patten CA, Lane K, Clark MM, Croghan IT, Schroeder DR, et al. Depressed versus nondepressed young adult tobacco users: differences in coping style, weight concerns and exercise level. Health Psychol Off J Div Health Psychol Am Psychol Assoc. 2003 Sep;22(5):498–503.

29. Roberts V, Maddison R, Simpson C, Bullen C, Prapavessis H. The acute effects of exercise on cigarette cravings, withdrawal symptoms, affect, and smoking behaviour: systematic review update and meta-analysis. Psychopharmacology (Berl). 2012 Jul;222(1):1–15.

30. Haasova M, Warren FC, Ussher M, Janse Van Rensburg K, Faulkner G, Cropley M, et al. The acute effects of physical activity on cigarette cravings: systematic review and meta-analysis with individual participant data. Addict Abingdon Engl. 2013 Jan;108(1):26–37.

31. Bernard P, Ninot G, Cyprien F, Courtet P, Guillaume S, Georgescu V, et al. Exercise and Counseling for Smoking Cessation in Smokers With Depressive Symptoms: A Randomized Controlled Pilot Trial. J Dual Diagn. 2015;11(3–4):205–16.

32. Korhonen T, Goodwin A, Miesmaa P, Dupuis EA, Kinnunen T. Smoking cessation program with exercise improves cardiovascular disease biomarkers in sedentary women. J Womens Health 2002. 2011 Jul;20(7):1051–64.

33. Koubaa A, Triki M, Trabelsi H, Baati H, Sahnoun Z, Hakim A. The effect of a 12-week moderate intensity interval training program on the antioxidant defense capability and lipid profile in men smoking cigarettes or hookah: a cohort study. ScientificWorldJournal. 2015;2015:639369.

34. Erens B, Primatesta P. Health survey for England 2000: cardiovascular disease. Lond Station Off. 2001;

35. Demakakos P, Hamer M, Stamatakis E, Steptoe A. Low-intensity physical activity is associated with reduced risk of incident type 2 diabetes in older adults: evidence from the English Longitudinal Study of Ageing. Diabetologia. 2010 May 22;53:1877–85.

36. Hamer M, Stamatakis E. Screen-Based Sedentary Behavior, Physical Activity, and Muscle Strength in the English Longitudinal Study of Ageing. PLOS ONE. 2013 Jun 3;8(6):e66222.

37. Hamer M, Stamatakis E. Prospective Study of Sedentary Behavior, Risk of Depression, and Cognitive Impairment. Med Sci Sports Exerc. 2014 Apr 1;46(4):718–23.

38. Hamer M, Molloy GJ, de Oliveira C, Demakakos P. Leisure time physical activity, risk of depressive symptoms, and inflammatory mediators: the English Longitudinal Study of Ageing. Psychoneuroendocrinology. 2009 Aug;34(7):1050–5.

39. Hamer M, de Oliveira C, Demakakos P. Non-exercise physical activity and survival: English longitudinal study of ageing. Am J Prev Med. 2014 Oct;47(4):452–60.

40. Loprinzi PD, Walker JF. Nicotine Dependence, Physical Activity, and Sedentary Behavior among Adult Smokers. North Am J Med Sci. 2015 Mar;7(3):94–9.

41. Sweet SN, Fortier MS. Improving Physical Activity and Dietary Behaviours with Single or Multiple Health Behaviour Interventions? A Synthesis of Meta-Analyses and Reviews. Int J Environ Res Public Health. 2010 Apr;7(4):1720–43.

For peer review only - http://bmjopen.bmj.com/site/about/guidelines.xhtml

42. Perkins KA. Issues in the Prevention of Weight Gain after Smoking Cessation. Ann Behav Med. 1994 Jan 1;16(1):46–52.

43. Hyman DJ, Pavlik VN, Taylor WC, Goodrick GK, Moye L. Simultaneous vs sequential counseling for multiple behavior change. Arch Intern Med. 2007 Jun 11;167(11):1152–8.

44. Kapteyn A, Banks J, Hamer M, Smith JP, Steptoe A, Soest A van, et al. What they say and what they do: comparing physical activity across the USA, England and the Netherlands. J Epidemiol Community Health. 2018 Jun 1;72(6):471–6.

45. Bergmann MM, Byers T, Freedman DS, Mokdad A. Validity of Self-reported Diagnoses Leading to Hospitalization: A Comparison of Self-reports with Hospital Records in a Prospective Study of American Adults. Am J Epidemiol. 1998 May 15;147(10):969–77.

46. Kelly PJ, Baker AL, Deane FP, Kay-Lambkin FJ, Bonevski B, Tregarthen J. Prevalence of smoking and other health risk factors in people attending residential substance abuse treatment. Drug Alcohol Rev. 2012;31(5):638–44.

47. Quirk SE, Williams LJ, O'Neil A, Pasco JA, Jacka FN, Housden S, et al. The association between diet quality, dietary patterns and depression in adults: a systematic review. BMC Psychiatry. 2013 Jun 27;13(1):175.

48. Mente A, Koning L de, Shannon HS, Anand SS. A Systematic Review of the Evidence Supporting a Causal Link Between Dietary Factors and Coronary Heart Disease. Arch Intern Med. 2009 Apr 13;169(7):659–69.

49. Donaldson MS. Nutrition and cancer: A review of the evidence for an anti-cancer diet. Nutr J. 2004 Oct 20;3(1):19.

50. Stein MD. MEDICAL CONSEQUENCES OF SUBSTANCE ABUSE. Psychiatr Clin North Am. 1999 Jun 1;22(2):351–70.

For peer review only

For peer review only - http://bmjopen.bmj.com/site/about/guidelines.xhtml

Table 1. Sample characteristics at baseline overall and in relation to smoking status and level of physical activity

		Smoking status				Physical activity		
	Whole sample (*n*=6425)	Never smoker (*n*=2387)	Former smoker (*n*=3141)	Current smoker (*n*=897)	*p*	High (*n*=4233)	Low (*n*=2192)	*p*
Age in years, mean (SD)	65.88 (9.34)	65.55 (9.17)	66.95 (9.60)	63.04 (8.12)	<0.001	64.77 (8.54)	68.03 (10.38)	<0.001
Sex, % (*n*)								
Men	45.2 (2902)	34.1 (813)	53.1 (1667)	47.0 (422)	<0.001	47.5 (2011)	40.6 (891)	<0.001
Women	54.8 (3523)	65.9 (1574)	46.9 (1474)	53.0 (475)	-	52.5 (2222)	59.4 (1301)	-
Ethnicity, % (*n*)								
White	98.8 (6345)	98.1 (2342)	99.3 (3118)	98.7 (885)	0.001	98.7 (4180)	98.8 (2165)	0.944
Non-white	1.2 (80)	1.9 (45)	0.7 (23)	1.3 (12)	-	1.3 (53)	1.2 (27)	-
Wealth quintile, % (*n*)								
1 (poorest)	14.6 (940)	11.5 (275)	13.0 (408)	28.7 (257)	<0.001	10.7 (451)	22.3 (489)	<0.001
2	18.5 (1188)	17.1 (407)	17.8 (559)	24.7 (222)	-	15.6 (661)	24.0 (527)	-
3	20.8 (1338)	21.3 (508)	20.9 (656)	19.4 (174)	-	21.4 (905)	19.8 (433)	-
4	22.3 (1432)	22.9 (546)	23.7 (743)	15.9 (143)	-	24.4 (1034)	18.2 (398)	-
5 (richest)	23.8 (1527)	27.3 (651)	24.7 (775)	11.3 (101)	-	27.9 (1182)	15.7 (345)	-
Alcohol intake, % (*n*)								
Never/rarely	18.9 (1213)	21.5 (513)	14.9 (468)	25.9 (232)	<0.001	15.4 (651)	25.6 (562)	<0.001
Regularly	45.3 (2909)	48.0 (1145)	44.3 (1393)	41.4 (371)	-	45.0 (1905)	45.8 (1004)	-
Frequently	35.8 (2303)	30.5 (729)	40.8 (1280)	32.8 (294)	-	39.6 (1677)	28.6 (626)	-
BMI, mean (SD)	27.91 (4.87)	27.84 (4.84)	28.19 (4.85)	27.14 (4.93)	<0.001	27.48 (4.44)	28.75 (5.51)	<0.001
Fair/poor self-rated health[1], % (*n*)	24.5 (1575)	19.7 (469)	24.5 (770)	37.5 (336)	<0.001	16.8 (709)	39.5 (866)	<0.001
Limiting long-standing illness[1], % (*n*)	32.9 (2111)	28.0 (668)	34.5 (1082)	40.2 (361)	<0.001	23.7 (1004)	50.5 (1107)	<0.001
Coronary heart disease[1], % (*n*)	8.6 (553)	6.7 (159)	10.3 (324)	7.8 (70)	<0.001	6.8 (286)	12.2 (267)	<0.001
Stroke[1], % (*n*)	2.4 (152)	1.6 (39)	2.9 (91)	2.5 (22)	0.009	1.5 (64)	4.0 (88)	<0.001
Cancer[1], % (*n*)	7.7 (496)	7.2 (171)	8.4 (263)	6.9 (62)	0.154	7.2 (303)	8.8 (193)	0.019
Chronic lung disease[1], % (*n*)	7.3 (466)	4.4 (105)	7.7 (241)	13.4 (120)	<0.001	5.4 (228)	10.9 (238)	<0.001
Clinically relevant depressive symptoms[1], % (*n*)	13.5 (860)	11.5 (273)	13.2 (411)	19.8 (176)	<0.001	9.7 (408)	20.8 (452)	<0.001
Smoking status, % (*n*)								
Never smoker	37.2 (2387)	100 (2387)	-	-	-	38.2 (1616)	35.2 (771)	<0.001
Former smoker	48.9 (3141)	-	100 (3141)	-	-	49.3 (2085)	48.2 (1056)	-
Current smoker	14.0 (897)	-	-	100 (897)	-	12.6 (532)	16.7 (365)	-
Level of physical activity, % (*n*)								
High	65.9 (4233)	67.7 (1616)	66.4 (2085)	59.3 (532)	<0.001	100 (4233)	-	-

For peer review only - http://bmjopen.bmj.com/site/about/guidelines.xhtml

Low	34.1 (2192)	32.3 (771)	33.6 (1056)	40.7 (365)	-	-	100 (2192)	-

Unweighted data shown. BMI = body mass index; SD = standard deviation.

[1] Complete data on all health variables at baseline was not a prerequisite for inclusion, so there was a small amount of missing data across these variables. Valid percentages are presented for ease of interpretation.

For peer review only

Table 2. Main effects of smoking status and physical activity and the interaction between smoking status and physical activity for risks of incident health problems over 12-year follow-up

	Smoking status			Physical activity		Interaction[1]	
	Never smoker	Former smoker	Current smoker	High active	Low active	Former smoker x low active	Current smoker x low active
Fair/poor self-rated health							
% (*n*)	40.9 (529)	49.0 (744)	59.2 (225)	42.3 (999)	60.0 (499)	-	-
RR [95% CI]	1	1.20 [1.05-1.37]	1.45 [1.19-1.76]	1	1.42 [1.24-1.62]	-	-
p		0.008	<0.001		<0.001		
RR_{adj} [95% CI]	1	1.14 [0.97-1.35]	1.55 [1.22-1.99]	1	1.19 [0.95-1.49]	0.99 [0.74-1.34]	1.04 [0.68-1.59]
p		0.112	<0.001		0.141	0.954	0.846
Limiting long-standing illness							
% (*n*)	57.1 (720)	62.7 (905)	64.0 (240)	57.8 (1345)	69.2 (520)	-	-
RR [95% CI]	1	1.10 [0.97-1.24]	1.12 [0.93-1.35]	1	1.20 [1.05-1.37]	-	-
p		0.143	0.233		0.007		
RR_{adj} [95% CI]	1	1.07 [0.93-1.24]	1.16 [0.92-1.45]	1	1.06 [0.86-1.31]	0.97 [0.73-1.29]	1.08 [0.71-1.64]
p		0.359	0.205		0.564	0.845	0.719
Coronary heart disease							
% (*n*)	8.8 (117)	11.7 (176)	14.9 (59)	9.2 (214)	15.2 (138)	-	-
RR [95% CI]	1	1.33 [1.04-1.70]	1.69 [1.21-2.36]	1	1.66 [1.32-2.08]	-	-
p		0.023	0.002		<0.001		
RR_{adj} [95% CI]	1	1.13 [0.82-1.55]	1.93 [1.23-3.03]	1	1.19 [0.78-1.82]	1.19 [0.70-2.03]	1.15 [0.57-2.33]
p		0.454	0.004		0.412	0.521	0.704
Stroke							
% (*n*)	8.2 (113)	9.9 (159)	12.9 (54)	8.1 (196)	13.3 (130)	-	-
RR [95% CI]	1	1.22 [0.95-1.56]	1.58 [1.12-2.22]	1	1.64 [1.30-2.07]	-	-
p		0.129	0.009		<0.001		
RR_{adj} [95% CI]	1	1.13 [0.81-1.57]	1.74 [1.06-2.85]	1	1.40 [0.92-2.12]	0.81 [0.47-1.40]	1.28 [0.63-2.64]
p		0.491	0.028		0.115	0.449	0.496

CI = confidence interval; RR = relative risk from bivariate models; RR_{adj} = relative risk adjusted for age, sex, ethnicity, wealth, alcohol intake, body mass index, and physical activity (for smoking status) or smoking status (for physical activity).

[1] Multiplicative interaction between smoking status and physical activity. Results can be interpreted as the difference in RR between high active and low active former/current smokers, relative to the difference in RR between high and low active never smokers. Thus, an interaction term above 1 indicates the disparity between high and low active groups was greater for former/current smokers than never smokers, and an interaction term below 1 indicates the disparity between high and low active groups was smaller for former/current smokers than never smokers.

For peer review only - http://bmjopen.bmj.com/site/about/guidelines.xhtml

Note: For each outcome, the sample is restricted to those who did not report the presence of the outcome at baseline. Results therefore indicate the prevalence and relative risk of new-onset health problems over the follow-up period.

Table 2. (continued)

	Smoking status			Physical activity		Interaction[1]	
	Never smoker	Former smoker	Current smoker	High active	Low active	Former smoker x low active	Current smoker x low active
Cancer							
% (*n*)	13.4 (178)	15.8 (247)	17.1 (70)	13.6 (320)	18.5 (175)	-	-
RR [95% CI]	1	1.18 [0.96-1.45]	1.28 [0.95-1.72]	1	1.36 [1.11-1.66]	-	-
p		0.112	0.107		0.002		
RR_{adj} [95% CI]	1	1.11 [0.86-1.43]	1.44 [0.98-2.13]	1	1.30 [0.92-1.83]	1.01 [0.65-1.57]	1.01 [0.54-1.88]
p		0.445	0.067		0.139	0.970	0.977
Chronic lung disease							
% (*n*)	3.3 (44)	7.8 (120)	20.9 (82)	5.1 (119)	13.7 (127)	-	-
RR [95% CI]	1	2.34 [1.65-3.34]	6.28 [4.28-9.21]	1	2.67 [2.06-3.47]	-	-
p		<0.001	<0.001		<0.001		
RR_{adj} [95% CI]	1	2.77 [1.62-4.74]	8.33 [4.62-15.00]	1	3.50 [1.88-6.52]	0.56 [0.27-1.16]	0.48 [0.22-1.06]
p		<0.001	<0.001		<0.001	0.116	0.070
Clinically relevant depressive symptoms							
% (*n*)	53.5 (418)	56.4 (535)	62.4 (181)	52.8 (714)	62.9 (420)	-	-
RR [95% CI]	1	1.06 [0.90-1.24]	1.17 [0.94-1.46]	1	1.19 [1.02-1.39]	-	-
p		0.512	0.168		0.024		
RR_{adj} [95% CI]	1	1.09 [0.90-1.34]	1.16 [0.87-1.55]	1	1.09 [0.84-1.41]	0.95 [0.68-1.34]	1.07 [0.68-1.69]
p		0.381	0.309		0.511	0.782	0.764

CI = confidence interval; RR = relative risk from bivariate models; RR_{adj} = relative risk adjusted for age, sex, ethnicity, wealth, alcohol intake, body mass index, and physical activity (for smoking status) or smoking status (for physical activity).

[1] Multiplicative interaction between smoking status and physical activity. Results can be interpreted as the difference in RR between high active and low active former/current smokers, relative to the difference in RR between high and low active never smokers. Thus, an interaction term above 1 indicates the disparity between high and low active groups was greater for former/current smokers than never smokers, and an interaction term below 1 indicates the disparity between high and low active groups was smaller for former/current smokers than never smokers.

Note: For each outcome, the sample is restricted to those who did not report the presence of the outcome at baseline. Results therefore indicate the prevalence and relative risk of new-onset health problems over the follow-up period.

For peer review only - http://bmjopen.bmj.com/site/about/guidelines.xhtml

Table 3. Prevalence and unadjusted and adjusted relative risks of incident health problems over 12-year follow-up associated with each smoking/physical activity group

	Never smoker		Former smoker		Current smoker	
	High active	Low active	High active	Low active	High active	Low active
Fair/poor self-rated health						
% (*n*)	37.0 (354)	51.9 (48.1)	44.4 (504)	62.8 (240)	52.8 (141)	74.3 (84)
RR [95% CI]	1	1.40 [1.13-1.75]	1.20 [1.02-1.41]	1.70 [1.39-2.08]	1.43 [1.13-1.81]	2.01 [1.48-2.73]
p		0.002	0.027	<0.001	0.003	<0.001
RR_{adj} [95% CI]	1	1.19 [0.95-1.49]	1.14 [0.97-1.35]	1.35 [1.09-1.66]	1.55 [1.22-1.99]	1.92 [1.40-2.65]
p		0.141	0.112	0.006	<0.001	<0.001
Limiting long-standing illness						
% (*n*)	54.5 (516)	65.2 (204)	60.0 (662)	71.3 (243)	60.1 (167)	75.3 (73)
RR [95% CI]	1	1.20 [0.97-1.47]	1.10 [0.95-1.27]	1.31 [1.08-1.59]	1.10 [0.89-1.37]	1.38 [1.00-1.91]
p		0.089	0.189	0.007	0.384	0.049
RR_{adj} [95% CI]	1	1.06 [0.86-1.31]	1.07 [0.93-1.24]	1.11 [0.91-1.36]	1.16 [0.92-1.45]	1.33 [0.96-1.85]
p		0.564	0.359	0.321	0.205	0.091
Coronary heart disease						
% (*n*)	8.0 (78)	11.0 (39)	9.5 (104)	17.3 (72)	12.3 (32)	19.9 (27)
RR [95% CI]	1	1.38 [0.92-2.06]	1.19 [0.88-1.62]	2.17 [1.55-3.05]	1.53 [0.99-2.37]	2.48 [1.55-3.99]
p		0.119	0.259	<0.001	0.053	<0.001
RR_{adj} [95% CI]	1	1.19 [0.78-1.82]	1.13 [0.82-1.55]	1.60 [1.12-2.30]	1.93 [1.23-3.03]	2.64 [1.59-4.37]
p		0.412	0.454	0.011	0.004	<0.001
Stroke						
% (*n*)	6.7 (67)	12.0 (46)	8.9 (103)	12.6 (56)	9.7 (26)	18.5 (28)
RR [95% CI]	1	1.78 [1.20-2.64]	1.33 [0.96-1.82]	1.88 [1.30-2.73]	1.44 [0.90-2.32]	2.76 [1.72-4.43]
p		0.004	0.083	0.001	0.128	<0.001
RR_{adj} [95% CI]	1	1.40 [0.92-2.12]	1.13 [0.8-1.57]	1.27 [0.86-1.89]	1.74 [1.06-2.85]	3.12 [1.88-5.18
p		0.115	0.491	0.232	0.028	<0.001

CI = confidence interval; RR = relative risk; RR_{adj} = relative risk adjusted for age, sex, ethnicity, wealth, alcohol intake, and body mass index.
Note: For each outcome, the sample is restricted to those who did not report the presence of the outcome at baseline. Results therefore indicate the prevalence and relative risk of new-onset health problems over the follow-up period.

For peer review only - http://bmjopen.bmj.com/site/about/guidelines.xhtml
For peer review only

Table 3. (continued)

	Never smoker		Former smoker		Current smoker	
	High active	Low active	High active	Low active	High active	Low active
Cancer						
% (*n*)	12.4 (120)	16.0 (58)	14.3 (160)	19.8 (87)	15.2 (40)	20.7 (30)
RR [95% CI]	1	1.29 [0.92-1.81]	1.15 [0.89-1.48]	1.60 [1.19-2.15]	1.22 [0.83-1.79]	1.67 [1.08-2.58]
p		0.136	0.276	0.002	0.307	0.022
RR_{adj} [95% CI]	1	1.30 [0.92-1.83]	1.11 [0.86-1.43]	1.45 [1.06-1.97]	1.44 [0.98-2.13]	1.89 [1.20-2.98]
p		0.139	0.445	0.019	0.067	0.006
Chronic lung disease						
% (*n*)	1.9 (18)	7.4 (26)	5.7 (63)	13.2 (57)	15.1 (38)	31.2 (44)
RR [95% CI]	1	3.96 [2.14-7.31]	3.07 [1.80-5.22]	7.07 [4.11-12.16]	8.13 [4.56-14.49]	16.76 [9.42-29.83]
p		<0.001	<0.001	<0.001	<0.001	<0.001
RR_{adj} [95% CI]	1	3.50 [1.88-6.52]	2.77 [1.62-4.74]	5.42 [3.11-9.44]	8.33 [4.62-15.00]	14.00 [7.68-25.53]
p		<0.001	<0.001	<0.001	<0.001	<0.001
Clinically relevant depressive symptoms						
% (*n*)	50.0 (269)	61.1 (149)	53.8 (343)	61.7 (192)	57.6 (102)	69.9 (79)
RR [95% CI]	1	1.22 [0.95-1.57]	1.08 [0.88-1.31]	1.24 [0.98-1.56]	1.15 [0.87-1.53]	1.40 [1.01-1.93]
p		0.118	0.470	0.075	0.328	0.042
RR_{adj} [95% CI]	1	1.09 [0.84-1.41]	1.09 [0.90-1.34]	1.14 [0.89-1.45]	1.16 [0.87-1.55]	1.36 [0.97-1.89]
p		0.511	0.381	0.297	0.309	0.071

CI = confidence interval; RR = relative risk; RR_{adj} = relative risk adjusted for age, sex, ethnicity, wealth, alcohol intake, and body mass index.
Note: For each outcome, the sample is restricted to those who did not report the presence of the outcome at baseline. Results therefore indicate the prevalence and relative risk of new-onset health problems over the follow-up period.

For peer review only

For peer review only - http://bmjopen.bmj.com/site/about/guidelines.xhtml

Figure legends

Figure 1. Relative risks of developing (a) fair/poor self-rated health, (b) limiting long-standing illness, (c) coronary heart disease, (d) stroke, (e) cancer, (f) chronic lung disease, and (g) clinically relevant depressive symptoms over 12-year follow-up by baseline smoking/physical activity status, among older adults free of these conditions at baseline.

For peer review only

For peer review only - http://bmjopen.bmj.com/site/about/guidelines.xhtml

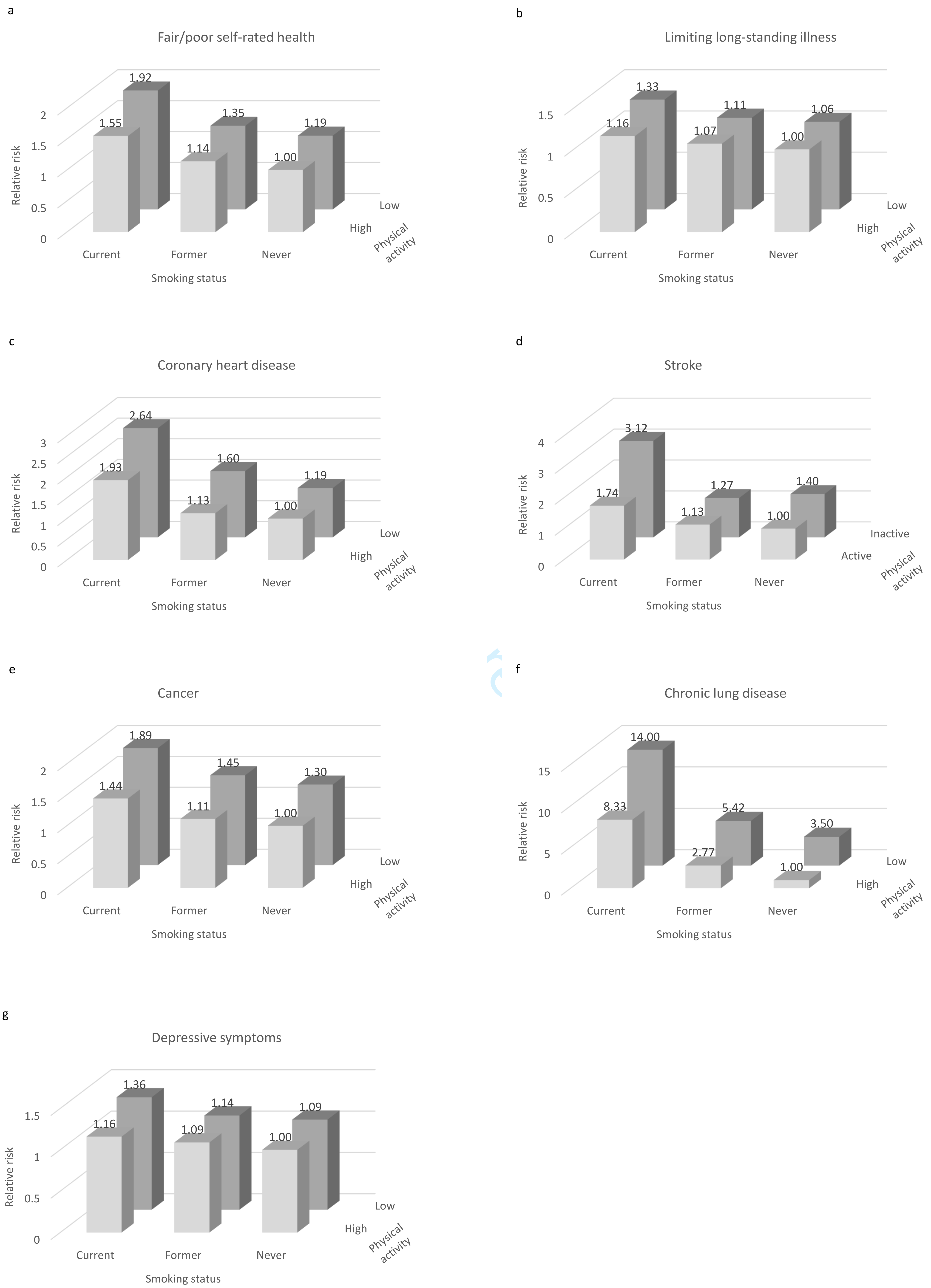
a
Fair/poor self-rated health
1.92
1.55
1.35
1.14
1.19
1.00
Relative risk
0
0.5
1
1.5
2
Current
Former
Never
Smoking status
Low
High
Physical activity
b
Limiting long-standing illness
1.33
1.16
1.11
1.07
1.06
1.00
Relative risk
0
0.5
1
1.5
Current
Former
Never
Smoking status
Low
High
Physical activity
c
Coronary heart disease
2.64
1.93
1.60
1.13
1.19
1.00
Relative risk
0
0.5
1
1.5
2
2.5
3
Current
Former
Never
Smoking status
Low
High
Physical activity
d
Stroke
3.12
1.74
1.27
1.13
1.40
1.00
Relative risk
0
1
2
3
4
Current
Former
Never
Smoking status
Inactive
Active
Physical activity
e
Cancer
1.89
1.44
1.45
1.11
1.30
1.00
Relative risk
0
0.5
1
1.5
2
Current
Former
Never
Smoking status
Low
High
Physical activity
f
Chronic lung disease
14.00
8.33
5.42
2.77
3.50
1.00
Relative risk
0
5
10
15
Current
Former
Never
Smoking status
Low
High
Physical activity
g
Depressive symptoms
1.36
1.16
1.14
1.09
1.09
1.00
Relative risk
0
0.5
1
1.5
Current
Former
Never
Smoking status
Low
High
Physical activity

Supplementary material

Method: measurement of outcomes

Self-rated health was assessed using a single item: *"Would you say your health is… poor/fair/good/very good/excellent?"* We analysed the proportion of individuals rating their health as fair/poor, as has been done in previous investigations (1–3).

We also used data on self-reported limiting long-standing illness, which reflects the extent to which participants feel their daily activities are limited by the presence of illness. This was assessed with two questions: 1) "*Do you have any long-standing illness, disability, or infirmity? By long-standing I mean anything that has troubled you over a period of time or that is likely to affect you over a period of time*." Those who respond yes were asked: 2) "*Does this illness or disability limit your activities in any way?*" Affirmation of a long-standing illness and any form of limitation classified the participant as having a limiting long-standing illness.

Doctor-diagnosed CHD, stroke, cancer, and chronic lung disease were self-reported in response to presentation of a list of conditions and the question: "*Has a doctor ever told you that you have (or have had) any of the conditions on this card*?"

Depressive symptoms were assessed with an eight-item version of the Center for Epidemiologic Studies Depression Scale (CES-D), a validated instrument for use in older adults (4). Respondents were asked to indicate whether they had experienced depressive symptoms (e.g. restless sleep and being unhappy) over the past month using a binary (yes/no) response. Total scores ranged from 0 to 8, with higher scores indicating more depressive symptoms. Data were dichotomised using an established cut-off, with a score of 4 or higher indicating clinically relevant symptomatology (5).

Method: calculation of Bayes factors

We used a conservative approach with alternative hypotheses represented by a half-normal distribution and expected effect sizes to RR=3 based on previous research that demonstrated a large multiplicative effect of smoking and BMI on circulatory disease mortality (6), and RR=2 and RR=1.5 to test for medium and small synergistic effects. BFs ≥3 can be interpreted as evidence for the alternative hypothesis (and against the null), BFs ≤1/3 as evidence for the null hypothesis, and BFs between 1/3 and 3 suggest the data are insensitive to distinguish the alternative hypothesis from the null (7).

For peer review only

For peer review only - http://bmjopen.bmj.com/site/about/guidelines.xhtml

Results: sensitivity analyses

Imputing outcomes at Wave 8 for participants who dropped out and did not report the presence of the outcome prior to dropout produced a very similar pattern of results, but the larger sample size meant 95% CIs were narrower and *p* values were smaller (Supplementary Table 2, Supplementary Figure 1). Multiplicative interactions between smoking and physical activity remained non-significant for all outcomes, with the exception of chronic lung disease which became statistically significant (p=0.044).

Restricting the sample to those with complete data at baseline (Wave 2) and final follow-up (Wave 8) also produced a similar pattern of results, although RRs were attenuated for the diagnosed diseases (CHD, stroke, cancer, chronic lung disease) (Supplementary Table 3, Supplementary Figure 2). The only notable change was that the RR for CHD in low active current smokers fell below that of low active former smokers, but this was likely caused by the reduced sample size leading to imprecise estimates for this group (*n*=121, 12 cases of incident CHD). Interactions between smoking and physical activity remained non-significant for all outcomes.

Excluding current smokers with lower levels of dependence (i.e. those who smoked <15 cigarettes per day, *n*=369) did not notably alter the results (Supplementary Table 4, Supplementary Figure 3), although as was observed when missing data were imputed, the interaction between smoking and physical activity for risk of developing chronic lung disease became statistically significant (p=0.033).

References

1. Steptoe A, Jackson SE. The Life Skills of Older Americans: Association with Economic, Psychological, Social, and Health Outcomes. Sci Rep. 2018 Jul 5;8(1):9669.

2. DeSalvo KB, Bloser N, Reynolds K, He J, Muntner P. Mortality prediction with a single general self-rated health question. A meta-analysis. J Gen Intern Med. 2006 Mar;21(3):267–75.

3. Jackson SE, Hackett RA, Steptoe A. Associations between age discrimination and health and wellbeing: cross-sectional and prospective analysis of the English Longitudinal Study of Ageing. Lancet Public Health. 2019;in press.

4. Radloff LS. The CES-D scale. Appl Psychol Meas. 1977;1(3):385–401.

5. Steffick DE. Documentation of affective functioning measures in the Health and Retirement Study. HRS Documentation Report DR-005; 2000.

For peer review only - http://bmjopen.bmj.com/site/about/guidelines.xhtml

6. Freedman DM, Sigurdson AJ, Rajaraman P, Doody MM, Linet MS, Ron E. The Mortality Risk of Smoking and Obesity Combined. Am J Prev Med. 2006 Nov;31(5):355–62.

7. Dienes Z. Using Bayes to get the most out of non-significant results. Front Psychol [Internet]. 2014 Jul 29 [cited 2018 Jul 4];5. Available from: https://www.ncbi.nlm.nih.gov/pmc/articles/PMC4114196/

For peer review only

For peer review only - http://bmjopen.bmj.com/site/about/guidelines.xhtml

Supplementary tables

Supplementary Table 1. Bayes factors for multiplicative interactions between smoking status and physical activity

	Large effects (RR=3)		Medium effects (RR=2)		Small effects (RR=1.5)	
	BF	Interpretation	BF	Interpretation	BF	Interpretation
Fair/poor self-rated health	0.23	Moderate evidence for H0	0.36	Data were insensitive	0.56	Data were insensitive
Limiting long-standing illness	0.28	Moderate evidence for H0	0.43	Data were insensitive	0.65	Data were insensitive
Coronary heart disease	0.48	Data were insensitive	0.68	Data were insensitive	0.88	Data were insensitive
Stroke	0.62	Data were insensitive	0.84	Data were insensitive	1.02	Data were insensitive
Cancer	0.30	Moderate evidence for H0	0.45	Data were insensitive	0.65	Data were insensitive
Chronic lung disease	0.04	Strong evidence for H0	0.06	Strong evidence for H0	0.11	Moderate evidence for H0
Clinically relevant depressive symptoms	0.29	Moderate evidence for H0	0.43	Data were insensitive	0.65	Data were insensitive

BF=Bayes factor, H0=null hypothesis, RR=relative risk.

For peer review only

Supplementary Table 2. Main effects of smoking status and physical activity and the interaction between smoking status and physical activity for risks of incident health problems over 12-year follow-up: imputed outcome data for participants who dropped out before Wave 8

	Smoking status			Physical activity		Interaction[1]	
	Never smoker	Former smoker	Current smoker	High active	Low active	Former smoker x low active	Current smoker x low active
Fair/poor self-rated health							
% (*n*)	48.9 (1168)	58.5 (1839)	69.7 (625)	48.9 (2071)	71.2 (1562)	-	-
RR [95% CI]	1	1.20 [1.09-1.32]	1.42 [1.25-1.63]	1	1.46 [1.33-1.60]	-	-
p		<0.001	<0.001		<0.001		
RR_{adj} [95% CI]	1	1.15 [1.02-1.30]	1.52 [1.27-1.81]	1	1.23 [1.05-1.44]	0.95 [0.78-1.15]	0.97 [0.74-1.27]
p		0.023	<0.001		0.012	0.567	0.810
Limiting long-standing illness							
% (*n*)	62.8 (1499)	69.8 (2192)	72.9 (654)	62.1 (2630)	78.2 (1715)	-	-
RR [95% CI]	1	1.11 [1.01-1.22]	1.16 [1.03-1.31]	1	1.26 [1.16-1.37]	-	-
p		0.025	0.019		<0.001		
RR_{adj} [95% CI]	1	1.08 [0.96-1.21]	1.18 [1.00-1.39]	1	1.11 [0.96-1.28]	0.98 [0.82-1.17]	1.06 [0.82-1.37]
p		0.195	0.053		0.166	0.786	0.666
Coronary heart disease							
% (*n*)	14.1 (335)	20.3 (639)	25.3 (227)	14.0 (594)	27.7 (607)	-	-
RR [95% CI]	1	1.45 [1.20-1.75]	1.79 [1.32-2.44]	1	1.97 [1.71-2.27]	-	-
p		<0.001	0.001		<0.001		
RR_{adj} [95% CI]	1	1.21 [0.96-1.53]	2.05 [1.43-2.94]	1	1.37 [1.02-1.82]	1.04 [0.71-1.54]	1.05 [0.69-1.61]
p		0.098	<0.001		0.034	0.829	0.810
Stroke							
% (*n*)	13.7 (327)	18.4 (579)	23.3 (209)	13.4 (567)	25.0 (547)	-	-
RR [95% CI]	1	1.34 [1.04-1.73]	1.70 [1.30-2.22]	1	1.86 [1.55-2.24]	-	-
p		0.027	0.001		<0.001		
RR_{adj} [95% CI]	1	1.20 [0.89-1.63]	2.03 [1.42-2.92]	1	1.42 [1.03-1.97]	0.87 [0.60-1.25]	1.01 [0.67-1.53]
p		0.216	<0.001		0.035	0.441	0.963

CI = confidence interval; RR = relative risk from bivariate models; RR_{adj} = relative risk adjusted for age, sex, ethnicity, wealth, alcohol intake, body mass index, and physical activity (for smoking status) or smoking status (for physical activity).

[1] Multiplicative interaction between smoking status and physical activity. Results can be interpreted as the difference in RR between high active and low active former/current smokers, relative to the difference in RR between high and low active never smokers. Thus, an interaction term above 1 indicates the disparity between high and low active groups was greater for former/current smokers than never smokers, and an interaction term below 1 indicates the disparity between high and low active groups was smaller for former/current smokers than never smokers.

Note: For each outcome, the sample is restricted to those who did not report the presence of the outcome at baseline. Results therefore indicate the prevalence and relative risk of new-onset health problems over the follow-up period.

For peer review only - http://bmjopen.bmj.com/site/about/guidelines.xhtml

Supplementary Table 2. (continued)

	Smoking status			Physical activity		Interaction[1]	
	Never smoker	Former smoker	Current smoker	High active	Low active	Former smoker x low active	Current smoker x low active
Cancer							
% (*n*)	18.0 (430)	21.9 (688)	24.6 (221)	17.7 (749)	26.9 (589)	-	-
RR [95% CI]	1	1.22 [1.06-1.40]	1.36 [1.07-1.73]	1	1.52 [1.21-1.90]	-	-
p		0.006	0.013		0.002		
RR_{adj} [95% CI]	1	1.13 [0.93-1.37]	1.57 [1.15-2.16]	1	1.34 [0.98-1.83]	0.96 [0.69-1.32]	0.95 [0.56-1.61]
p		0.207	0.006		0.065	0.792	0.831
Chronic lung disease							
% (*n*)	6.9 (164)	14.1 (443)	32.5 (292)	9.0 (382)	23.6 (517)	-	-
RR [95% CI]	1	2.05 [1.56-2.72]	4.76 [3.10-7.32]	1	2.61 [1.79-3.81]	-	-
p		<0.001	<0.001		0.001		
RR_{adj} [95% CI]	1	2.14 [1.42-3.24]	6.27 [3.88-10.09]	1	2.66 [1.65-4.27]	0.74 [0.49-1.12]	0.63 [0.40-0.99]
p		0.001	<0.001		<0.001	0.157	0.044
Clinically relevant depressive symptoms							
% (*n*)	52.8 (1259)	58.0 (1823)	62.9 (564)	52.4 (2219)	65.1 (1428)	-	-
RR [95% CI]	1	1.10 [0.98-1.24]	1.19 [1.02-1.39]	1	1.24 [1.13-1.36]	-	-
p		0.111	0.029		<0.001		
RR_{adj} [95% CI]	1	1.10 [0.96-1.26]	1.18 [0.98-1.43]	1	1.07 [0.91-1.27]	1.01 [0.84-1.22]	1.09 [0.82-1.43]
p		0.184	0.087		0.396	0.905	0.556

CI = confidence interval; RR = relative risk from bivariate models; RR_{adj} = relative risk adjusted for age, sex, ethnicity, wealth, alcohol intake, body mass index, and physical activity (for smoking status) or smoking status (for physical activity).

[1] Multiplicative interaction between smoking status and physical activity. Results can be interpreted as the difference in RR between high active and low active former/current smokers, relative to the difference in RR between high and low active never smokers. Thus, an interaction term above 1 indicates the disparity between high and low active groups was greater for former/current smokers than never smokers, and an interaction term below 1 indicates the disparity between high and low active groups was smaller for former/current smokers than never smokers.

Note: For each outcome, the sample is restricted to those who did not report the presence of the outcome at baseline. Results therefore indicate the prevalence and relative risk of new-onset health problems over the follow-up period.

For peer review only

Supplementary Table 3. Main effects of smoking status and physical activity and the interaction between smoking status and physical activity for risks of incident health problems over 12-year follow-up: sample restricted to participants with data at Wave 2 and Wave 8

	Smoking status			Physical activity		Interaction[1]	
	Never smoker	Former smoker	Current smoker	High active	Low active	Former smoker x low active	Current smoker x low active
Fair/poor self-rated health							
% (*n*)	31.5 (351)	39.0 (494)	45.4 (129)	32.9 (668)	47.9 (306)	-	-
RR [95% CI]	1	1.24 [1.06-1.45]	1.44 [1.14-1.84]	1	1.45 [1.24-1.71]	-	-
p		0.008	0.003		<0.001		
RR_{adj} [95% CI]	1	1.16 [0.96-1.41]	1.53 [1.13-2.05]	1	1.19 [0.90-1.56]	1.06 [0.74-1.52]	1.11 [0.65-1.91]
p		0.134	0.005		0.223	0.734	0.693
Limiting long-standing illness							
% (*n*)	47.5 (488)	52.6 (599)	50.0 (135)	48.2 (915)	57.1 (307)	-	-
RR [95% CI]	1	1.11 [0.96-1.28]	1.05 [0.83-1.33]	1	1.18 [1.01-1.39]	-	-
p		0.166	0.662		0.040		
RR_{adj} [95% CI]	1	1.09 [0.92-1.30]	1.08 [0.82-1.43]	1	1.07 [0.82-1.38]	0.98 [0.69-1.39]	1.18 [0.69-2.02]
p		0.321	0.572		0.626	0.926	0.543
Coronary heart disease							
% (*n*)	5.7 (73)	7.9 (114)	8.6 (32)	6.1 (138)	9.6 (81)	-	-
RR [95% CI]	1	1.39 [1.03-1.88]	1.52 [0.99-2.35]	1	1.56 [1.17-2.07]	-	-
p		0.033	0.056		0.002		
RR_{adj} [95% CI]	1	1.20 [0.82-1.76]	1.72 [0.99-2.98]	1	1.12 [0.66-1.88]	1.37 [0.72-2.63]	0.94 [0.38-2.35]
p		0.359	0.053		0.677	0.339	0.893
Stroke							
% (*n*)	5.4 (72)	6.5 (100)	8.1 (32)	5.6 (131)	7.9 (73)	-	-
RR [95% CI]	1	1.21 [0.89-1.65]	1.50 [0.98-2.31]	1	1.43 [1.06-1.92]	-	-
p		0.234	0.065		0.019		
RR_{adj} [95% CI]	1	1.02 [0.69-1.51]	1.42 [0.78-2.57]	1	1.07 [0.63-1.81]	1.15 [0.58-2.27]	1.59 [0.64-3.92]
p		0.918	0.247		0.812	0.682	0.315

CI = confidence interval; RR = relative risk from bivariate models; RR_{adj} = relative risk adjusted for age, sex, ethnicity, wealth, alcohol intake, body mass index, and physical activity (for smoking status) or smoking status (for physical activity).

[1] Multiplicative interaction between smoking status and physical activity. Results can be interpreted as the difference in RR between high active and low active former/current smokers, relative to the difference in RR between high and low active never smokers. Thus, an interaction term above 1 indicates the disparity between high and low active groups was greater for former/current smokers than never smokers, and an interaction term below 1 indicates the disparity between high and low active groups was smaller for former/current smokers than never smokers.

Note: For each outcome, the sample is restricted to those who did not report the presence of the outcome at baseline. Results therefore indicate the prevalence and relative risk of new-onset health problems over the follow-up period.

For peer review only

Supplementary Table 3. (continued)

	Smoking status			Physical activity		Interaction[1]	
	Never smoker	Former smoker	Current smoker	High active	Low active	Former smoker x low active	Current smoker x low active
Cancer							
% (*n*)	9.8 (125)	11.6 (173)	11.5 (44)	10.1 (228)	12.9 (114)	-	-
RR [95% CI]	1	1.19 [0.93-1.51]	1.17 [0.82-1.68]	1	1.28 [1.01-1.62]	-	-
p		0.162	0.388		0.044		
RR_{adj} [95% CI]	1	1.22 [0.91-1.64]	1.20 [0.74-1.94]	1	1.38 [0.93-2.06]	0.82 [0.49-1.39]	1.07 [0.51-2.25]
p		0.192	0.460		0.111	0.467	0.864
Chronic lung disease							
% (*n*)	2.6 (34)	5.0 (75)	14.4 (52)	3.4 (78)	9.4 (83)	-	-
RR [95% CI]	1	1.94 [1.28-2.93]	5.54 [3.54-8.66]	1	2.75 [2.00-3.78]	-	-
p		0.002	<0.001		<0.001		
RR_{adj} [95% CI]	1	2.67 [1.41-5.03]	7.02 [3.49-14.12]	1	4.09 [2.01-8.32]	0.47 [0.20-1.09]	0.45 [0.18-1.13]
p		0.002	<0.001		<0.001	0.077	0.088
Clinically relevant depressive symptoms							
% (*n*)	43.7 (283)	44.7 (334)	48.8 (104)	42.9 (480)	49.3 (241)	-	-
RR [95% CI]	1	1.02 [0.84-1.24]	1.12 [0.85-1.47]	1	1.15 [0.95-1.39]	-	-
p		0.832	0.430		0.147		
RR_{adj} [95% CI]	1	1.06 [0.84-1.33]	0.98 [0.68-1.41]	1	1.01 [0.74-1.38]	1.02 [0.67-1.55]	1.37 [0.77-2.41]
p		0.639	0.917		0.965	0.931	0.284

CI = confidence interval; RR = relative risk from bivariate models; RR_{adj} = relative risk adjusted for age, sex, ethnicity, wealth, alcohol intake, body mass index, and physical activity (for smoking status) or smoking status (for physical activity).

[1] Multiplicative interaction between smoking status and physical activity. Results can be interpreted as the difference in RR between high active and low active former/current smokers, relative to the difference in RR between high and low active never smokers. Thus, an interaction term above 1 indicates the disparity between high and low active groups was greater for former/current smokers than never smokers, and an interaction term below 1 indicates the disparity between high and low active groups was smaller for former/current smokers than never smokers.

Note: For each outcome, the sample is restricted to those who did not report the presence of the outcome at baseline. Results therefore indicate the prevalence and relative risk of new-onset health problems over the follow-up period.

For peer review only

Supplementary Table 4. Main effects of smoking status and physical activity and the interaction between smoking status and physical activity for risks of incident health problems over 12-year follow-up: excluding current smokers with low levels of nicotine dependence (<15 cigarettes/day)

	Smoking status			Physical activity		Interaction[1]	
	Never smoker	Former smoker	Current smoker	High active	Low active	Former smoker x low active	Current smoker x low active
Fair/poor self-rated health							
% (*n*)	40.9 (529)	49.0 (744)	63.8 (134)	42.2 (946)	59.3 (461)	-	-
RR [95% CI]	1	1.20 [1.05-1.37]	1.56 [1.23-1.98]	1	1.41 [1.22-1.61]	-	-
p		0.008	<0.001		<0.001		
RR_{adj} [95% CI]	1	1.14 [0.97-1.35]	1.75 [1.29-2.36]	1	1.19 [0.94-1.49]	0.99 [0.74-1.34]	0.99 [0.58-1.67]
p		0.115	<0.001		0.144	0.953	0.955
Limiting long-standing illness							
% (*n*)	57.1 (720)	62.7 (905)	70.8 (148)	58.2 (1282)	69.2 (491)	-	-
RR [95% CI]	1	1.10 [0.97-1.24]	1.24 [0.99-1.56]	1	1.19 [1.04-1.36]	-	-
p		0.143	0.067		0.012		
RR_{adj} [95% CI]	1	1.07 [0.93-1.24]	1.36 [1.03-1.79]	1	1.07 [0.86-1.32]	0.97 [0.73-1.29]	0.97 [0.58-1.63]
p		0.348	0.032		0.548	0.847	0.908
Coronary heart disease							
% (*n*)	8.8 (117)	11.7 (176)	14.9 (34)	9.0 (200)	14.9 (127)	-	-
RR [95% CI]	1	1.33 [1.04-1.70]	1.70 [1.13-2.55]	1	1.65 [1.30-2.09]	-	-
p		0.023	0.011		<0.001		
RR_{adj} [95% CI]	1	1.13 [0.82-1.55]	1.94 [1.10-3.41]	1	1.20 [0.79-1.83]	1.19 [0.70-2.02]	1.15 [0.49-2.69]
p		0.459	0.021		0.392	0.533	0.750
Stroke							
% (*n*)	8.2 (113)	9.9 (159)	12.6 (30)	7.9 (182)	13.1 (120)	-	-
RR [95% CI]	1	1.22 [0.95-1.56]	1.53 [1.00-2.35]	1	1.65 [1.29-2.10]	-	-
p		0.129	0.049		<0.001		
RR_{adj} [95% CI]	1	1.12 [0.80-1.57]	1.69 [0.87-3.26]	1	1.41 [0.93-2.13]	0.81 [0.47-1.39]	1.59 [0.65-3.89]
p		0.498	0.121		0.109	0.437	0.306

CI = confidence interval; RR = relative risk from bivariate models; RR_{adj} = relative risk adjusted for age, sex, ethnicity, wealth, alcohol intake, body mass index, and physical activity (for smoking status) or smoking status (for physical activity).

[1] Multiplicative interaction between smoking status and physical activity. Results can be interpreted as the difference in RR between high active and low active former/current smokers, relative to the difference in RR between high and low active never smokers. Thus, an interaction term above 1 indicates the disparity between high and low active groups was greater for former/current smokers than never smokers, and an interaction term below 1 indicates the disparity between high and low active groups was smaller for former/current smokers than never smokers.

Note: For each outcome, the sample is restricted to those who did not report the presence of the outcome at baseline. Results therefore indicate the prevalence and relative risk of new-onset health problems over the follow-up period.

For peer review only

Supplementary Table 4. (continued)

	Smoking status			Physical activity		Interaction[1]	
	Never smoker	Former smoker	Current smoker	High active	Low active	Former smoker x low active	Current smoker x low active
Cancer							
% (*n*)	13.4 (178)	15.8 (247)	19.8 (48)	13.8 (309)	18.4 (164)	-	-
RR [95% CI]	1	1.18 [0.96-1.45]	1.48 [1.05-2.10]	1	1.33 [1.08-1.63]	-	-
p		0.112	0.027		0.006		
RR_{adj} [95% CI]	1	1.11 [0.85-1.43]	1.85 [1.18-2.91]	1	1.29 [0.91-1.82]	1.01 [0.65-1.57]	0.81 [0.39-1.67]
p		0.446	0.008		0.149	0.974	0.565
Chronic lung disease							
% (*n*)	3.3 (44)	7.8 (120)	26.3 (61)	5.0 (111)	13.0 (114)	-	-
RR [95% CI]	1	2.34 [1.65-3.34]	7.89 [5.22-11.91]	1	2.60 [1.98-3.41]	-	-
p		<0.001	<0.001		<0.001		
RR_{adj} [95% CI]	1	2.80 [1.64-4.79]	11.40 [6.10-21.31]	1	3.49 [1.87-6.50]	0.57 [0.27-1.17]	0.40 [0.17-0.93]
p		<0.001	<0.001		<0.001	0.123	0.033
Clinically relevant depressive symptoms							
% (*n*)	53.5 (418)	56.4 (535)	63.0 (104)	52.4 (668)	62.5 (389)	-	-
RR [95% CI]	1	1.06 [0.90-1.24]	1.18 [0.90-1.55]	1	1.19 [1.02-1.40]	-	-
p		0.512	0.236		0.028		
RR_{adj} [95% CI]	1	1.10 [0.90-1.34]	1.18 [0.82-1.70]	1	1.09 [0.84-1.41]	0.95 [0.68-1.34]	1.11 [0.64-1.95]
p		0.370	0.375		0.527	0.783	0.705

CI = confidence interval; RR = relative risk from bivariate models; RR_{adj} = relative risk adjusted for age, sex, ethnicity, wealth, alcohol intake, body mass index, and physical activity (for smoking status) or smoking status (for physical activity).

[1] Multiplicative interaction between smoking status and physical activity. Results can be interpreted as the difference in RR between high active and low active former/current smokers, relative to the difference in RR between high and low active never smokers. Thus, an interaction term above 1 indicates the disparity between high and low active groups was greater for former/current smokers than never smokers, and an interaction term below 1 indicates the disparity between high and low active groups was smaller for former/current smokers than never smokers.

Note: For each outcome, the sample is restricted to those who did not report the presence of the outcome at baseline. Results therefore indicate the prevalence and relative risk of new-onset health problems over the follow-up period.

For peer review only

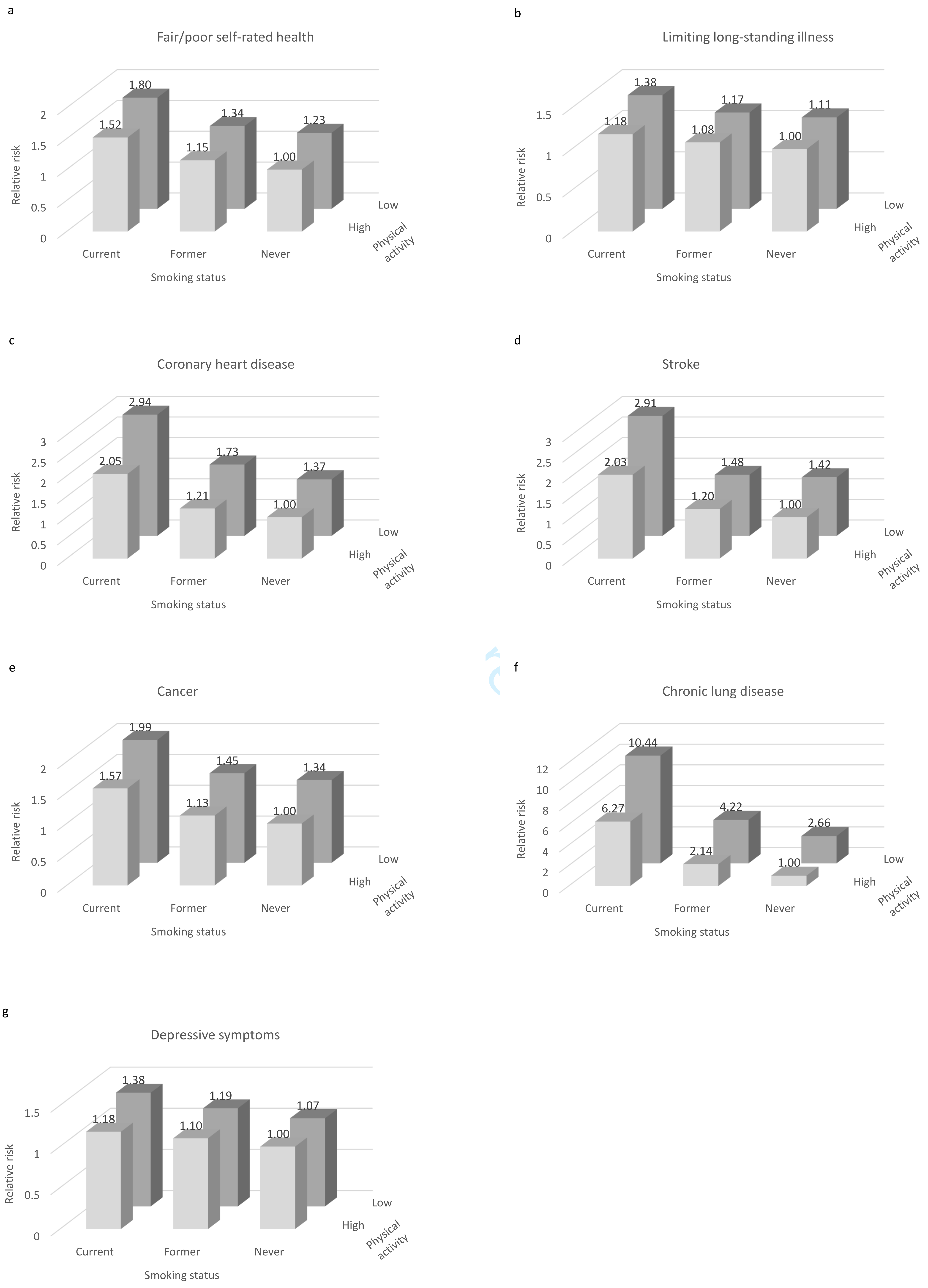
a
Fair/poor self-rated health
1.52
1.80
1.15
1.34
1.00
1.23
Relative risk
0
0.5
1
1.5
2
Current
Former
Never
Smoking status
Low
High
Physical activity
b
Limiting long-standing illness
1.18
1.38
1.08
1.17
1.00
1.11
Relative risk
0
0.5
1
1.5
Current
Former
Never
Smoking status
Low
High
Physical activity
c
Coronary heart disease
2.05
2.94
1.21
1.73
1.00
1.37
Relative risk
0
0.5
1
1.5
2
2.5
3
Current
Former
Never
Smoking status
Low
High
Physical activity
d
Stroke
2.03
2.91
1.20
1.48
1.00
1.42
Relative risk
0
0.5
1
1.5
2
2.5
3
Current
Former
Never
Smoking status
Low
High
Physical activity
e
Cancer
1.57
1.99
1.13
1.45
1.00
1.34
Relative risk
0
0.5
1
1.5
2
Current
Former
Never
Smoking status
Low
High
Physical activity
f
Chronic lung disease
6.27
10.44
2.14
4.22
1.00
2.66
Relative risk
0
2
4
6
8
10
12
Current
Former
Never
Smoking status
Low
High
Physical activity
g
Depressive symptoms
1.18
1.38
1.10
1.19
1.00
1.07
Relative risk
0
0.5
1
1.5
Current
Former
Never
Smoking status
Low
High
Physical activity

Supplementary Figure 1. Relative risks of developing (a) fair/poor self-rated health, (b) limiting long-standing illness, (c) coronary heart disease, (d) stroke, (e) cancer, (f) chronic lung disease, and (g) clinically relevant depressive symptoms over 12-year follow-up by baseline smoking/physical activity status, among older adults free of these conditions at baseline: imputed outcome data for participants who dropped out before Wave 8

For peer review only

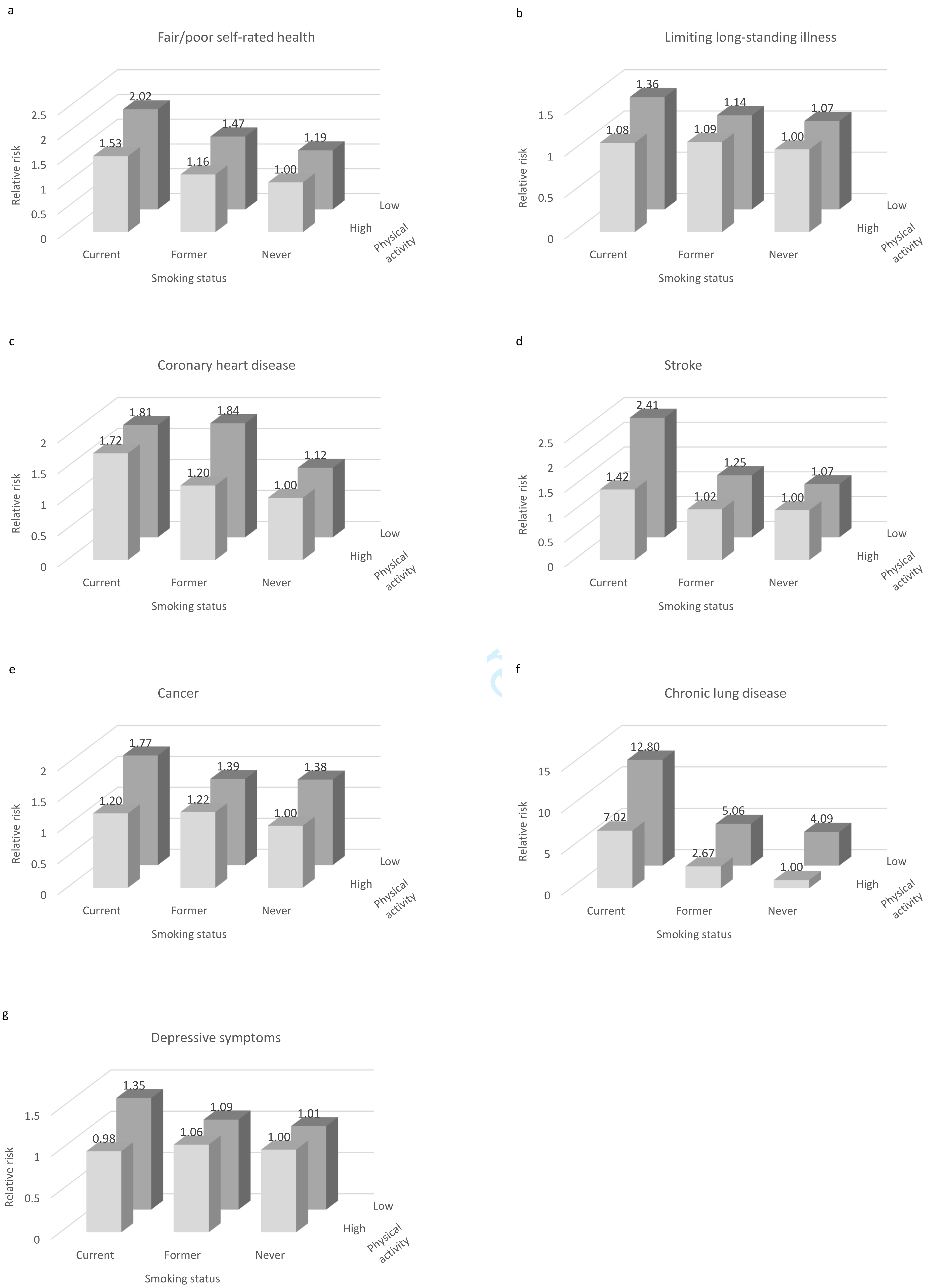
a
Fair/poor self-rated health
Relative risk
0
0.5
1
1.5
2
2.5
1.53
2.02
1.16
1.47
1.00
1.19
Current
Former
Never
Smoking status
Low
High
Physical activity
b
Limiting long-standing illness
Relative risk
0
0.5
1
1.5
1.08
1.36
1.09
1.14
1.00
1.07
Current
Former
Never
Smoking status
Low
High
Physical activity
c
Coronary heart disease
Relative risk
0
0.5
1
1.5
2
1.72
1.81
1.20
1.84
1.00
1.12
Current
Former
Never
Smoking status
Low
High
Physical activity
d
Stroke
Relative risk
0
0.5
1
1.5
2
2.5
1.42
2.41
1.02
1.25
1.00
1.07
Current
Former
Never
Smoking status
Low
High
Physical activity
e
Cancer
Relative risk
0
0.5
1
1.5
2
1.20
1.77
1.22
1.39
1.00
1.38
Current
Former
Never
Smoking status
Low
High
Physical activity
f
Chronic lung disease
Relative risk
0
5
10
15
7.02
12.80
2.67
5.06
1.00
4.09
Current
Former
Never
Smoking status
Low
High
Physical activity
g
Depressive symptoms
Relative risk
0
0.5
1
1.5
0.98
1.35
1.06
1.09
1.00
1.01
Current
Former
Never
Smoking status
Low
High
Physical activity

Supplementary Figure 2. Relative risks of developing (a) fair/poor self-rated health, (b) limiting long-standing illness, (c) coronary heart disease, (d) stroke, (e) cancer, (f) chronic lung disease, and (g) clinically relevant depressive symptoms over 12-year follow-up by baseline smoking/physical activity status, among older adults free of these conditions at baseline: sample restricted to participants with data at Wave 2 and Wave 8

For peer review only

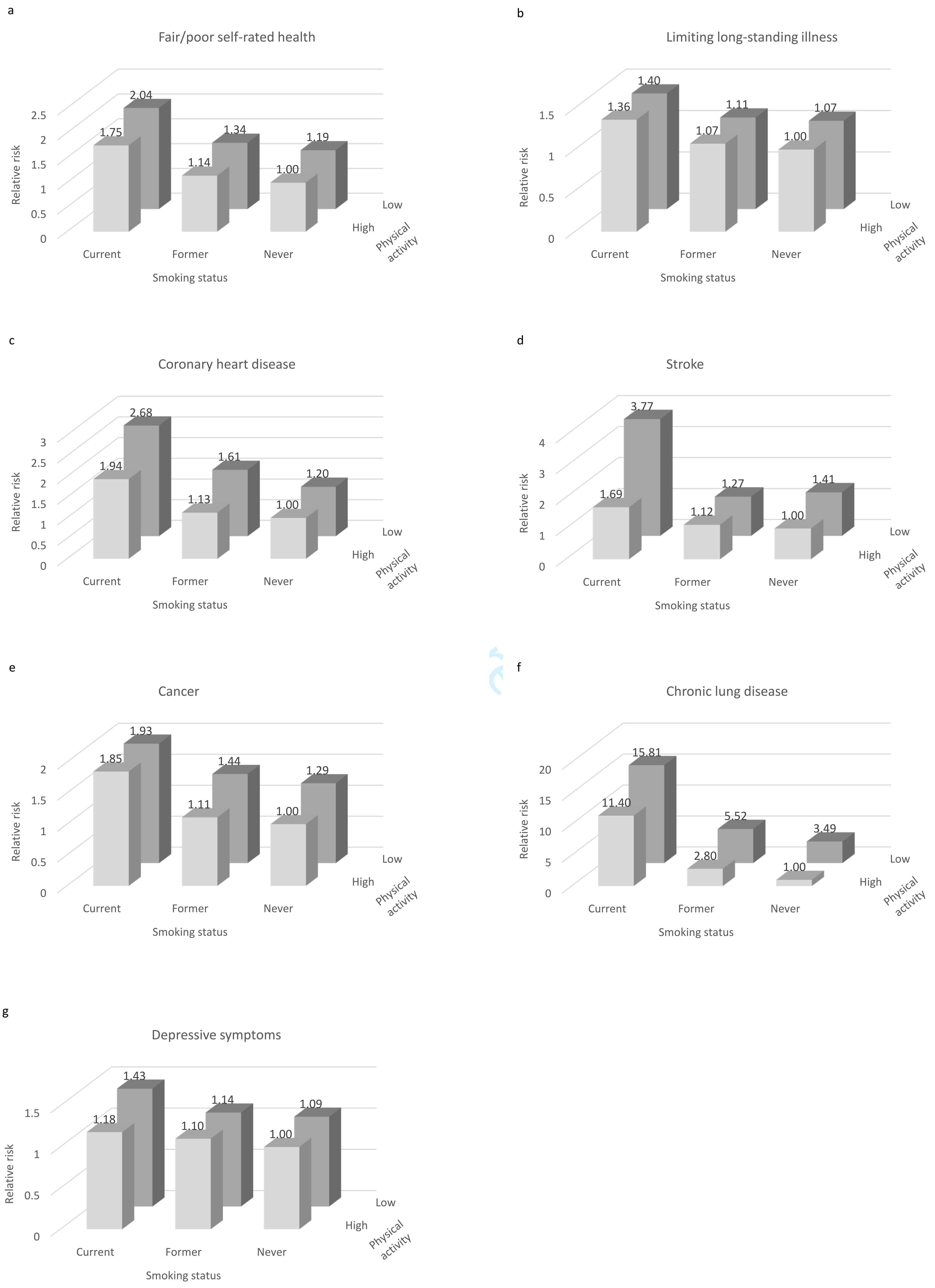
a
Fair/poor self-rated health
Relative risk
0
0.5
1
1.5
2
2.5
1.75
2.04
1.14
1.34
1.00
1.19
Current
Former
Never
Smoking status
Low
High
Physical activity
b
Limiting long-standing illness
Relative risk
0
0.5
1
1.5
1.36
1.40
1.07
1.11
1.00
1.07
Current
Former
Never
Smoking status
Low
High
Physical activity
c
Coronary heart disease
Relative risk
0
0.5
1
1.5
2
2.5
3
1.94
2.68
1.13
1.61
1.00
1.20
Current
Former
Never
Smoking status
Low
High
Physical activity
d
Stroke
Relative risk
0
1
2
3
4
1.69
3.77
1.12
1.27
1.00
1.41
Current
Former
Never
Smoking status
Low
High
Physical activity
e
Cancer
Relative risk
0
0.5
1
1.5
2
1.85
1.93
1.11
1.44
1.00
1.29
Current
Former
Never
Smoking status
Low
High
Physical activity
f
Chronic lung disease
Relative risk
0
5
10
15
20
11.40
15.81
2.80
5.52
1.00
3.49
Current
Former
Never
Smoking status
Low
High
Physical activity
g
Depressive symptoms
Relative risk
0
0.5
1
1.5
1.18
1.43
1.10
1.14
1.00
1.09
Current
Former
Never
Smoking status
Low
High
Physical activity

Supplementary Figure 3. Relative risks of developing (a) fair/poor self-rated health, (b) limiting long-standing illness, (c) coronary heart disease, (d) stroke, (e) cancer, (f) chronic lung disease, and (g) clinically relevant depressive symptoms over 12-year follow-up by baseline smoking/physical activity status, among older adults free of these conditions at baseline: excluding current smokers with low levels of nicotine dependence (<15 cigarettes/day)

For peer review only

STROBE Statement—Checklist of items that should be included in reports of ***cross-sectional studies***

	Item No	Recommendation	Page No
**Title and abstract**	1	(*a*) Indicate the study's design with a commonly used term in the title or the abstract	1
		(*b*) Provide in the abstract an informative and balanced summary of what was done and what was found	2
**Introduction**			
Background/rationale	2	Explain the scientific background and rationale for the investigation being reported	4-5
Objectives	3	State specific objectives, including any prespecified hypotheses	5
**Methods**			
Study design	4	Present key elements of study design early in the paper	6
Setting	5	Describe the setting, locations, and relevant dates, including periods of recruitment, exposure, follow-up, and data collection	6
Participants	6	(*a*) Give the eligibility criteria, and the sources and methods of selection of participants	6
Variables	7	Clearly define all outcomes, exposures, predictors, potential confounders, and effect modifiers. Give diagnostic criteria, if applicable	6-7
Data sources/ measurement	8*	For each variable of interest, give sources of data and details of methods of assessment (measurement). Describe comparability of assessment methods if there is more than one group	*6-7*
Bias	9	Describe any efforts to address potential sources of bias	7-8
Study size	10	Explain how the study size was arrived at	9
Quantitative variables	11	Explain how quantitative variables were handled in the analyses. If applicable, describe which groupings were chosen and why	6-8
Statistical methods	12	(*a*) Describe all statistical methods, including those used to control for confounding	7-8
		(*b*) Describe any methods used to examine subgroups and interactions	7-8
		(*c*) Explain how missing data were addressed	7-8
		(*d*) If applicable, describe analytical methods taking account of sampling strategy	Na
		(*e*) Describe any sensitivity analyses	8
**Results**			
Participants	13*	(a) Report numbers of individuals at each stage of study—eg numbers potentially eligible, examined for eligibility, confirmed eligible, included in the study, completing follow-up, and analysed	9
		(b) Give reasons for non-participation at each stage	Na
		(c) Consider use of a flow diagram	Na
Descriptive data	14*	(a) Give characteristics of study participants (eg demographic, clinical, social) and information on exposures and potential confounders	9
		(b) Indicate number of participants with missing data for each variable of interest	Tables
Outcome data	15*	Report numbers of outcome events or summary measures	Tables

For peer review only - http://bmjopen.bmj.com/site/about/guidelines.xhtml

Main results	16	(*a*) Give unadjusted estimates and, if applicable, confounder-adjusted estimates and their precision (eg, 95% confidence interval). Make clear which confounders were adjusted for and why they were included	Tables
		(*b*) Report category boundaries when continuous variables were categorized	Tables
		(*c*) If relevant, consider translating estimates of relative risk into absolute risk for a meaningful time period	Na
Other analyses	17	Report other analyses done—eg analyses of subgroups and interactions, and sensitivity analyses	10-11, tables
**Discussion**			
Key results	18	Summarise key results with reference to study objectives	11-12
Limitations	19	Discuss limitations of the study, taking into account sources of potential bias or imprecision. Discuss both direction and magnitude of any potential bias	13-14
Interpretation	20	Give a cautious overall interpretation of results considering objectives, limitations, multiplicity of analyses, results from similar studies, and other relevant evidence	12-13
Generalisability	21	Discuss the generalisability (external validity) of the study results	13-14
**Other information**			
Funding	22	Give the source of funding and the role of the funders for the present study and, if applicable, for the original study on which the present article is based	16

*Give information separately for exposed and unexposed groups.

Note: An Explanation and Elaboration article discusses each checklist item and gives methodological background and published examples of transparent reporting. The STROBE checklist is best used in conjunction with this article (freely available on the Web sites of PLoS Medicine at http://www.plosmedicine.org/, Annals of Internal Medicine at http://www.annals.org/, and Epidemiology at http://www.epidem.com/). Information on the STROBE Initiative is available at www.strobe-statement.org.

For peer review only

For peer review only - http://bmjopen.bmj.com/site/about/guidelines.xhtml

BMJ Open

The combined health risks of cigarette smoking and low levels of physical activity: a prospective cohort study in England with 12-year follow-up

Journal:	*BMJ Open*
Manuscript ID	bmjopen-2019-032852.R1
Article Type:	Original research
Date Submitted by the Author:	14-Oct-2019
Complete List of Authors:	Jackson, Sarah ; University College London, Epidemiology and Public Health;<br>Brown, Jamie; University College London, Psychology & Language Sciences<br>Ussher, Michael; St George's University of London, Division of Population Health Sciences and Education<br>Shahab, Lion; UCL, Dept of Epidemiology and Public Health<br>Steptoe, Andrew; University College London, Epidemiology and Public Health<br>Smith, Lee; Anglia Ruskin University - Cambridge Campus, Department of Life Sciences
**Primary Subject Heading**:	Epidemiology
Secondary Subject Heading:	Public health
Keywords:	smoking, physical activity, cancer, cardiovascular disease, lung disease, depresison

SCHOLARONE™
Manuscripts

The combined health risks of cigarette smoking and low levels of physical activity: a prospective cohort study in England with 12-year follow-up

Sarah E. Jackson[1], Jamie Brown[1,2], Michael Ussher[3,4], Lion Shahab[1], Andrew Steptoe[1], Lee Smith[5]

[1] Department of Behavioural Science and Health, University College London, London, UK

[2] Department of Clinical, Educational and Health Psychology, University College London, UK

[3] Population Health Research Institute, St George's, University of London, London, UK

[4] Institute of Social Marketing and Health, University of Stirling, Stirling, UK

[5] The Cambridge Centre for Sport and Exercise Sciences, Anglia Ruskin University, UK

Corresponding author: Dr Sarah Jackson, Department of Behavioural Science and Health, University College London, 1-19 Torrington Place, London WC1E 7HB, UK. s.e.jackson@ucl.ac.uk +44 (0)207 679 8312

For peer review only

For peer review only - http://bmjopen.bmj.com/site/about/guidelines.xhtml

Abstract

Objectives: To (i) estimate the combined risks of cigarette smoking and physical inactivity for chronic disease, disability, and depressive symptoms, and (ii) determine whether risks associated with these behaviours are additive or synergistic.

Design and setting: Longitudinal observational population study using data from Waves 2 (2004/05) through 8 (2016/17) of the English Longitudinal Study of Ageing, a prospective study of community-dwelling older adults in England.

Participants: 6,425 men and women aged ≥52 years (mean [SD] 65.88 [9.34] years) at baseline.

Main outcome measures: Smoking status (never; former; current) and level of physical activity (high, defined as moderate/vigorous physical activity [MVPA] more than once a week; low, defined as MVPA once a week or less) were self-reported at Wave 2 baseline. Self-rated health, limiting long-standing illness, chronic conditions (coronary heart disease [CHD], stroke, cancer, chronic lung disease), and depressive symptoms were reported in each biennial wave.

Results: Both smoking and low levels of physical activity were associated with increased risk of incident health problems over the 12-year follow-up period. Current smokers with low levels of physical activity had especially high risks of developing fair/poor self-rated health, CHD, stroke, cancer, and chronic lung disease compared with highly active never smokers (RR_{adj} range 1.89-14.00). While additive effects were evident, tests of multiplicative interactions revealed no evidence of large synergistic effects of smoking and low physical activity (Bayes factor range 0.04-0.61), although data were insensitive to detect smaller effects.

Conclusions: Among older adults in England, there was no evidence of large synergistic effects of smoking and low levels of physical activity on risk of developing chronic disease or depressive symptoms over 12 years. However, additive effects of smoking and low levels of physical activity were evident, underscoring the importance of each of these lifestyle risk behaviours for disease onset.

Key words: smoking; physical activity; self-rated health; coronary heart disease; stroke; cancer; lung disease; depression

For peer review only - http://bmjopen.bmj.com/site/about/guidelines.xhtml

Strengths and limitations of this study

- This study represents the first effort comprehensively to examine the combined risks of smoking and low levels of physical activity in a large prospective cohort study.
- Adjustment for a range of relevant covariates took into account potential confounders of the associations between exposures and outcomes.
- Findings were robust to three sensitivity analyses taking different analytic approaches.
- Reliance on self-reported data introduced potential for bias.
- We did not model dynamic effects (i.e. the impact of changes in smoking status and physical activity across the time period on disease outcomes) which may have masked some associations.

For peer review only

For peer review only - http://bmjopen.bmj.com/site/about/guidelines.xhtml

Introduction

Smoking and low levels of physical activity are among the leading causes of preventable morbidity and mortality worldwide (1). Each is associated with substantially increased risk of developing a host of chronic diseases, including coronary heart disease (CHD), cancer, and chronic lung diseases (2–5). Quitting smoking and taking up physical activity leads to improvements in overall health and longevity, even relatively late in life (2,6–8). Associations between low levels of physical activity and poorer mental health outcomes, including depression and anxiety disorders, have consistently been reported (9,10) and physical inactivity (defined as not meeting the recommended physical activity guidelines for good health) appears to be causally related to mental health conditions (3). The evidence on smoking is mixed, with some studies suggesting that the association with poor mental health can largely be explained by common causes, such as genes that predispose to both smoking and depression (11,12) and others finding evidence for a causal relationship (13,14).

The combined health risks associated with smoking and physical inactivity have not been comprehensively examined. This is important because health risk behaviours tend to cluster within individuals (15–17). Studies in large, representative samples have shown the majority of adults in England and the US have multiple lifestyle risk factors (e.g. smoking, physical inactivity, excessive alcohol intake, low fruit and vegetable consumption) (15,17), and there is evidence to suggest that combinations of lifestyle risk factors have a greater adverse impact on health than would be expected from the added individual effects alone (18–22). If lifestyle risk factors work synergistically (i.e. greater than the sum of the risks associated with each behaviour individually, indicating the behaviours act as effect modifiers for each other) rather than additively (i.e. the combined risk is greater than the individual risks associated with each behaviour) to influence disease risk, there may be potential to increase the public health impact of behaviour change interventions by targeting multiple behaviours (23–25). However, the extant literature on the benefits of multiple behaviour change interventions is mixed, and their effectiveness likely depends upon particular behaviour combinations and whether there is genuine synergy between them (24). A Cochrane review of randomised controlled trials (RCTs) of physical activity in addition to smoking cessation treatment found mixed results, with the majority failing to provide evidence that physical activity aids smoking cessation (26). However, most of these trials had small samples or a physical activity component insufficiently intense to achieve the desired level of activity (26). Examination of the risks associated with smoking and physical activity in combination is important in order to determine synergistic health effects of these risk factors and

For peer review only - http://bmjopen.bmj.com/site/about/guidelines.xhtml

evaluate the potential usefulness of further research targeting this combination of behaviours in interventions for primary prevention.

There is some evidence from cross-sectional studies to suggest smoking and physical activity interact to influence the risk of adverse physical and mental health outcomes. For example, in a large sample of adult smokers, physical activity was found to moderate the association between nicotine dependence and depression (27). The results indicated smokers with high nicotine dependence and low physical activity were more likely to be depressed than would be expected on the basis of individual effects of smoking and physical inactivity. Similarly, a survey of undergraduate smokers found that those with a lower level of physical activity had higher odds of depression (28). However, the cross-sectional study design makes it very difficult to interpret the direction of associations. For example, it is possible being depressed leads to the uptake of smoking and a loss of interest in physical activity, as opposed to being the result of these behaviours. A number of RCTs have examined the impact of physical activity on smoking and cessation outcomes, and provided strong evidence exercise reduces nicotine cravings and withdrawal symptoms (26,29,30), although a positive impact on relapse has not clearly been demonstrated (31). Regarding physical health effects, the evidence is mixed. For example, two small experimental studies have examined the impact of physical activity on cardiovascular biomarkers in smokers, and observed improvements in the cardiovascular risk profile over three months (32,33). A cohort study of adults in Copenhagen followed for an average of 11 years found that smokers who engaged in moderate to high levels of regular physical activity experienced a smaller decline in lung function decline and lower COPD risk than those who were less active (34). However, another study that followed middle-aged men in Japan over a similar duration observed no significant interaction between smoking status and level of physical activity for risk of pancreatic cancer (35). To the best of our knowledge, no studies have evaluated synergistic effects of smoking and physical activity on depression or chronic disease in a large, representative sample using a prospective design.

Using data collected over 12 years from a large population-based sample of older adults living in England, this study therefore aimed to examine the risks of chronic disease and poor mental health associated with cigarette smoking and low levels of physical activity combined. Specifically, we aimed to answer the following research questions:

1. To what extent is the combination of smoking and low physical activity associated with increased risk of the incidence of poor self-rated health, limiting long-standing illness, CHD, stroke, cancer,

For peer review only - http://bmjopen.bmj.com/site/about/guidelines.xhtml

chronic lung disease, and depressive symptoms over 12-year follow-up among older adults who are healthy at baseline, over and above the risks associated with smoking or low physical activity alone, or neither smoking nor low physical activity?

2. Are the combined risks of smoking and low physical activity for these outcomes additive or synergistic?

Method

Design

This investigation used data from the English Longitudinal Study of Ageing (ELSA) covering a 12-year period. ELSA is a population-based longitudinal panel study of a representative sample of men and women aged 50 and older living in England. The study began in 2002 (Wave 1), with participants recruited from an annual cross-sectional survey of households. Data are collected every two years via computer-assisted personal interview and self-completion questionnaires. In alternate (even) waves there is an additional health examination, in which objective measures are obtained. For the present study, baseline data were drawn from Wave 2 (2004/05; the first wave in which height and weight were measured, allowing inclusion of body mass index (BMI) in the analyses), collected when participants were aged ≥52 years. Follow-up data were collected biennially through to Wave 8 (2016/17; the most recent wave of available data).

Measures

Measurement of exposures

Smoking status was defined as current, former, or never smoker on the basis of responses to two yes/no questions: 1) "*Have you ever smoked cigarettes?*" 2) "*Do you smoke cigarettes at all nowadays?*" This measure has been validated against salivary cotinine levels in the Health Survey for England (36).

Physical activity was assessed with three items that asked participants how often they took part in activities that were vigorous (e.g. jogging, cycling), moderately energetic (e.g. gardening, walking at moderate pace), or mildly energetic (e.g. laundry, home repairs). Response options were: more than once a week, once a week, 1-3 times a month, hardly ever/never. Activity examples provided to respondents correspond to metabolic equivalent of task ≥6, ≥3.5 to <6, and ≥2 to <3.5 respectively for vigorous, moderate, and mild

For peer review only - http://bmjopen.bmj.com/site/about/guidelines.xhtml

activities. For the purpose of analysis, we categorised physical activity into two categories: high physical activity (moderate and/or vigorous activity more than once a week) vs. low physical activity (moderate/vigorous activity once a week or less). This measure has been validated against objective, accelerometer-measured hours of moderate-vigorous intensity activity and demonstrates convergent validity in grading a wide range of psychosocial, physical, and biochemical outcomes (7,37–41).

Measurement of outcomes

We included as outcomes two measures of subjective health (self-rated health, limiting long-standing illness), four diagnosed chronic conditions (CHD, stroke, cancer, chronic lung disease), and one measure of mental health (clinically relevant depressive symptoms).

Self-rated health was assessed using a single item: *"Would you say your health is… poor/fair/good/very good/excellent?"* We analysed the proportion of individuals rating their health as fair/poor, as has been done in previous investigations (42–44).

We also used data on self-reported limiting long-standing illness, which reflects the extent to which participants feel their daily activities are limited by the presence of illness. This was assessed with two questions: 1) "*Do you have any long-standing illness, disability, or infirmity? By long-standing I mean anything that has troubled you over a period of time or that is likely to affect you over a period of time*." Those who respond yes were asked: 2) "*Does this illness or disability limit your activities in any way?*" Affirmation of a long-standing illness and any form of limitation classified the participant as having a limiting long-standing illness.

Doctor-diagnosed CHD, stroke, cancer, and chronic lung disease were self-reported in response to presentation of a list of conditions and the question: "*Has a doctor ever told you that you have (or have had) any of the conditions on this card*?"

Depressive symptoms were assessed with an eight-item version of the Center for Epidemiologic Studies Depression Scale (CES-D), a validated instrument for use in older adults (45). Respondents were asked to indicate whether they had experienced depressive symptoms (e.g. restless sleep and being unhappy) over the past month using a binary (yes/no) response. Total scores ranged from 0 to 8, with higher scores indicating more depressive symptoms. Data were dichotomised using an established cut-off, with a score of 4 or higher indicating clinically relevant symptomatology (46).

For peer review only - http://bmjopen.bmj.com/site/about/guidelines.xhtml

For each outcome of interest, we analysed the proportion of participants free from that outcome at Wave 2 baseline who reported the presence of the outcome in Wave 3, 4, 5, 6, 7 or 8 (coded 1). Therefore, our dependent variables incorporated all new-onset adverse health outcomes reported by participants across the 12-year follow-up period. For our primary analyses, participants retained in the study at Wave 8 who did not report the presence of the outcome in any wave were coded 0. Participants lost to follow-up before Wave 8 who did not report the presence of the outcome in any wave were coded as missing, because it was not possible to determine their status.

Measurement of covariates

Demographic variables included baseline age, sex, and ethnicity (white vs. non-white). Sociodemographic position was indexed using household non-pension wealth, which has been identified as a particularly sensitive indicator in this population (47). Past-year alcohol intake was categorised as never/rare (never – once or twice a year), regular but infrequent (once every couple of months – twice a week), or frequent (3 days a week – almost every day). BMI was calculated as weight in kilograms/(height in metres)^2 based on objective measurements.

Statistical analysis

The analysis plan was pre-registered on Open Science Framework (https://osf.io/g9p2b/). We amended our pre-specified definition of physical activity categories upon seeing the distribution of the data, because our original dichotomy of moderate/vigorous physical activity at least once a week resulted in an implausibly high proportion of the sample being classified as high active (~80%). For transparency, results based on the original categorisation are available on Open Science Framework.

We used one-way independent analyses of variance (ANOVA; continuous variables) and Pearson's chi-square tests (categorical variables) to analyse differences in baseline characteristics by smoking status (never/former/current) and level of physical activity (high/low).

We used log-binomial regression to calculate the relative risks (RR) with 95% confidence intervals (CIs) associated with smoking and physical activity of incident fair/poor self-rated health, limiting long-standing illness, CHD, stroke, cancer, chronic lung disease, and depressive symptoms over 12-year follow-up among participants who did not report the outcome of interest at baseline. We constructed five models for each outcome. The first and second calculated unadjusted RRs associated with smoking status (reference

For peer review only - http://bmjopen.bmj.com/site/about/guidelines.xhtml

category: never smoker) and physical activity (reference category: high active), respectively. The third tested main effects of smoking status and physical activity, and the multiplicative interaction between smoking status and physical activity, controlling for covariates. The fourth and fifth calculated unadjusted and adjusted RRs, respectively, associated with each combination of smoking status and level of physical activity: (i) never smoker/high active (reference category); (ii) never smoker/low active; (iii) former smoker/high active; (iv) former smoker/low active; (v) current smoker/high active; and (vi) current smoker/low active.

We performed three sensitivity analyses. The first imputed missing outcomes data for those who dropped out of ELSA before Wave 8 and did not report the presence of any of these conditions in their completed waves. A multiple imputation model was run with all exposures and covariates entered as predictors. Five imputed datasets were created, each was analysed separately, and the results were combined to produce pooled estimates of effects. The second sensitivity analysis restricted the sample to those with complete data at Wave 2 and Wave 8 to assess healthy survivor effects. The third excluded current smokers who smoke <15 cigarettes per day (indicative of a lower level of nicotine dependence) in order to address the potential issue of differential rates of smoking cessation in relation to level of physical activity (48). One would expect a lower rate of successful quitting during the follow-up period among more dependent smokers, so it was thought that excluding those who were less dependent may provide a better reflection of the combined health risks of smoking and low physical activity rather than an artefact of more successful quitting among active smokers generally.

In order to evaluate the extent to which our data supported the null hypothesis (i.e. no synergistic relationship between smoking and physical activity for risk of incident health problems), the experimental hypothesis (i.e. synergy between smoking and physical activity), or were insensitive, we calculated Bayes factors (BFs) for the multiplicative interaction results (see Supplementary Material for details).

All analyses were conducted in SPSS v.24, with the exception of the BFs which were calculated using an online calculator (http://www.lifesci.sussex.ac.uk/home/Zoltan_Dienes/inference/Bayes.htm).

Public and patient involvement

No patients were involved in setting the research questions or outcome measures, nor were they involved in the design and implementation of the study. There are no plans to involve patients in dissemination.

For peer review only - http://bmjopen.bmj.com/site/about/guidelines.xhtml

Results

Sample characteristics

There were 9,432 individuals in Wave 2 of ELSA, of whom 7,666 (81.3%) participated in the health examination in which objective measurements of height and weight were obtained. We excluded 1,241 individuals (16.2%) with missing data, leaving a final sample for analysis of 6,425 participants. Compared with those who were excluded, the analysed sample had a similar mean age but were more likely to be male, white, and wealthier. They were also more likely to drink alcohol regularly or frequently and had a higher mean BMI, but were less likely to smoke or have low physical activity. The prevalence of chronic disease and depressive symptoms was generally lower in the analysed sample (Supplementary Table 1).

Table 1 presents descriptive characteristics measured at Wave 2 baseline overall and by smoking status and level of physical activity. The sample comprised 2,902 men and 3,523 women aged ≥52 years (mean [SD] 65.88 [9.34] years). Participants were predominantly white (98.8%) and the upper quintiles of wealth were overrepresented. The majority (81.1%) reported regular or frequent alcohol intake and the mean BMI was in the overweight range (27.91 [4.87] kg/m^2). The prevalence of chronic disease and depressive symptoms ranged from 2.4% (stroke) to 32.9% (limiting long-standing illness).

Some 14.0% of participants were current smokers, 48.9% were former smokers, and 37.2% were never smokers. Those who reported current smoking tended to be younger than never/former smokers, and more were from the lower quintiles of wealth. Current and former smokers were more likely that never smokers to be female and white. Former smokers were the most likely to report drinking alcohol frequently and had the highest BMI. Current smokers were the most likely to have low levels of physical activity. They were also more likely than former and never smokers to rate their health as fair or poor, and to report the presence of limiting long-standing illness, diagnosed chronic lung disease, and clinically relevant depressive symptoms. Former smokers were the most likely to report CHD and stroke.

Just over a third (34.1%) were classified as having low physical activity. Relative to those with high levels of physical activity, participants with low levels of physical activity were older on average, and a higher proportion were female and from the lower quintiles of wealth (Table 1). They were less likely to drink alcohol frequently, had a higher mean BMI, and were more likely to be current smokers. Participants with

For peer review only - http://bmjopen.bmj.com/site/about/guidelines.xhtml

low levels of physical activity were also more likely than those with high levels of physical activity to rate their health as fair or poor, and to report the presence of a limiting long-standing illness, diagnosed CHD, stroke, cancer, or chronic lung disease, and clinically relevant depressive symptoms.

Associations with incident health problems

For each outcome, Table 2 summarises the absolute risk and unadjusted and adjusted RRs associated with smoking status and physical activity, and interactions between smoking status and physical activity. Table 3 shows the absolute risk and unadjusted and adjusted RRs associated with each combination of smoking status and physical activity.

Main effects of smoking status

In unadjusted models (Table 2), both former and current smokers had significantly higher risks of developing fair/poor self-rated health, CHD, and chronic lung disease than never smokers (RR range 1.20-2.34 for former smokers, RR range 1.45-6.28 for current smokers). Risk of stroke was significantly higher among current smokers than never smokers (RR 1.58), but did not differ significantly between former and never smokers (RR 1.22). Smoking status was not significantly associated with risk of developing a limiting long-standing illness, cancer, or clinically relevant depressive symptoms (RR range 1.10-1.28).

After adjustment for age, sex, ethnicity, wealth, alcohol intake, BMI, and level of physical activity (Table 2), the risk of developing chronic lung disease remained significantly higher among former (RR_{adj} 2.77) and current smokers (RR_{adj} 8.33), and risks of developing fair/poor self-rated health, CHD, and stroke were significantly higher among current smokers (RR_{adj} range 1.55-1.93), relative to never smokers. The risk of developing cancer approached statistical significance for current versus never smokers (RR_{adj} 1.44).

Main effects of physical activity

In unadjusted models (Table 2), participants with low physical activity had significantly higher risks of developing fair/poor self-rated health, limiting long-standing illness, CHD, stroke, cancer, chronic lung disease, and clinically relevant depressive symptoms than those with high physical activity (RR range 1.19-2.67).

After adjustment for age, sex, ethnicity, wealth, alcohol intake, BMI, and smoking status (Table 2), the risk of developing chronic lung disease remained significantly higher among those with low versus high physical

For peer review only - http://bmjopen.bmj.com/site/about/guidelines.xhtml

activity (RR_{adj} 3.50), but other associations were attenuated and became non-significant (RR_{adj} range 1.06-1.40).

Additive and synergistic effects of smoking status and physical activity

After adjustment for covariates, significant differences in risks of developing fair/poor self-rated health, CHD, stroke, cancer, and chronic lung disease were observed across different combinations of smoking status and levels of physical activity (Table 3, Figure 1).

Relative to never smokers with high physical activity, current smokers with low physical activity had the highest risks of each of these outcomes (RR_{adj} range 1.89-14.00). Risks of fair/poor self-rated health, CHD, stroke, and chronic lung disease were also significantly elevated among current smokers with high physical activity (RR_{adj} range 1.55-8.33), and the risk of cancer approached significance (RR_{adj} 1.44), although relative risks were lower than those for current smokers with low physical activity.

Risks of fair/poor self-rated health, CHD, cancer, and chronic lung disease were also significantly elevated for those with low physical activity who had stopped smoking, although risks relative to never smokers with high physical activity were comparatively lower than were observed for current smokers (RR_{adj} range 1.35-5.42). Chronic lung disease was the only outcome for which significantly elevated risk was observed among former smokers with high physical activity (RR_{adj} 2.77) or never smokers with low physical activity (RR_{adj} 3.50), relative to never smokers with high physical activity.

The risks of limiting long-standing illness and clinically relevant depressive symptoms did not differ significantly across smoking/physical activity groups, although point estimates followed a similar pattern (Table 3, Figure 1).

While additive effects were evident, with the health risks associated with the combination of current smoking and low physical activity higher than those associated with one or other of these behaviours in isolation (Table 3, Figure 1), tests of multiplicative interactions revealed no evidence of synergistic effects of smoking and low physical activity (Table 2). The only outcome for which the interaction approached statistical significance was chronic lung disease (p=0.070), where the effect was in the opposite direction to what we hypothesised, i.e. relative to never smokers, the increase in risk associated with inactivity appears smaller in current smokers.

For peer review only - http://bmjopen.bmj.com/site/about/guidelines.xhtml

BFs based on large synergistic effects between smoking status and physical activity indicated the data provided strong evidence for the null hypothesis for chronic lung disease and moderate evidence for the null hypothesis for incident fair/poor self-rated health, limiting long-standing illness, cancer, and depressive symptoms, but were insensitive to detect large effects for CHD and stroke (Supplementary Table 2). BFs based on medium and small synergistic effects favoured the null hypothesis but indicated the data were insensitive for all outcomes except chronic lung disease.

Sensitivity analyses

Sensitivity analyses taking three different analytic approaches produced a very similar pattern of results (see Supplementary Material, Supplementary Tables 3-5 and Supplementary Figures 1-3 for full details).

Discussion

In this large prospective study of older adults, we examined the risks of incident self-rated health, limiting long-standing illness, CHD, stroke, cancer, chronic lung disease, and depressive symptoms over 12-year follow-up associated with smoking and low levels of physical activity among individuals free of these conditions at baseline. We observed additive effects of smoking and low physical activity on these outcomes, with older adults who reported both current smoking and low physical activity at higher risk of developing these conditions than those who engaged in one or neither of these lifestyle risk behaviours. However, there was no evidence of synergistic effects of smoking and low physical activity on the incidence of these conditions.

It has been proposed that targeting multiple behaviours could increase the public health impact of behaviour change interventions (23–25), but evidence on the effectiveness of this strategy is inconsistent (24). For example, studies focusing on physical activity and diet have shown interventions that focus on a single behaviour are more effective in increasing the target behaviours, while those that target both behaviours result in greater weight loss (49). Dieting while trying to stop smoking is associated with worse smoking outcomes (50), and it is generally recommended smokers do not attempt to diet until several months after quitting (50). It is likely that the effectiveness of multiple behaviour change interventions relies on there being a synergistic relationship between the target behaviours. The failure of the present study to find evidence of synergy between smoking and low physical activity on risk of chronic disease and

For peer review only - http://bmjopen.bmj.com/site/about/guidelines.xhtml

For peer review only

depressive symptoms suggests targeting this combination of behaviours is unlikely to be more effective in reducing the risk of these adverse health outcomes than focusing on each behaviour separately. This is consistent with findings of RCTs that have examined effects of physical activity as an adjunct to smoking cessation treatment. A 2014 Cochrane review (26) identified 20 RCTs (total *n*=5,870) that compared an exercise-only intervention or a combined exercise and smoking cessation intervention with a cessation only intervention. Just two of the 20 trials found a beneficial effect of including an exercise component on long-term cessation (26).

However, despite the lack of evidence for synergy between these behaviours, there are other reasons why targeting smoking and physical activity in a multiple behaviour change intervention may be beneficial. For example, changes in physical activity as a result of an intervention may interact differently with smoking compared with more spontaneous changes in physical activity (as reported in cohort studies) and especially so if the intervention is actively used to promote cessation (e.g. as a means for reducing cigarette cravings (30)). It is also possible that smoking and physical activity may interact in different ways depending on the timing of changes in the two behaviours (51).

While the present results provide no evidence for synergistic effects of smoking and low physical activity on health, there were clear additive effects. Current smokers were at higher risk of incident health problems than former or never smokers. People with low physical activity were at higher risk of incident health problems than those who engaged in regular moderate/vigorous intensity physical activity. The combination of current smoking and low physical activity conferred the highest risk of each outcome: notably, individuals who reported both behaviours had more than twice the risk of developing CHD, three times higher risk of having a stroke, and 14 times higher risk of developing chronic lung disease over 12-year follow-up than never smokers who engaged in regular physical activity. These results emphasise the importance of promoting both abstinence from smoking and regular physical activity, and intervening to encourage behaviour change for people with unhealthy lifestyles.

This study had several strengths. The sample was drawn from a large, nationally-representative cohort of older adults. The prospective design facilitated assessment of the temporal relationship between smoking and physical inactivity and future disease onset. Assuming the health risk behaviours have a cumulative (dose-response) effect on health outcomes, the older age of the sample meant we had a better chance of detecting an effect given longer exposure in this population group. Adjustment for a range of relevant

For peer review only - http://bmjopen.bmj.com/site/about/guidelines.xhtml

covariates took into account potential confounders of the associations between exposures and outcomes. Findings were robust to three sensitivity analyses taking different analytic approaches.

There were also a number of limitations. First, the items used to assess smoking status did not specify regular smoking, meaning the group of former smokers encompassed a wide range of smoking histories, from very occasional use to heavy smoking. As such, our results may underestimate the health risks associated with former (regular) smoking. Second, physical activity was self-reported, introducing scope for bias. A recent study documented notable discrepancy between objective measures and self-reports of physical activity, including an age-related decline in activity levels captured by accelerometery that was not observed in self-reports (52). In addition, levels of physical activity were dichotomised for analysis, distinguishing between those who engaged in moderate or vigorous activities more than once a week and those who engaged in less frequent moderate or vigorous activities. Replication of these analyses using a more objective and detailed measure of physical activity would be useful in validating our results. Third, chronic disease outcomes were based on self-reports of doctor diagnosis, and it is possible some may have been forgotten or not reported. However, validation studies comparing self-reports against medical records generally show high agreement (53). Fourth, while we included participants who reported the onset of health problems in any wave, regardless of whether they were retained in ELSA through to final follow-up at Wave 8, we excluded from our primary analyses those who did not report health problems or depressive symptoms prior to dropout. This group likely included individuals suffering from the conditions we were studying, but who died before the diseases were identified or could be reported in an ELSA interview. As such, our results may underestimate the impact of our exposures on the health outcomes of interest, although a sensitivity analysis based on imputed data produced similar estimates of associations. There were several differences between the analysed sample and participants we excluded, with the analysed sample generally more advantaged, healthier, and less likely to smoke or have low levels of physical activity. As such, our results may not generalise to the entire older population in England. Insofar that a synergistic effect of smoking and low physical activity is greater in less advantaged groups, then the current study could have underestimated the overall effect. In addition, we did not model dynamic effects (i.e. the impact of changes in smoking status and physical activity across the time period on disease outcomes) which may have masked some associations, although previous analyses of the ELSA cohort suggest that smoking status and level of physical activity remain stable across waves for the majority of participants (54). Fifth, although we had a large sample, the number of incident diagnoses was relatively small meaning we likely lacked statistical power to detect significant effects. Indeed, Bayes factors indicated that while the data supported

For peer review only - http://bmjopen.bmj.com/site/about/guidelines.xhtml

the null hypothesis (i.e. no synergistic effects of smoking and physical activity), there was some data insensitivity which meant we were unable to rule out small and medium-sized effects. The small number of incident cases also meant we were unable to conduct more fine-grained analyses, for example focusing on specific cancer types (e.g. lung, breast, colorectal) that might be affected by the exposures. Finally, while we adjusted for a range of potential confounders, there were no data available on substance misuse (aside from alcohol intake, which we controlled for) or diet quality. These variables have been associated to varying degrees with our exposures (15–17,55) and outcomes of interest (56–59). Further research is required to validate our findings with adjustment for these variables. There is also potential for residual confounding by socioeconomic position if there were aspects of this that were not accounted for by our adjustment for non-pension wealth.

Conclusions

The present results are not suggestive of large synergistic effects of smoking and low levels of physical activity on risk of developing chronic disease or clinically relevant depressive symptoms (although smaller synergistic effects cannot be ruled out). However, additive effects of smoking and low activity were evident, underscoring the importance of each of these behaviours for disease onset.

For peer review only - http://bmjopen.bmj.com/site/about/guidelines.xhtml

List of abbreviations

ANOVA – analysis of variance

BF – Bayes factor

BMI – body mass index

CHD – coronary heart disease

CI – confidence interval

ELSA – English Longitudinal Study of Ageing

RCT – randomised controlled trial

RR – relative risk

For peer review only

For peer review only - http://bmjopen.bmj.com/site/about/guidelines.xhtml

Declarations

Ethics approval and consent to participate

Ethical approval for the English Longitudinal Study of Ageing was provided by the London Multi-Centre Research Ethics Committee (MREC/01/2/91) and participants provided full informed consent.

Availability of data and material

The raw ELSA data are freely available to download from the UK Data Service.

Competing interests

J Brown has received unrestricted research funding from Pfizer, who manufacture smoking cessation medications. L Shahab has received a research grant and honoraria for a talk and travel expenses from manufacturers of smoking cessation medications (Pfizer and Johnson & Johnson). All authors declare no financial links with tobacco companies or e-cigarette manufacturers or their representatives. All other authors declare no competing interests.

Funding

This work was supported by Cancer Research UK (C1417/A22962) and the Economic and Social Research Council (ES/R005990/1). The funders had no final role in the study design; in the collection, analysis and interpretation of data; in the writing of the report; or in the decision to submit the paper for publication. All researchers listed as authors are independent from the funders and all final decisions about the research were taken by the investigators and were unrestricted. All authors had full access to all of the data (including statistical reports and tables) in the study and can take responsibility for the integrity of the data and the accuracy of the data analysis.

Authors' contributions

SE Jackson and L Smith conceived the study. J Brown, M Ussher, L Shahab and A Steptoe contributed to the study design. SE Jackson analysed and interpreted the data, and drafted the manuscript. All authors revised the manuscript critically for intellectual content, and read and approved the final manuscript.

For peer review only - http://bmjopen.bmj.com/site/about/guidelines.xhtml

References

1. Lopez AD, Mathers CD, Ezzati M, Jamison DT, Murray CJ, editors. Global Burden of Disease and Risk Factors [Internet]. Washington (DC): World Bank; 2006 [cited 2018 Oct 2]. Available from: http://www.ncbi.nlm.nih.gov/books/NBK11812/

2. Warburton DER, Nicol CW, Bredin SSD. Health benefits of physical activity: the evidence. Can Med Assoc J. 2006 Mar 14;174(6):801–9.

3. Penedo FJ, Dahn JR. Exercise and well-being: a review of mental and physical health benefits associated with physical activity. Curr Opin Psychiatry. 2005 Mar;18(2):189.

4. U.S. Department of Health and Human Services. The Health Consequences of Smoking—50 Years of Progress: A Report of the Surgeon General. Atlanta, GA: U.S. Department of Health and Human Services, Centers for Disease Control and Prevention, National Center for Chronic Disease Prevention and Health Promotion, Office on Smoking and Health; 2014.

5. Mokdad AH, Marks JS, Stroup DF, Gerberding JL. Actual causes of death in the United States, 2000. Jama. 2004;291(10):1238–1245.

6. Anthonisen NR, Skeans MA, Wise RA, Manfreda J, Kanner RE, Connett JE. The Effects of a Smoking Cessation Intervention on 14.5-Year Mortality: A Randomized Clinical Trial. Ann Intern Med. 2005 Feb 15;142(4):233–9.

7. Hamer M, Lavoie KL, Bacon SL. Taking up physical activity in later life and healthy ageing: the English longitudinal study of ageing. Br J Sports Med. 2014 Feb 1;48(3):239–43.

8. Jha P, Ramasundarahettige C, Landsman V, Rostron B, Thun M, Anderson RN, et al. 21st-Century Hazards of Smoking and Benefits of Cessation in the United States. N Engl J Med. 2013 Jan 24;368(4):341–50.

9. Hallgren M, Nguyen T-T-D, Herring MP, McDowell CP, Gordon BR, Stubbs B, et al. Associations of physical activity with anxiety symptoms and disorders: Findings from the Swedish National March Cohort. Gen Hosp Psychiatry. 2019 Mar 10;58:45–50.

10. Schuch FB, Vancampfort D, Firth J, Rosenbaum S, Ward PB, Silva ES, et al. Physical Activity and Incident Depression: A Meta-Analysis of Prospective Cohort Studies. Am J Psychiatry. 2018 Jul 1;175(7):631–48.

11. Kendler KS, Neale MC, MacLean CJ, Heath AC, Eaves LJ, Kessler RC. Smoking and Major Depression: A Causal Analysis. Arch Gen Psychiatry. 1993 Jan 1;50(1):36–43.

12. Fergusson DM, Goodwin RD, Horwood LJ. Major depression and cigarette smoking: results of a 21-year longitudinal study. Psychol Med. 2003 Nov;33(8):1357–67.

13. Boden JM, Fergusson DM, Horwood LJ. Cigarette smoking and depression: tests of causal linkages using a longitudinal birth cohort. Br J Psychiatry. 2010 Jun;196(6):440–6.

For peer review only - http://bmjopen.bmj.com/site/about/guidelines.xhtml

14. Wootton RE, Richmond RC, Stuijfzand BG, Lawn RB, Sallis HM, Taylor GMJ, et al. Causal effects of lifetime smoking on risk for depression and schizophrenia: Evidence from a Mendelian randomisation study. bioRxiv. 2018 Dec 20;381301.

15. Poortinga W. The prevalence and clustering of four major lifestyle risk factors in an English adult population. Prev Med. 2007 Feb 1;44(2):124–8.

16. Chiolero A, Wietlisbach V, Ruffieux C, Paccaud F, Cornuz J. Clustering of risk behaviors with cigarette consumption: A population-based survey. Prev Med. 2006 May 1;42(5):348–53.

17. Berrigan D, Dodd K, Troiano RP, Krebs-Smith SM, Barbash RB. Patterns of health behavior in U.S. adults. Prev Med. 2003 May 1;36(5):615–23.

18. Marrero JA, Fontana RJ, Fu S, Conjeevaram HS, Su GL, Lok AS. Alcohol, tobacco and obesity are synergistic risk factors for hepatocellular carcinoma. J Hepatol. 2005 Feb 1;42(2):218–24.

19. Freedman DM, Sigurdson AJ, Rajaraman P, Doody MM, Linet MS, Ron E. The Mortality Risk of Smoking and Obesity Combined. Am J Prev Med. 2006 Nov;31(5):355–62.

20. Li TY, Rana JS, Manson JE, Willett WC, Stampfer MJ, Colditz GA, et al. Obesity as Compared With Physical Activity in Predicting Risk of Coronary Heart Disease in Women. Circulation. 2006 Jan 31;113(4):499–506.

21. Slattery ML, Potter JD. Physical activity and colon cancer: confounding or interaction? Med Sci Sports Exerc. 2002 Jun;34(6):913–9.

22. Schlecht NF, Franco EL, Pintos J, Negassa A, Kowalski LP, Oliveira BV, et al. Interaction between Tobacco and Alcohol Consumption and the Risk of Cancers of the Upper Aero-Digestive Tract in Brazil. Am J Epidemiol. 1999 Dec 1;150(11):1129–37.

23. Prochaska JJ, Prochaska JO. A Review of Multiple Health Behavior Change Interventions for Primary Prevention. Am J Lifestyle Med. 2011 May 1;5(3):208–21.

24. Spring B, Moller AC, Coons MJ. Multiple health behaviours: overview and implications. J Public Health Oxf Engl. 2012 Mar;34 Suppl 1:i3-10.

25. Alageel S, Gulliford MC, McDermott L, Wright AJ. Multiple health behaviour change interventions for primary prevention of cardiovascular disease in primary care: systematic review and meta-analysis. BMJ Open. 2017 Jun 1;7(6):e015375.

26. Ussher MH, Taylor AH, Faulkner GEJ. Exercise interventions for smoking cessation. Cochrane Database Syst Rev [Internet]. 2014 [cited 2019 Mar 15];(8). Available from: https://www.cochranelibrary.com/cdsr/doi/10.1002/14651858.CD002295.pub5/full

27. Loprinzi PD, Walker JF, Kane C, Cardinal BJ. Physical activity moderates the association between nicotine dependence and depression among U.S. smokers. Am J Health Promot AJHP. 2014 Oct;29(1):37–42.

For peer review only

28. Vickers KS, Patten CA, Lane K, Clark MM, Croghan IT, Schroeder DR, et al. Depressed versus nondepressed young adult tobacco users: differences in coping style, weight concerns and exercise level. Health Psychol Off J Div Health Psychol Am Psychol Assoc. 2003 Sep;22(5):498–503.

29. Roberts V, Maddison R, Simpson C, Bullen C, Prapavessis H. The acute effects of exercise on cigarette cravings, withdrawal symptoms, affect, and smoking behaviour: systematic review update and meta-analysis. Psychopharmacology (Berl). 2012 Jul;222(1):1–15.

30. Haasova M, Warren FC, Ussher M, Janse Van Rensburg K, Faulkner G, Cropley M, et al. The acute effects of physical activity on cigarette cravings: systematic review and meta-analysis with individual participant data. Addict Abingdon Engl. 2013 Jan;108(1):26–37.

31. Bernard P, Ninot G, Cyprien F, Courtet P, Guillaume S, Georgescu V, et al. Exercise and Counseling for Smoking Cessation in Smokers With Depressive Symptoms: A Randomized Controlled Pilot Trial. J Dual Diagn. 2015;11(3–4):205–16.

32. Korhonen T, Goodwin A, Miesmaa P, Dupuis EA, Kinnunen T. Smoking cessation program with exercise improves cardiovascular disease biomarkers in sedentary women. J Womens Health 2002. 2011 Jul;20(7):1051–64.

33. Koubaa A, Triki M, Trabelsi H, Baati H, Sahnoun Z, Hakim A. The effect of a 12-week moderate intensity interval training program on the antioxidant defense capability and lipid profile in men smoking cigarettes or hookah: a cohort study. ScientificWorldJournal. 2015;2015:639369.

34. Garcia-Aymerich J, Lange P, Benet M, Schnohr P, Antó JM. Regular Physical Activity Modifies Smoking-related Lung Function Decline and Reduces Risk of Chronic Obstructive Pulmonary Disease. Am J Respir Crit Care Med. 2007 Mar 1;175(5):458–63.

35. Luo J, Iwasaki M, Inoue M, Sasazuki S, Otani T, Ye W, et al. Body mass index, physical activity and the risk of pancreatic cancer in relation to smoking status and history of diabetes: a large-scale population-based cohort study in Japan–The JPHC study. Cancer Causes Control. 2007 Aug 1;18(6):603–12.

36. Erens B, Primatesta P. Health survey for England 2000: cardiovascular disease. Lond Station Off. 2001;

37. Demakakos P, Hamer M, Stamatakis E, Steptoe A. Low-intensity physical activity is associated with reduced risk of incident type 2 diabetes in older adults: evidence from the English Longitudinal Study of Ageing. Diabetologia. 2010 May 22;53:1877–85.

38. Hamer M, Stamatakis E. Screen-Based Sedentary Behavior, Physical Activity, and Muscle Strength in the English Longitudinal Study of Ageing. PLOS ONE. 2013 Jun 3;8(6):e66222.

39. Hamer M, Stamatakis E. Prospective Study of Sedentary Behavior, Risk of Depression, and Cognitive Impairment. Med Sci Sports Exerc. 2014 Apr 1;46(4):718–23.

For peer review only - http://bmjopen.bmj.com/site/about/guidelines.xhtml

40. Hamer M, Molloy GJ, de Oliveira C, Demakakos P. Leisure time physical activity, risk of depressive symptoms, and inflammatory mediators: the English Longitudinal Study of Ageing. Psychoneuroendocrinology. 2009 Aug;34(7):1050–5.

41. Hamer M, de Oliveira C, Demakakos P. Non-exercise physical activity and survival: English longitudinal study of ageing. Am J Prev Med. 2014 Oct;47(4):452–60.

42. Steptoe A, Jackson SE. The Life Skills of Older Americans: Association with Economic, Psychological, Social, and Health Outcomes. Sci Rep. 2018 Jul 5;8(1):9669.

43. DeSalvo KB, Bloser N, Reynolds K, He J, Muntner P. Mortality prediction with a single general self-rated health question. A meta-analysis. J Gen Intern Med. 2006 Mar;21(3):267–75.

44. Jackson SE, Hackett RA, Steptoe A. Associations between age discrimination and health and wellbeing: cross-sectional and prospective analysis of the English Longitudinal Study of Ageing. Lancet Public Health. 2019;in press.

45. Radloff LS. The CES-D scale. Appl Psychol Meas. 1977;1(3):385–401.

46. Steffick DE. Documentation of affective functioning measures in the Health and Retirement Study. HRS Documentation Report DR-005; 2000.

47. Banks J, Karlsen S, Oldfield Z. Socio-economic position. 2003 [cited 2014 Mar 4]; Available from: http://discovery.ucl.ac.uk/15366/1/15366.pdf

48. Loprinzi PD, Walker JF. Nicotine Dependence, Physical Activity, and Sedentary Behavior among Adult Smokers. North Am J Med Sci. 2015 Mar;7(3):94–9.

49. Sweet SN, Fortier MS. Improving Physical Activity and Dietary Behaviours with Single or Multiple Health Behaviour Interventions? A Synthesis of Meta-Analyses and Reviews. Int J Environ Res Public Health. 2010 Apr;7(4):1720–43.

50. Perkins KA. Issues in the Prevention of Weight Gain after Smoking Cessation. Ann Behav Med. 1994 Jan 1;16(1):46–52.

51. Hyman DJ, Pavlik VN, Taylor WC, Goodrick GK, Moye L. Simultaneous vs sequential counseling for multiple behavior change. Arch Intern Med. 2007 Jun 11;167(11):1152–8.

52. Kapteyn A, Banks J, Hamer M, Smith JP, Steptoe A, Soest A van, et al. What they say and what they do: comparing physical activity across the USA, England and the Netherlands. J Epidemiol Community Health. 2018 Jun 1;72(6):471–6.

53. Bergmann MM, Byers T, Freedman DS, Mokdad A. Validity of Self-reported Diagnoses Leading to Hospitalization: A Comparison of Self-reports with Hospital Records in a Prospective Study of American Adults. Am J Epidemiol. 1998 May 15;147(10):969–77.

54. Banks J, Nazroo J, Steptoe A. The dynamics of ageing: Evidence from the English Longitudinal Study of ageing 2002-12 (Wave 6). 2014;

For peer review only - http://bmjopen.bmj.com/site/about/guidelines.xhtml

55. Kelly PJ, Baker AL, Deane FP, Kay-Lambkin FJ, Bonevski B, Tregarthen J. Prevalence of smoking and other health risk factors in people attending residential substance abuse treatment. Drug Alcohol Rev. 2012;31(5):638–44.

56. Quirk SE, Williams LJ, O'Neil A, Pasco JA, Jacka FN, Housden S, et al. The association between diet quality, dietary patterns and depression in adults: a systematic review. BMC Psychiatry. 2013 Jun 27;13(1):175.

57. Mente A, Koning L de, Shannon HS, Anand SS. A Systematic Review of the Evidence Supporting a Causal Link Between Dietary Factors and Coronary Heart Disease. Arch Intern Med. 2009 Apr 13;169(7):659–69.

58. Donaldson MS. Nutrition and cancer: A review of the evidence for an anti-cancer diet. Nutr J. 2004 Oct 20;3(1):19.

59. Stein MD. MEDICAL CONSEQUENCES OF SUBSTANCE ABUSE. Psychiatr Clin North Am. 1999 Jun 1;22(2):351–70.

For peer review only

Table 1. Sample characteristics at baseline overall and in relation to smoking status and level of physical activity

	Whole sample	Smoking status				Physical activity		
	(*n*=6425)	Never smoker (*n*=2387)	Former smoker (*n*=3141)	Current smoker (*n*=897)	*p*	High (*n*=4233)	Low (*n*=2192)	*p*
Age in years, mean (SD)	65.88 (9.34)	65.55 (9.17)	66.95 (9.60)	63.04 (8.12)	<0.001	64.77 (8.54)	68.03 (10.38)	<0.001
Sex, % (*n*)								
Men	45.2 (2902)	34.1 (813)	53.1 (1667)	47.0 (422)	<0.001	47.5 (2011)	40.6 (891)	<0.001
Women	54.8 (3523)	65.9 (1574)	46.9 (1474)	53.0 (475)	-	52.5 (2222)	59.4 (1301)	-
Ethnicity, % (*n*)								
White	98.8 (6345)	98.1 (2342)	99.3 (3118)	98.7 (885)	0.001	98.7 (4180)	98.8 (2165)	0.944
Non-white	1.2 (80)	1.9 (45)	0.7 (23)	1.3 (12)	-	1.3 (53)	1.2 (27)	-
Wealth quintile, % (*n*)								
1 (poorest)	14.6 (940)	11.5 (275)	13.0 (408)	28.7 (257)	<0.001	10.7 (451)	22.3 (489)	<0.001
2	18.5 (1188)	17.1 (407)	17.8 (559)	24.7 (222)	-	15.6 (661)	24.0 (527)	-
3	20.8 (1338)	21.3 (508)	20.9 (656)	19.4 (174)	-	21.4 (905)	19.8 (433)	-
4	22.3 (1432)	22.9 (546)	23.7 (743)	15.9 (143)	-	24.4 (1034)	18.2 (398)	-
5 (richest)	23.8 (1527)	27.3 (651)	24.7 (775)	11.3 (101)	-	27.9 (1182)	15.7 (345)	-
Alcohol intake, % (*n*)								
Never/rarely	18.9 (1213)	21.5 (513)	14.9 (468)	25.9 (232)	<0.001	15.4 (651)	25.6 (562)	<0.001
Regularly	45.3 (2909)	48.0 (1145)	44.3 (1393)	41.4 (371)	-	45.0 (1905)	45.8 (1004)	-
Frequently	35.8 (2303)	30.5 (729)	40.8 (1280)	32.8 (294)	-	39.6 (1677)	28.6 (626)	-
BMI, mean (SD)	27.91 (4.87)	27.84 (4.84)	28.19 (4.85)	27.14 (4.93)	<0.001	27.48 (4.44)	28.75 (5.51)	<0.001
Fair/poor self-rated health[1], % (*n*)	24.5 (1575)	19.7 (469)	24.5 (770)	37.5 (336)	<0.001	16.8 (709)	39.5 (866)	<0.001
Limiting long-standing illness[1], % (*n*)	32.9 (2111)	28.0 (668)	34.5 (1082)	40.2 (361)	<0.001	23.7 (1004)	50.5 (1107)	<0.001
Coronary heart disease[1], % (*n*)	8.6 (553)	6.7 (159)	10.3 (324)	7.8 (70)	<0.001	6.8 (286)	12.2 (267)	<0.001
Stroke[1], % (*n*)	2.4 (152)	1.6 (39)	2.9 (91)	2.5 (22)	0.009	1.5 (64)	4.0 (88)	<0.001
Cancer[1], % (*n*)	7.7 (496)	7.2 (171)	8.4 (263)	6.9 (62)	0.154	7.2 (303)	8.8 (193)	0.019
Chronic lung disease[1], % (*n*)	7.3 (466)	4.4 (105)	7.7 (241)	13.4 (120)	<0.001	5.4 (228)	10.9 (238)	<0.001
Clinically relevant depressive symptoms[1], % (*n*)	13.5 (860)	11.5 (273)	13.2 (411)	19.8 (176)	<0.001	9.7 (408)	20.8 (452)	<0.001
Smoking status, % (*n*)								
Never smoker	37.2 (2387)	100 (2387)	-	-	-	38.2 (1616)	35.2 (771)	<0.001
Former smoker	48.9 (3141)	-	100 (3141)	-	-	49.3 (2085)	48.2 (1056)	-
Current smoker	14.0 (897)	-	-	100 (897)	-	12.6 (532)	16.7 (365)	-
Level of physical activity, % (*n*)								
High	65.9 (4233)	67.7 (1616)	66.4 (2085)	59.3 (532)	<0.001	100 (4233)	-	-

For peer review only - http://bmjopen.bmj.com/site/about/guidelines.xhtml

Low	34.1 (2192)	32.3 (771)	33.6 (1056)	40.7 (365)	-	-	100 (2192)	-

BMI = body mass index; SD = standard deviation.

[1] Complete data on all health variables at baseline was not a prerequisite for inclusion, so there was a small amount of missing data across these variables. Valid percentages are presented for ease of interpretation.

For peer review only

Table 2. Main effects of smoking status and physical activity and the interaction between smoking status and physical activity for risks of incident health problems over 12-year follow-up

	Smoking status			Physical activity		Interaction[1]	
	Never smoker	Former smoker	Current smoker	High active	Low active	Former smoker x low active	Current smoker x low active
Fair/poor self-rated health							
% (*n*)	40.9 (529)	49.0 (744)	59.2 (225)	42.3 (999)	60.0 (499)	-	-
RR [95% CI]	1	1.20 [1.05-1.37]	1.45 [1.19-1.76]	1	1.42 [1.24-1.62]	-	-
p		0.008	<0.001		<0.001		
RR_{adj} [95% CI]	1	1.14 [0.97-1.35]	1.55 [1.22-1.99]	1	1.19 [0.95-1.49]	0.99 [0.74-1.34]	1.04 [0.68-1.59]
p		0.112	<0.001		0.141	0.954	0.846
Limiting long-standing illness							
% (*n*)	57.1 (720)	62.7 (905)	64.0 (240)	57.8 (1345)	69.2 (520)	-	-
RR [95% CI]	1	1.10 [0.97-1.24]	1.12 [0.93-1.35]	1	1.20 [1.05-1.37]	-	-
p		0.143	0.233		0.007		
RR_{adj} [95% CI]	1	1.07 [0.93-1.24]	1.16 [0.92-1.45]	1	1.06 [0.86-1.31]	0.97 [0.73-1.29]	1.08 [0.71-1.64]
p		0.359	0.205		0.564	0.845	0.719
Coronary heart disease							
% (*n*)	8.8 (117)	11.7 (176)	14.9 (59)	9.2 (214)	15.2 (138)	-	-
RR [95% CI]	1	1.33 [1.04-1.70]	1.69 [1.21-2.36]	1	1.66 [1.32-2.08]	-	-
p		0.023	0.002		<0.001		
RR_{adj} [95% CI]	1	1.13 [0.82-1.55]	1.93 [1.23-3.03]	1	1.19 [0.78-1.82]	1.19 [0.70-2.03]	1.15 [0.57-2.33]
p		0.454	0.004		0.412	0.521	0.704
Stroke							
% (*n*)	8.2 (113)	9.9 (159)	12.9 (54)	8.1 (196)	13.3 (130)	-	-
RR [95% CI]	1	1.22 [0.95-1.56]	1.58 [1.12-2.22]	1	1.64 [1.30-2.07]	-	-
p		0.129	0.009		<0.001		
RR_{adj} [95% CI]	1	1.13 [0.81-1.57]	1.74 [1.06-2.85]	1	1.40 [0.92-2.12]	0.81 [0.47-1.40]	1.28 [0.63-2.64]
p		0.491	0.028		0.115	0.449	0.496

CI = confidence interval; RR = relative risk from bivariate models; RR_{adj} = relative risk adjusted for age, sex, ethnicity, wealth, alcohol intake, body mass index, and physical activity (for smoking status) or smoking status (for physical activity).

[1] Multiplicative interaction between smoking status and physical activity. Results can be interpreted as the difference in RR between high active and low active former/current smokers, relative to the difference in RR between high and low active never smokers. Thus, an interaction term above 1 indicates the disparity between high and low active groups was greater for former/current smokers than never smokers, and an interaction term below 1 indicates the disparity between high and low active groups was smaller for former/current smokers than never smokers.

For peer review only - http://bmjopen.bmj.com/site/about/guidelines.xhtml

Note: For each outcome, the sample is restricted to those who did not report the presence of the outcome at baseline. Results therefore indicate the prevalence and relative risk of new-onset health problems over the follow-up period.

Table 2. (continued)

	Smoking status			Physical activity		Interaction[1]	
	Never smoker	Former smoker	Current smoker	High active	Low active	Former smoker x low active	Current smoker x low active
Cancer							
% (*n*)	13.4 (178)	15.8 (247)	17.1 (70)	13.6 (320)	18.5 (175)	-	-
RR [95% CI]	1	1.18 [0.96-1.45]	1.28 [0.95-1.72]	1	1.36 [1.11-1.66]	-	-
p		0.112	0.107		0.002		
RR_{adj} [95% CI]	1	1.11 [0.86-1.43]	1.44 [0.98-2.13]	1	1.30 [0.92-1.83]	1.01 [0.65-1.57]	1.01 [0.54-1.88]
p		0.445	0.067		0.139	0.970	0.977
Chronic lung disease							
% (*n*)	3.3 (44)	7.8 (120)	20.9 (82)	5.1 (119)	13.7 (127)	-	-
RR [95% CI]	1	2.34 [1.65-3.34]	6.28 [4.28-9.21]	1	2.67 [2.06-3.47]	-	-
p		<0.001	<0.001		<0.001		
RR_{adj} [95% CI]	1	2.77 [1.62-4.74]	8.33 [4.62-15.00]	1	3.50 [1.88-6.52]	0.56 [0.27-1.16]	0.48 [0.22-1.06]
p		<0.001	<0.001		<0.001	0.116	0.070
Clinically relevant depressive symptoms							
% (*n*)	53.5 (418)	56.4 (535)	62.4 (181)	52.8 (714)	62.9 (420)	-	-
RR [95% CI]	1	1.06 [0.90-1.24]	1.17 [0.94-1.46]	1	1.19 [1.02-1.39]	-	-
p		0.512	0.168		0.024		
RR_{adj} [95% CI]	1	1.09 [0.90-1.34]	1.16 [0.87-1.55]	1	1.09 [0.84-1.41]	0.95 [0.68-1.34]	1.07 [0.68-1.69]
p		0.381	0.309		0.511	0.782	0.764

CI = confidence interval; RR = relative risk from bivariate models; RR_{adj} = relative risk adjusted for age, sex, ethnicity, wealth, alcohol intake, body mass index, and physical activity (for smoking status) or smoking status (for physical activity).

[1] Multiplicative interaction between smoking status and physical activity. Results can be interpreted as the difference in RR between high active and low active former/current smokers, relative to the difference in RR between high and low active never smokers. Thus, an interaction term above 1 indicates the disparity between high and low active groups was greater for former/current smokers than never smokers, and an interaction term below 1 indicates the disparity between high and low active groups was smaller for former/current smokers than never smokers.

Note: For each outcome, the sample is restricted to those who did not report the presence of the outcome at baseline. Results therefore indicate the prevalence and relative risk of new-onset health problems over the follow-up period.

For peer review only

Table 3. Prevalence and unadjusted and adjusted relative risks of incident health problems over 12-year follow-up associated with each smoking/physical activity group

	Never smoker		Former smoker		Current smoker	
	High active	Low active	High active	Low active	High active	Low active
Fair/poor self-rated health						
% (*n*)	37.0 (354)	51.9 (48.1)	44.4 (504)	62.8 (240)	52.8 (141)	74.3 (84)
RR [95% CI]	1	1.40 [1.13-1.75]	1.20 [1.02-1.41]	1.70 [1.39-2.08]	1.43 [1.13-1.81]	2.01 [1.48-2.73]
p		0.002	0.027	<0.001	0.003	<0.001
RR_{adj} [95% CI]	1	1.19 [0.95-1.49]	1.14 [0.97-1.35]	1.35 [1.09-1.66]	1.55 [1.22-1.99]	1.92 [1.40-2.65]
p		0.141	0.112	0.006	<0.001	<0.001
Limiting long-standing illness						
% (*n*)	54.5 (516)	65.2 (204)	60.0 (662)	71.3 (243)	60.1 (167)	75.3 (73)
RR [95% CI]	1	1.20 [0.97-1.47]	1.10 [0.95-1.27]	1.31 [1.08-1.59]	1.10 [0.89-1.37]	1.38 [1.00-1.91]
p		0.089	0.189	0.007	0.384	0.049
RR_{adj} [95% CI]	1	1.06 [0.86-1.31]	1.07 [0.93-1.24]	1.11 [0.91-1.36]	1.16 [0.92-1.45]	1.33 [0.96-1.85]
p		0.564	0.359	0.321	0.205	0.091
Coronary heart disease						
% (*n*)	8.0 (78)	11.0 (39)	9.5 (104)	17.3 (72)	12.3 (32)	19.9 (27)
RR [95% CI]	1	1.38 [0.92-2.06]	1.19 [0.88-1.62]	2.17 [1.55-3.05]	1.53 [0.99-2.37]	2.48 [1.55-3.99]
p		0.119	0.259	<0.001	0.053	<0.001
RR_{adj} [95% CI]	1	1.19 [0.78-1.82]	1.13 [0.82-1.55]	1.60 [1.12-2.30]	1.93 [1.23-3.03]	2.64 [1.59-4.37]
p		0.412	0.454	0.011	0.004	<0.001
Stroke						
% (*n*)	6.7 (67)	12.0 (46)	8.9 (103)	12.6 (56)	9.7 (26)	18.5 (28)
RR [95% CI]	1	1.78 [1.20-2.64]	1.33 [0.96-1.82]	1.88 [1.30-2.73]	1.44 [0.90-2.32]	2.76 [1.72-4.43]
p		0.004	0.083	0.001	0.128	<0.001
RR_{adj} [95% CI]	1	1.40 [0.92-2.12]	1.13 [0.81-1.57]	1.27 [0.86-1.89]	1.74 [1.06-2.85]	3.12 [1.88-5.18
p		0.115	0.491	0.232	0.028	<0.001

CI = confidence interval; RR = relative risk; RR_{adj} = relative risk adjusted for age, sex, ethnicity, wealth, alcohol intake, and body mass index.
Note: For each outcome, the sample is restricted to those who did not report the presence of the outcome at baseline. Results therefore indicate the prevalence and relative risk of new-onset health problems over the follow-up period.

For Peer review only

Table 3. (continued)

	Never smoker		Former smoker		Current smoker	
	High active	Low active	High active	Low active	High active	Low active
Cancer						
% (*n*)	12.4 (120)	16.0 (58)	14.3 (160)	19.8 (87)	15.2 (40)	20.7 (30)
RR [95% CI]	1	1.29 [0.92-1.81]	1.15 [0.89-1.48]	1.60 [1.19-2.15]	1.22 [0.83-1.79]	1.67 [1.08-2.58]
p		0.136	0.276	0.002	0.307	0.022
RR_{adj} [95% CI]	1	1.30 [0.92-1.83]	1.11 [0.86-1.43]	1.45 [1.06-1.97]	1.44 [0.98-2.13]	1.89 [1.20-2.98]
p		0.139	0.445	0.019	0.067	0.006
Chronic lung disease						
% (*n*)	1.9 (18)	7.4 (26)	5.7 (63)	13.2 (57)	15.1 (38)	31.2 (44)
RR [95% CI]	1	3.96 [2.14-7.31]	3.07 [1.80-5.22]	7.07 [4.11-12.16]	8.13 [4.56-14.49]	16.76 [9.42-29.83]
p		<0.001	<0.001	<0.001	<0.001	<0.001
RR_{adj} [95% CI]	1	3.50 [1.88-6.52]	2.77 [1.62-4.74]	5.42 [3.11-9.44]	8.33 [4.62-15.00]	14.00 [7.68-25.53]
p		<0.001	<0.001	<0.001	<0.001	<0.001
Clinically relevant depressive symptoms						
% (*n*)	50.0 (269)	61.1 (149)	53.8 (343)	61.7 (192)	57.6 (102)	69.9 (79)
RR [95% CI]	1	1.22 [0.95-1.57]	1.08 [0.88-1.31]	1.24 [0.98-1.56]	1.15 [0.87-1.53]	1.40 [1.01-1.93]
p		0.118	0.470	0.075	0.328	0.042
RR_{adj} [95% CI]	1	1.09 [0.84-1.41]	1.09 [0.90-1.34]	1.14 [0.89-1.45]	1.16 [0.87-1.55]	1.36 [0.97-1.89]
p		0.511	0.381	0.297	0.309	0.071

CI = confidence interval; RR = relative risk; RR_{adj} = relative risk adjusted for age, sex, ethnicity, wealth, alcohol intake, and body mass index.
Note: For each outcome, the sample is restricted to those who did not report the presence of the outcome at baseline. Results therefore indicate the prevalence and relative risk of new-onset health problems over the follow-up period.

For peer review only

For peer review only - http://bmjopen.bmj.com/site/about/guidelines.xhtml

Figure legends

Figure 1. Relative risks of developing (a) fair/poor self-rated health, (b) limiting long-standing illness, (c) coronary heart disease, (d) stroke, (e) cancer, (f) chronic lung disease, and (g) clinically relevant depressive symptoms over 12-year follow-up by baseline smoking/physical activity status, among older adults free of these conditions at baseline.

For peer review only

For peer review only - http://bmjopen.bmj.com/site/about/guidelines.xhtml

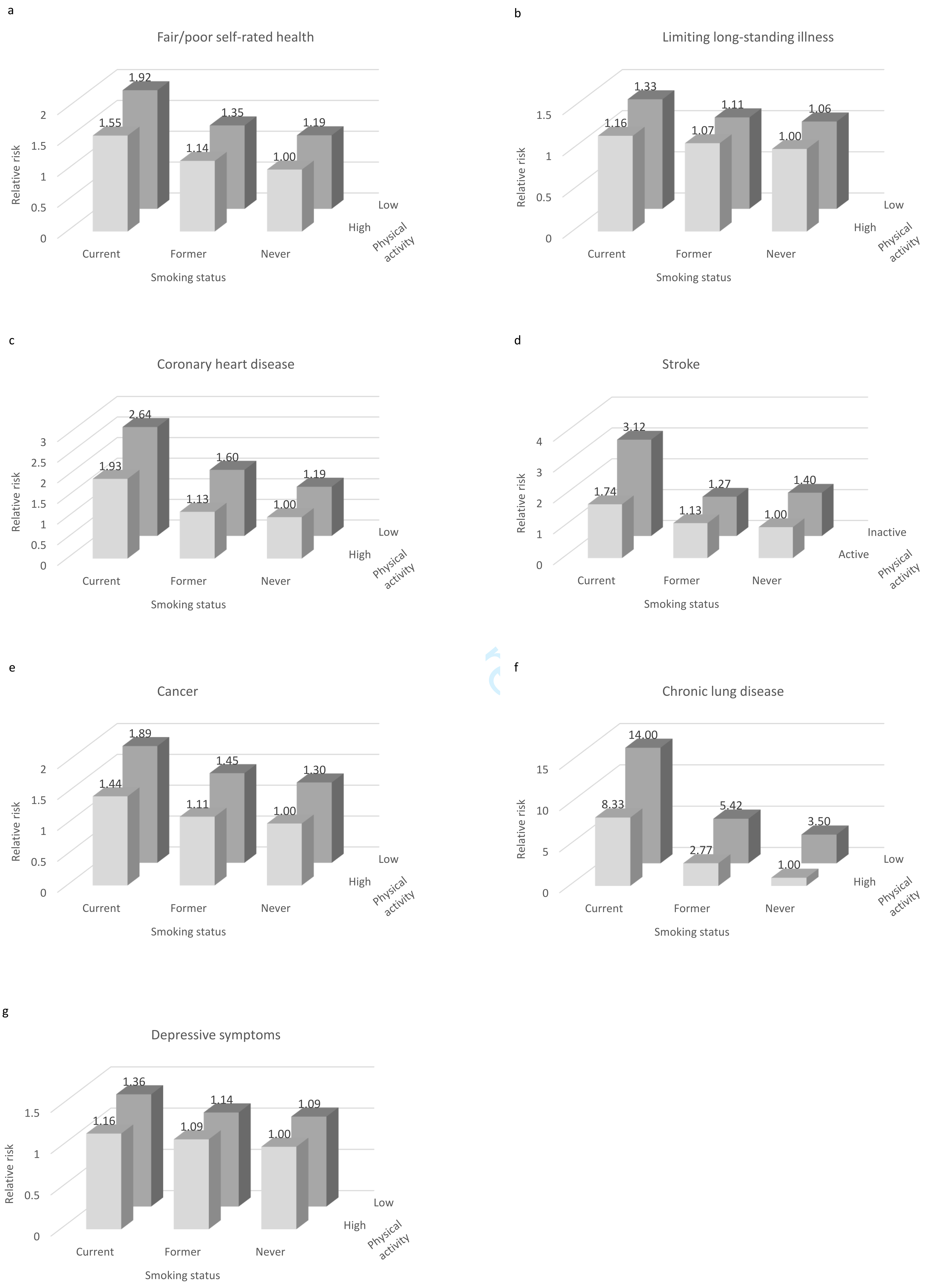
a
Fair/poor self-rated health
Relative risk
0
0.5
1
1.5
2
1.55
1.92
1.14
1.35
1.00
1.19
Current
Former
Never
Smoking status
Low
High
Physical activity
b
Limiting long-standing illness
Relative risk
0
0.5
1
1.5
1.16
1.33
1.07
1.11
1.00
1.06
Current
Former
Never
Smoking status
Low
High
Physical activity
c
Coronary heart disease
Relative risk
0
0.5
1
1.5
2
2.5
3
1.93
2.64
1.13
1.60
1.00
1.19
Current
Former
Never
Smoking status
Low
High
Physical activity
d
Stroke
Relative risk
0
1
2
3
4
1.74
3.12
1.13
1.27
1.00
1.40
Current
Former
Never
Smoking status
Inactive
Active
Physical activity
e
Cancer
Relative risk
0
0.5
1
1.5
2
1.44
1.89
1.11
1.45
1.00
1.30
Current
Former
Never
Smoking status
Low
High
Physical activity
f
Chronic lung disease
Relative risk
0
5
10
15
8.33
14.00
2.77
5.42
1.00
3.50
Current
Former
Never
Smoking status
Low
High
Physical activity
g
Depressive symptoms
Relative risk
0
0.5
1
1.5
1.16
1.36
1.09
1.14
1.00
1.09
Current
Former
Never
Smoking status
Low
High
Physical activity

Supplementary material

Method: calculation of Bayes factors

We used a conservative approach with alternative hypotheses represented by a half-normal distribution and expected effect sizes to RR=3 based on previous research that demonstrated a large multiplicative effect of smoking and BMI on circulatory disease mortality (1), and RR=2 and RR=1.5 to test for medium and small synergistic effects. BFs ≥3 can be interpreted as evidence for the alternative hypothesis (and against the null), BFs ≤1/3 as evidence for the null hypothesis, and BFs between 1/3 and 3 suggest the data are insensitive to distinguish the alternative hypothesis from the null (2).

Results: sensitivity analyses

Imputing outcomes at Wave 8 for participants who dropped out and did not report the presence of the outcome prior to dropout produced a very similar pattern of results, but the larger sample size meant 95% CIs were narrower and *p* values were smaller (Supplementary Table 2, Supplementary Figure 1). Multiplicative interactions between smoking and physical activity remained non-significant for all outcomes, with the exception of chronic lung disease which became statistically significant (p=0.044).

Restricting the sample to those with complete data at baseline (Wave 2) and final follow-up (Wave 8) also produced a similar pattern of results, although RRs were attenuated for the diagnosed diseases (CHD, stroke, cancer, chronic lung disease) (Supplementary Table 3, Supplementary Figure 2). The only notable change was that the RR for CHD in low active current smokers fell below that of low active former smokers, but this was likely caused by the reduced sample size leading to imprecise estimates for this group (*n*=121, 12 cases of incident CHD). Interactions between smoking and physical activity remained non-significant for all outcomes.

Excluding current smokers with lower levels of dependence (i.e. those who smoked <15 cigarettes per day, *n*=369) did not notably alter the results (Supplementary Table 4, Supplementary Figure 3), although as was observed when missing data were imputed, the interaction between smoking and physical activity for risk of developing chronic lung disease became statistically significant (p=0.033).

For peer review only - http://bmjopen.bmj.com/site/about/guidelines.xhtml

References

1. Freedman DM, Sigurdson AJ, Rajaraman P, Doody MM, Linet MS, Ron E. The Mortality Risk of Smoking and Obesity Combined. Am J Prev Med. 2006 Nov;31(5):355–62.

2. Dienes Z. Using Bayes to get the most out of non-significant results. Front Psychol [Internet]. 2014 Jul 29 [cited 2018 Jul 4];5. Available from: https://www.ncbi.nlm.nih.gov/pmc/articles/PMC4114196/

For peer review only

For peer review only - http://bmjopen.bmj.com/site/about/guidelines.xhtml

Supplementary tables

Supplementary Table 1. Baseline characteristics of participants included in the analysed sample compared with those who were excluded

	Excluded (*n*=3007)	Included (*n*=6425)	*p*
Age in years, mean (SD)	65.60 (13.28)	65.88 (9.34)	0.229
Sex, % (*n*)			
Men	40.7 (1223)	45.2 (2902)	<0.001
Women	59.3 (1784)	54.8 (3523)	-
Ethnicity, % (*n*)			
White	95.0 (2847)	98.8 (6345)	<0.001
Non-white	5.0 (149)	1.2 (80)	-
Wealth quintile, % (*n*)			
1 (poorest)	28.8 (643)	14.6 (940)	<0.001
2	24.0 (536)	18.5 (1188)	-
3	18.0 (403)	20.8 (1338)	-
4	15.3 (341)	22.3 (1432)	-
5 (richest)	14.0 (313)	23.8 (1527)	-
Alcohol intake, % (*n*)			
Never/rarely	23.6 (406)	18.9 (1213)	<0.001
Regularly	42.4 (728)	45.3 (2909)	-
Frequently	34.0 (585)	35.8 (2303)	-
BMI, mean (SD)	17.72 (14.48)	27.91 (4.87)	<0.001
Fair/poor self-rated health[1], % (*n*)	35.1 (1008)	24.5 (1575)	<0.001
Limiting long-standing illness[1], % (*n*)	40.9 (1230)	32.9 (2111)	<0.001
Coronary heart disease[1], % (*n*)	9.9 (299)	8.6 (553)	0.035
Stroke[1], % (*n*)	5.1 (153)	2.4 (152)	<0.001
Cancer[1], % (*n*)	6.9 (208)	7.7 (496)	0.167
Chronic lung disease[1], % (*n*)	7.4 (223)	7.3 (466)	0.777
Clinically relevant depressive symptoms[1], % (*n*)	20.3 (565)	13.5 (860)	<0.001
Smoking status, % (*n*)			
Never smoker	36.1 (1085)	37.2 (2387)	<0.001
Former smoker	44.6 (1341)	48.9 (3141)	-
Current smoker	19.2 (578)	14.0 (897)	-
Level of physical activity, % (*n*)			
High	53.5 (1536)	65.9 (4233)	<0.001
Low	46.5 (1336)	34.1 (2192)	-

BMI = body mass index; SD = standard deviation. Note: due to missing data, numbers do not sum to the total and percentages do not sum to 100 for the group of excluded participants for some variables.

For peer review only

Supplementary Table 2. Bayes factors for multiplicative interactions between smoking status and physical activity

	Large effects (RR=3)		Medium effects (RR=2)		Small effects (RR=1.5)	
	BF	Interpretation	BF	Interpretation	BF	Interpretation
Fair/poor self-rated health	0.23	Moderate evidence for H0	0.36	Data were insensitive	0.56	Data were insensitive
Limiting long-standing illness	0.28	Moderate evidence for H0	0.43	Data were insensitive	0.65	Data were insensitive
Coronary heart disease	0.48	Data were insensitive	0.68	Data were insensitive	0.88	Data were insensitive
Stroke	0.62	Data were insensitive	0.84	Data were insensitive	1.02	Data were insensitive
Cancer	0.30	Moderate evidence for H0	0.45	Data were insensitive	0.65	Data were insensitive
Chronic lung disease	0.04	Strong evidence for H0	0.06	Strong evidence for H0	0.11	Moderate evidence for H0
Clinically relevant depressive symptoms	0.29	Moderate evidence for H0	0.43	Data were insensitive	0.65	Data were insensitive

BF=Bayes factor, H0=null hypothesis, RR=relative risk.

For peer review only

Supplementary Table 3. Main effects of smoking status and physical activity and the interaction between smoking status and physical activity for risks of incident health problems over 12-year follow-up: imputed outcome data for participants who dropped out before Wave 8

	Smoking status			Physical activity		Interaction[1]	
	Never smoker	Former smoker	Current smoker	High active	Low active	Former smoker x low active	Current smoker x low active
Fair/poor self-rated health							
% (*n*)	48.9 (1168)	58.5 (1839)	69.7 (625)	48.9 (2071)	71.2 (1562)	-	-
RR [95% CI]	1	1.20 [1.09-1.32]	1.42 [1.25-1.63]	1	1.46 [1.33-1.60]	-	-
p		<0.001	<0.001		<0.001		
RR_{adj} [95% CI]	1	1.15 [1.02-1.30]	1.52 [1.27-1.81]	1	1.23 [1.05-1.44]	0.95 [0.78-1.15]	0.97 [0.74-1.27]
p		0.023	<0.001		0.012	0.567	0.810
Limiting long-standing illness							
% (*n*)	62.8 (1499)	69.8 (2192)	72.9 (654)	62.1 (2630)	78.2 (1715)	-	-
RR [95% CI]	1	1.11 [1.01-1.22]	1.16 [1.03-1.31]	1	1.26 [1.16-1.37]	-	-
p		0.025	0.019		<0.001		
RR_{adj} [95% CI]	1	1.08 [0.96-1.21]	1.18 [1.00-1.39]	1	1.11 [0.96-1.28]	0.98 [0.82-1.17]	1.06 [0.82-1.37]
p		0.195	0.053		0.166	0.786	0.666
Coronary heart disease							
% (*n*)	14.1 (335)	20.3 (639)	25.3 (227)	14.0 (594)	27.7 (607)	-	-
RR [95% CI]	1	1.45 [1.20-1.75]	1.79 [1.32-2.44]	1	1.97 [1.71-2.27]	-	-
p		<0.001	0.001		<0.001		
RR_{adj} [95% CI]	1	1.21 [0.96-1.53]	2.05 [1.43-2.94]	1	1.37 [1.02-1.82]	1.04 [0.71-1.54]	1.05 [0.69-1.61]
p		0.098	<0.001		0.034	0.829	0.810
Stroke							
% (*n*)	13.7 (327)	18.4 (579)	23.3 (209)	13.4 (567)	25.0 (547)	-	-
RR [95% CI]	1	1.34 [1.04-1.73]	1.70 [1.30-2.22]	1	1.86 [1.55-2.24]	-	-
p		0.027	0.001		<0.001		
RR_{adj} [95% CI]	1	1.20 [0.89-1.63]	2.03 [1.42-2.92]	1	1.42 [1.03-1.97]	0.87 [0.60-1.25]	1.01 [0.67-1.53]
p		0.216	<0.001		0.035	0.441	0.963

CI = confidence interval; RR = relative risk from bivariate models; RR_{adj} = relative risk adjusted for age, sex, ethnicity, wealth, alcohol intake, body mass index, and physical activity (for smoking status) or smoking status (for physical activity).

[1] Multiplicative interaction between smoking status and physical activity. Results can be interpreted as the difference in RR between high active and low active former/current smokers, relative to the difference in RR between high and low active never smokers. Thus, an interaction term above 1 indicates the disparity between high and low active groups was greater for former/current smokers than never smokers, and an interaction term below 1 indicates the disparity between high and low active groups was smaller for former/current smokers than never smokers.

Note: For each outcome, the sample is restricted to those who did not report the presence of the outcome at baseline. Results therefore indicate the prevalence and relative risk of new-onset health problems over the follow-up period.

For peer review only

Supplementary Table 3. (continued)

	Smoking status			Physical activity		Interaction[1]	
	Never smoker	Former smoker	Current smoker	High active	Low active	Former smoker x low active	Current smoker x low active
Cancer							
% (*n*)	18.0 (430)	21.9 (688)	24.6 (221)	17.7 (749)	26.9 (589)	-	-
RR [95% CI]	1	1.22 [1.06-1.40]	1.36 [1.07-1.73]	1	1.52 [1.21-1.90]	-	-
p		0.006	0.013		0.002		
RR_{adj} [95% CI]	1	1.13 [0.93-1.37]	1.57 [1.15-2.16]	1	1.34 [0.98-1.83]	0.96 [0.69-1.32]	0.95 [0.56-1.61]
p		0.207	0.006		0.065	0.792	0.831
Chronic lung disease							
% (*n*)	6.9 (164)	14.1 (443)	32.5 (292)	9.0 (382)	23.6 (517)	-	-
RR [95% CI]	1	2.05 [1.56-2.72]	4.76 [3.10-7.32]	1	2.61 [1.79-3.81]	-	-
p		<0.001	<0.001		0.001		
RR_{adj} [95% CI]	1	2.14 [1.42-3.24]	6.27 [3.88-10.09]	1	2.66 [1.65-4.27]	0.74 [0.49-1.12]	0.63 [0.40-0.99]
p		0.001	<0.001		<0.001	0.157	0.044
Clinically relevant depressive symptoms							
% (*n*)	52.8 (1259)	58.0 (1823)	62.9 (564)	52.4 (2219)	65.1 (1428)	-	-
RR [95% CI]	1	1.10 [0.98-1.24]	1.19 [1.02-1.39]	1	1.24 [1.13-1.36]	-	-
p		0.111	0.029		<0.001		
RR_{adj} [95% CI]	1	1.10 [0.96-1.26]	1.18 [0.98-1.43]	1	1.07 [0.91-1.27]	1.01 [0.84-1.22]	1.09 [0.82-1.43]
p		0.184	0.087		0.396	0.905	0.556

CI = confidence interval; RR = relative risk from bivariate models; RR_{adj} = relative risk adjusted for age, sex, ethnicity, wealth, alcohol intake, body mass index, and physical activity (for smoking status) or smoking status (for physical activity).

[1] Multiplicative interaction between smoking status and physical activity. Results can be interpreted as the difference in RR between high active and low active former/current smokers, relative to the difference in RR between high and low active never smokers. Thus, an interaction term above 1 indicates the disparity between high and low active groups was greater for former/current smokers than never smokers, and an interaction term below 1 indicates the disparity between high and low active groups was smaller for former/current smokers than never smokers.

Note: For each outcome, the sample is restricted to those who did not report the presence of the outcome at baseline. Results therefore indicate the prevalence and relative risk of new-onset health problems over the follow-up period.

For peer review only

Supplementary Table 4. Main effects of smoking status and physical activity and the interaction between smoking status and physical activity for risks of incident health problems over 12-year follow-up: sample restricted to participants with data at Wave 2 and Wave 8

	Smoking status			Physical activity		Interaction[1]	
	Never smoker	Former smoker	Current smoker	High active	Low active	Former smoker x low active	Current smoker x low active
Fair/poor self-rated health							
% (*n*)	31.5 (351)	39.0 (494)	45.4 (129)	32.9 (668)	47.9 (306)	-	-
RR [95% CI]	1	1.24 [1.06-1.45]	1.44 [1.14-1.84]	1	1.45 [1.24-1.71]	-	-
p		0.008	0.003		<0.001		
RR_{adj} [95% CI]	1	1.16 [0.96-1.41]	1.53 [1.13-2.05]	1	1.19 [0.90-1.56]	1.06 [0.74-1.52]	1.11 [0.65-1.91]
p		0.134	0.005		0.223	0.734	0.693
Limiting long-standing illness							
% (*n*)	47.5 (488)	52.6 (599)	50.0 (135)	48.2 (915)	57.1 (307)	-	-
RR [95% CI]	1	1.11 [0.96-1.28]	1.05 [0.83-1.33]	1	1.18 [1.01-1.39]	-	-
p		0.166	0.662		0.040		
RR_{adj} [95% CI]	1	1.09 [0.92-1.30]	1.08 [0.82-1.43]	1	1.07 [0.82-1.38]	0.98 [0.69-1.39]	1.18 [0.69-2.02]
p		0.321	0.572		0.626	0.926	0.543
Coronary heart disease							
% (*n*)	5.7 (73)	7.9 (114)	8.6 (32)	6.1 (138)	9.6 (81)	-	-
RR [95% CI]	1	1.39 [1.03-1.88]	1.52 [0.99-2.35]	1	1.56 [1.17-2.07]	-	-
p		0.033	0.056		0.002		
RR_{adj} [95% CI]	1	1.20 [0.82-1.76]	1.72 [0.99-2.98]	1	1.12 [0.66-1.88]	1.37 [0.72-2.63]	0.94 [0.38-2.35]
p		0.359	0.053		0.677	0.339	0.893
Stroke							
% (*n*)	5.4 (72)	6.5 (100)	8.1 (32)	5.6 (131)	7.9 (73)	-	-
RR [95% CI]	1	1.21 [0.89-1.65]	1.50 [0.98-2.31]	1	1.43 [1.06-1.92]	-	-
p		0.234	0.065		0.019		
RR_{adj} [95% CI]	1	1.02 [0.69-1.51]	1.42 [0.78-2.57]	1	1.07 [0.63-1.81]	1.15 [0.58-2.27]	1.59 [0.64-3.92]
p		0.918	0.247		0.812	0.682	0.315

CI = confidence interval; RR = relative risk from bivariate models; RR_{adj} = relative risk adjusted for age, sex, ethnicity, wealth, alcohol intake, body mass index, and physical activity (for smoking status) or smoking status (for physical activity).

[1] Multiplicative interaction between smoking status and physical activity. Results can be interpreted as the difference in RR between high active and low active former/current smokers, relative to the difference in RR between high and low active never smokers. Thus, an interaction term above 1 indicates the disparity between high and low active groups was greater for former/current smokers than never smokers, and an interaction term below 1 indicates the disparity between high and low active groups was smaller for former/current smokers than never smokers.

Note: For each outcome, the sample is restricted to those who did not report the presence of the outcome at baseline. Results therefore indicate the prevalence and relative risk of new-onset health problems over the follow-up period.

For peer review only - http://bmjopen.bmj.com/site/about/guidelines.xhtml

Supplementary Table 4. (continued)

	Smoking status			Physical activity		Interaction[1]	
	Never smoker	Former smoker	Current smoker	High active	Low active	Former smoker x low active	Current smoker x low active
Cancer							
% (*n*)	9.8 (125)	11.6 (173)	11.5 (44)	10.1 (228)	12.9 (114)	-	-
RR [95% CI]	1	1.19 [0.93-1.51]	1.17 [0.82-1.68]	1	1.28 [1.01-1.62]	-	-
p		0.162	0.388		0.044		
RR_{adj} [95% CI]	1	1.22 [0.91-1.64]	1.20 [0.74-1.94]	1	1.38 [0.93-2.06]	0.82 [0.49-1.39]	1.07 [0.51-2.25]
p		0.192	0.460		0.111	0.467	0.864
Chronic lung disease							
% (*n*)	2.6 (34)	5.0 (75)	14.4 (52)	3.4 (78)	9.4 (83)	-	-
RR [95% CI]	1	1.94 [1.28-2.93]	5.54 [3.54-8.66]	1	2.75 [2.00-3.78]	-	-
p		0.002	<0.001		<0.001		
RR_{adj} [95% CI]	1	2.67 [1.41-5.03]	7.02 [3.49-14.12]	1	4.09 [2.01-8.32]	0.47 [0.20-1.09]	0.45 [0.18-1.13]
p		0.002	<0.001		<0.001	0.077	0.088
Clinically relevant depressive symptoms							
% (*n*)	43.7 (283)	44.7 (334)	48.8 (104)	42.9 (480)	49.3 (241)	-	-
RR [95% CI]	1	1.02 [0.84-1.24]	1.12 [0.85-1.47]	1	1.15 [0.95-1.39]	-	-
p		0.832	0.430		0.147		
RR_{adj} [95% CI]	1	1.06 [0.84-1.33]	0.98 [0.68-1.41]	1	1.01 [0.74-1.38]	1.02 [0.67-1.55]	1.37 [0.77-2.41]
p		0.639	0.917		0.965	0.931	0.284

CI = confidence interval; RR = relative risk from bivariate models; RR_{adj} = relative risk adjusted for age, sex, ethnicity, wealth, alcohol intake, body mass index, and physical activity (for smoking status) or smoking status (for physical activity).

[1] Multiplicative interaction between smoking status and physical activity. Results can be interpreted as the difference in RR between high active and low active former/current smokers, relative to the difference in RR between high and low active never smokers. Thus, an interaction term above 1 indicates the disparity between high and low active groups was greater for former/current smokers than never smokers, and an interaction term below 1 indicates the disparity between high and low active groups was smaller for former/current smokers than never smokers.

Note: For each outcome, the sample is restricted to those who did not report the presence of the outcome at baseline. Results therefore indicate the prevalence and relative risk of new-onset health problems over the follow-up period.

For peer review only

Supplementary Table 5. Main effects of smoking status and physical activity and the interaction between smoking status and physical activity for risks of incident health problems over 12-year follow-up: excluding current smokers with low levels of nicotine dependence (<15 cigarettes/day)

	Smoking status			Physical activity		Interaction[1]	
	Never smoker	Former smoker	Current smoker	High active	Low active	Former smoker x low active	Current smoker x low active
**Fair/poor self-rated health**							
% (*n*)	40.9 (529)	49.0 (744)	63.8 (134)	42.2 (946)	59.3 (461)	-	-
RR [95% CI]	1	1.20 [1.05-1.37]	1.56 [1.23-1.98]	1	1.41 [1.22-1.61]	-	-
p		0.008	<0.001		<0.001		
RR_{adj} [95% CI]	1	1.14 [0.97-1.35]	1.75 [1.29-2.36]	1	1.19 [0.94-1.49]	0.99 [0.74-1.34]	0.99 [0.58-1.67]
p		0.115	<0.001		0.144	0.953	0.955
**Limiting long-standing illness**							
% (*n*)	57.1 (720)	62.7 (905)	70.8 (148)	58.2 (1282)	69.2 (491)	-	-
RR [95% CI]	1	1.10 [0.97-1.24]	1.24 [0.99-1.56]	1	1.19 [1.04-1.36]	-	-
p		0.143	0.067		0.012		
RR_{adj} [95% CI]	1	1.07 [0.93-1.24]	1.36 [1.03-1.79]	1	1.07 [0.86-1.32]	0.97 [0.73-1.29]	0.97 [0.58-1.63]
p		0.348	0.032		0.548	0.847	0.908
**Coronary heart disease**							
% (*n*)	8.8 (117)	11.7 (176)	14.9 (34)	9.0 (200)	14.9 (127)	-	-
RR [95% CI]	1	1.33 [1.04-1.70]	1.70 [1.13-2.55]	1	1.65 [1.30-2.09]	-	-
p		0.023	0.011		<0.001		
RR_{adj} [95% CI]	1	1.13 [0.82-1.55]	1.94 [1.10-3.41]	1	1.20 [0.79-1.83]	1.19 [0.70-2.02]	1.15 [0.49-2.69]
p		0.459	0.021		0.392	0.533	0.750
**Stroke**							
% (*n*)	8.2 (113)	9.9 (159)	12.6 (30)	7.9 (182)	13.1 (120)	-	-
RR [95% CI]	1	1.22 [0.95-1.56]	1.53 [1.00-2.35]	1	1.65 [1.29-2.10]	-	-
p		0.129	0.049		<0.001		
RR_{adj} [95% CI]	1	1.12 [0.80-1.57]	1.69 [0.87-3.26]	1	1.41 [0.93-2.13]	0.81 [0.47-1.39]	1.59 [0.65-3.89]
p		0.498	0.121		0.109	0.437	0.306

CI = confidence interval; RR = relative risk from bivariate models; RR_{adj} = relative risk adjusted for age, sex, ethnicity, wealth, alcohol intake, body mass index, and physical activity (for smoking status) or smoking status (for physical activity).

[1] Multiplicative interaction between smoking status and physical activity. Results can be interpreted as the difference in RR between high active and low active former/current smokers, relative to the difference in RR between high and low active never smokers. Thus, an interaction term above 1 indicates the disparity between high and low active groups was greater for former/current smokers than never smokers, and an interaction term below 1 indicates the disparity between high and low active groups was smaller for former/current smokers than never smokers.

Note: For each outcome, the sample is restricted to those who did not report the presence of the outcome at baseline. Results therefore indicate the prevalence and relative risk of new-onset health problems over the follow-up period.

For peer review only - http://bmjopen.bmj.com/site/about/guidelines.xhtml

Supplementary Table 5. (continued)

	Smoking status			Physical activity		Interaction[1]	
	Never smoker	Former smoker	Current smoker	High active	Low active	Former smoker x low active	Current smoker x low active
Cancer							
% (*n*)	13.4 (178)	15.8 (247)	19.8 (48)	13.8 (309)	18.4 (164)	-	-
RR [95% CI]	1	1.18 [0.96-1.45]	1.48 [1.05-2.10]	1	1.33 [1.08-1.63]	-	-
p		0.112	0.027		0.006		
RR_{adj} [95% CI]	1	1.11 [0.85-1.43]	1.85 [1.18-2.91]	1	1.29 [0.91-1.82]	1.01 [0.65-1.57]	0.81 [0.39-1.67]
p		0.446	0.008		0.149	0.974	0.565
Chronic lung disease							
% (*n*)	3.3 (44)	7.8 (120)	26.3 (61)	5.0 (111)	13.0 (114)	-	-
RR [95% CI]	1	2.34 [1.65-3.34]	7.89 [5.22-11.91]	1	2.60 [1.98-3.41]	-	-
p		<0.001	<0.001		<0.001		
RR_{adj} [95% CI]	1	2.80 [1.64-4.79]	11.40 [6.10-21.31]	1	3.49 [1.87-6.50]	0.57 [0.27-1.17]	0.40 [0.17-0.93]
p		<0.001	<0.001		<0.001	0.123	0.033
Clinically relevant depressive symptoms							
% (*n*)	53.5 (418)	56.4 (535)	63.0 (104)	52.4 (668)	62.5 (389)	-	-
RR [95% CI]	1	1.06 [0.90-1.24]	1.18 [0.90-1.55]	1	1.19 [1.02-1.40]	-	-
p		0.512	0.236		0.028		
RR_{adj} [95% CI]	1	1.10 [0.90-1.34]	1.18 [0.82-1.70]	1	1.09 [0.84-1.41]	0.95 [0.68-1.34]	1.11 [0.64-1.95]
p		0.370	0.375		0.527	0.783	0.705

CI = confidence interval; RR = relative risk from bivariate models; RR_{adj} = relative risk adjusted for age, sex, ethnicity, wealth, alcohol intake, body mass index, and physical activity (for smoking status) or smoking status (for physical activity).

[1] Multiplicative interaction between smoking status and physical activity. Results can be interpreted as the difference in RR between high active and low active former/current smokers, relative to the difference in RR between high and low active never smokers. Thus, an interaction term above 1 indicates the disparity between high and low active groups was greater for former/current smokers than never smokers, and an interaction term below 1 indicates the disparity between high and low active groups was smaller for former/current smokers than never smokers.

Note: For each outcome, the sample is restricted to those who did not report the presence of the outcome at baseline. Results therefore indicate the prevalence and relative risk of new-onset health problems over the follow-up period.

For peer review only

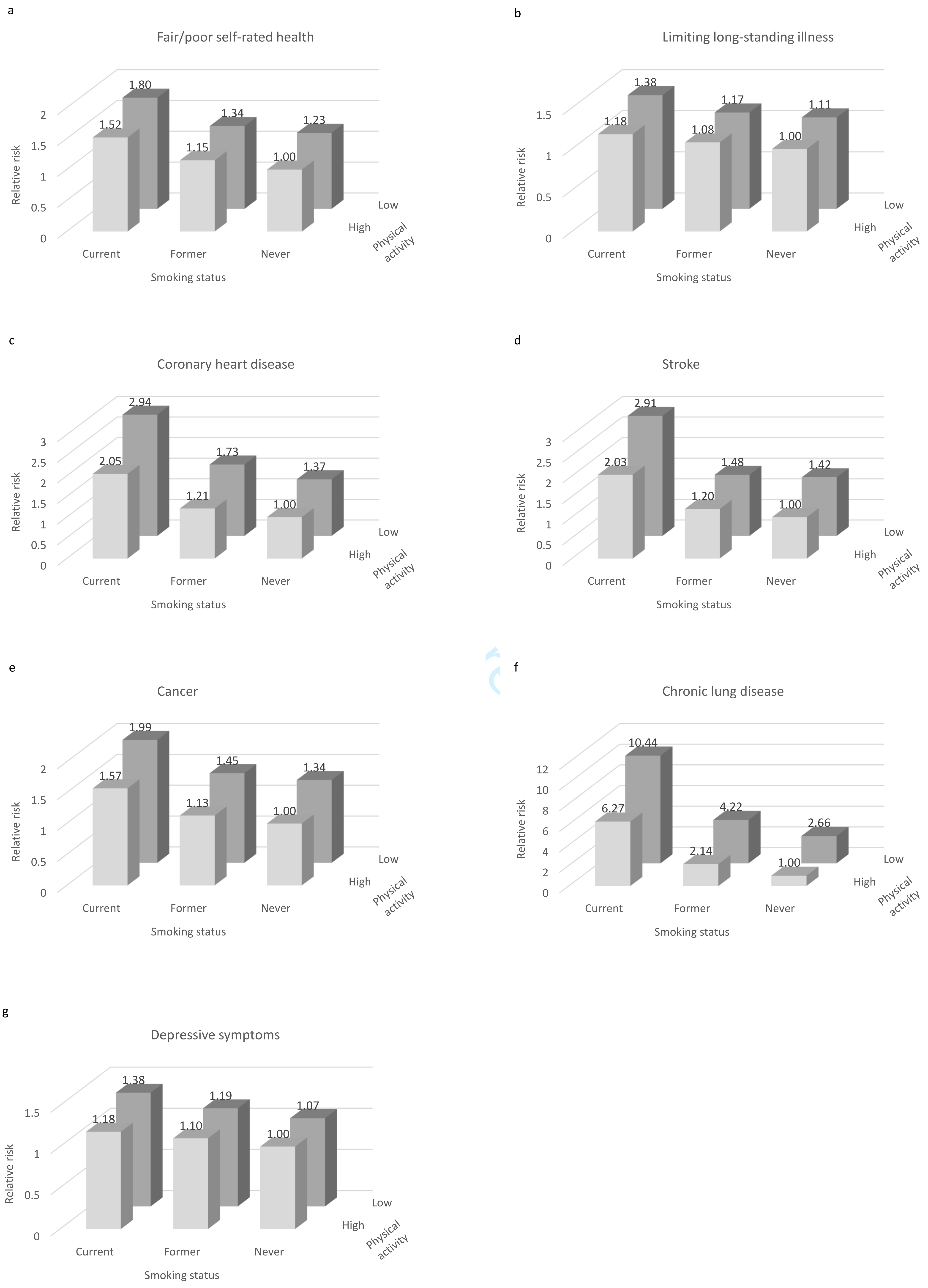
a
Fair/poor self-rated health
1.80
1.52
1.34
1.15
1.23
1.00
2
1.5
1
0.5
0
Relative risk
Current
Former
Never
Smoking status
Low
High
Physical activity
b
Limiting long-standing illness
1.38
1.18
1.17
1.08
1.11
1.00
1.5
1
0.5
0
Relative risk
Current
Former
Never
Smoking status
Low
High
Physical activity
c
Coronary heart disease
2.94
2.05
1.73
1.21
1.37
1.00
3
2.5
2
1.5
1
0.5
0
Relative risk
Current
Former
Never
Smoking status
Low
High
Physical activity
d
Stroke
2.91
2.03
1.48
1.20
1.42
1.00
3
2.5
2
1.5
1
0.5
0
Relative risk
Current
Former
Never
Smoking status
Low
High
Physical activity
e
Cancer
1.99
1.57
1.45
1.13
1.34
1.00
2
1.5
1
0.5
0
Relative risk
Current
Former
Never
Smoking status
Low
High
Physical activity
f
Chronic lung disease
10.44
6.27
4.22
2.14
2.66
1.00
12
10
8
6
4
2
0
Relative risk
Current
Former
Never
Smoking status
Low
High
Physical activity
g
Depressive symptoms
1.38
1.18
1.19
1.10
1.07
1.00
1.5
1
0.5
0
Relative risk
Current
Former
Never
Smoking status
Low
High
Physical activity

Supplementary Figure 1. Relative risks of developing (a) fair/poor self-rated health, (b) limiting long-standing illness, (c) coronary heart disease, (d) stroke, (e) cancer, (f) chronic lung disease, and (g) clinically relevant depressive symptoms over 12-year follow-up by baseline smoking/physical activity status, among older adults free of these conditions at baseline: imputed outcome data for participants who dropped out before Wave 8

For peer review only

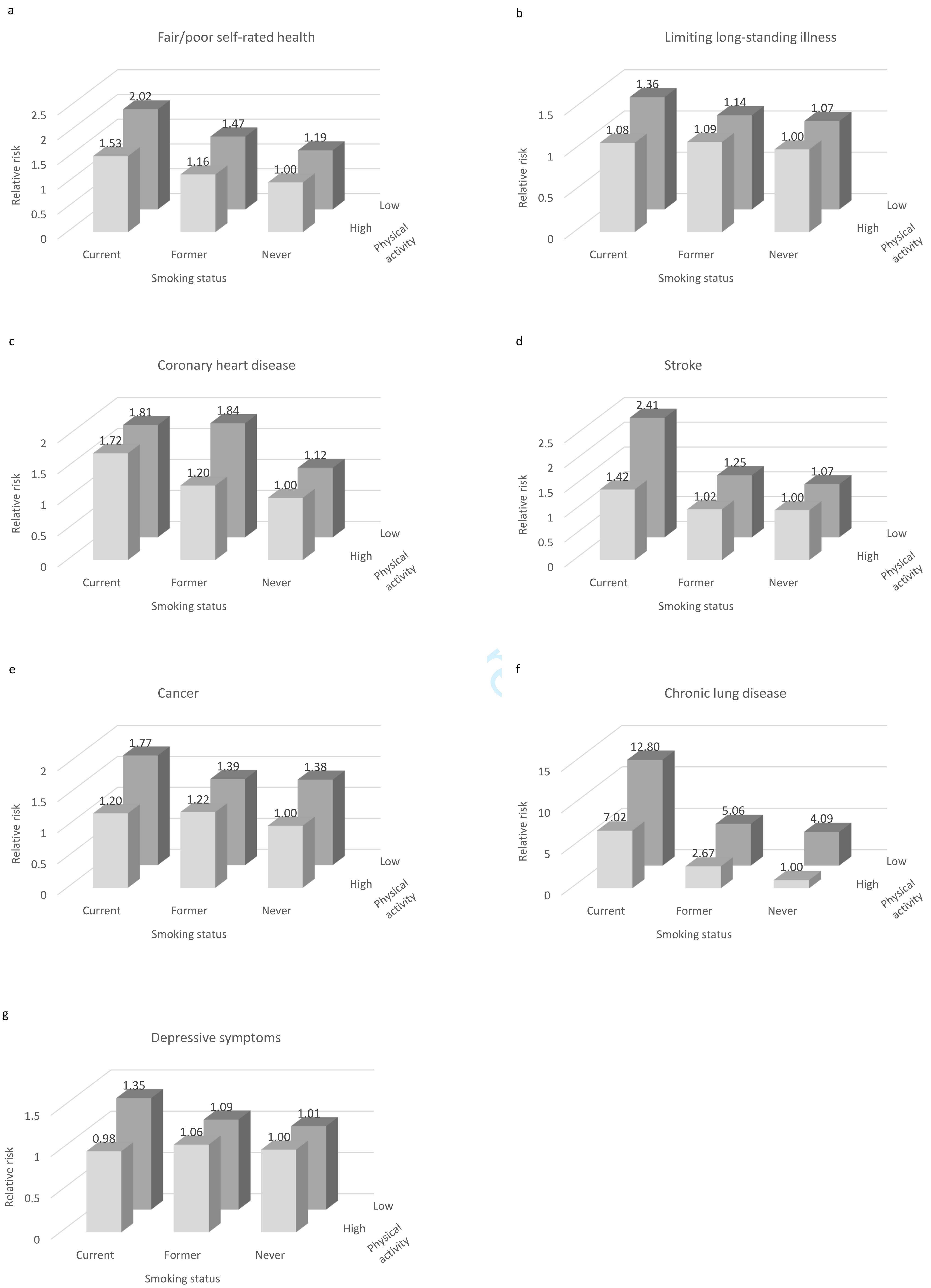
a
Fair/poor self-rated health
Relative risk
0
0.5
1
1.5
2
2.5
2.02
1.53
1.47
1.16
1.19
1.00
Low
High
Physical activity
Current
Former
Never
Smoking status
b
Limiting long-standing illness
Relative risk
0
0.5
1
1.5
1.36
1.08
1.14
1.09
1.07
1.00
Low
High
Physical activity
Current
Former
Never
Smoking status
c
Coronary heart disease
Relative risk
0
0.5
1
1.5
2
1.81
1.72
1.84
1.20
1.12
1.00
Low
High
Physical activity
Current
Former
Never
Smoking status
d
Stroke
Relative risk
0
0.5
1
1.5
2
2.5
2.41
1.42
1.25
1.02
1.07
1.00
Low
High
Physical activity
Current
Former
Never
Smoking status
e
Cancer
Relative risk
0
0.5
1
1.5
2
1.77
1.20
1.39
1.22
1.38
1.00
Low
High
Physical activity
Current
Former
Never
Smoking status
f
Chronic lung disease
Relative risk
0
5
10
15
12.80
7.02
5.06
2.67
4.09
1.00
Low
High
Physical activity
Current
Former
Never
Smoking status
g
Depressive symptoms
Relative risk
0
0.5
1
1.5
1.35
0.98
1.09
1.06
1.01
1.00
Low
High
Physical activity
Current
Former
Never
Smoking status

Supplementary Figure 2. Relative risks of developing (a) fair/poor self-rated health, (b) limiting long-standing illness, (c) coronary heart disease, (d) stroke, (e) cancer, (f) chronic lung disease, and (g) clinically relevant depressive symptoms over 12-year follow-up by baseline smoking/physical activity status, among older adults free of these conditions at baseline: sample restricted to participants with data at Wave 2 and Wave 8

For peer review only

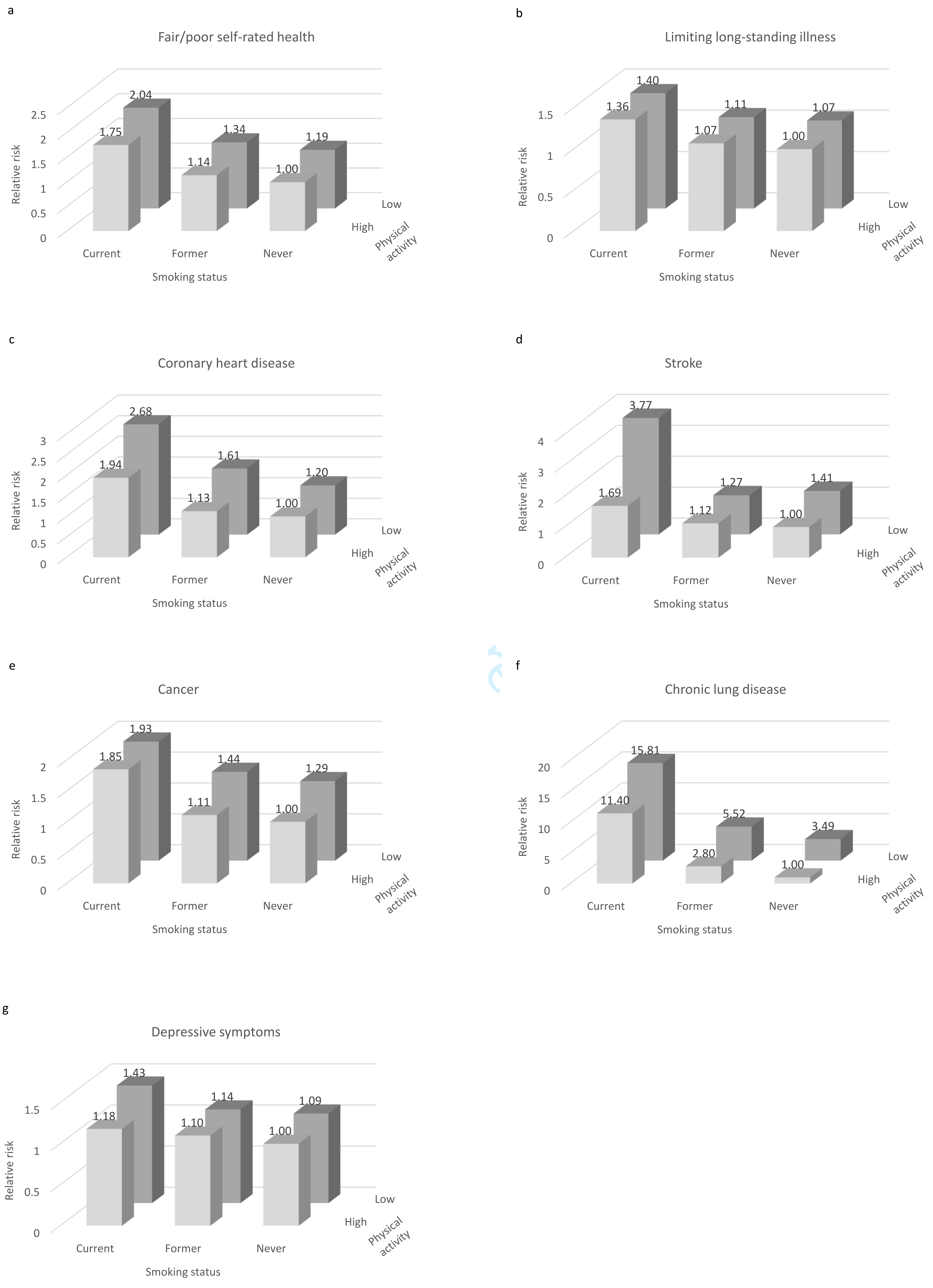
a
Fair/poor self-rated health
Relative risk
0
0.5
1
1.5
2
2.5
1.75
2.04
1.14
1.34
1.00
1.19
Current
Former
Never
Smoking status
Low
High
Physical activity
b
Limiting long-standing illness
Relative risk
0
0.5
1
1.5
1.36
1.40
1.07
1.11
1.00
1.07
Current
Former
Never
Smoking status
Low
High
Physical activity
c
Coronary heart disease
Relative risk
0
0.5
1
1.5
2
2.5
3
1.94
2.68
1.13
1.61
1.00
1.20
Current
Former
Never
Smoking status
Low
High
Physical activity
d
Stroke
Relative risk
0
1
2
3
4
1.69
3.77
1.12
1.27
1.00
1.41
Current
Former
Never
Smoking status
Low
High
Physical activity
e
Cancer
Relative risk
0
0.5
1
1.5
2
1.85
1.93
1.11
1.44
1.00
1.29
Current
Former
Never
Smoking status
Low
High
Physical activity
f
Chronic lung disease
Relative risk
0
5
10
15
20
11.40
15.81
2.80
5.52
1.00
3.49
Current
Former
Never
Smoking status
Low
High
Physical activity
g
Depressive symptoms
Relative risk
0
0.5
1
1.5
1.18
1.43
1.10
1.14
1.00
1.09
Current
Former
Never
Smoking status
Low
High
Physical activity

Supplementary Figure 3. Relative risks of developing (a) fair/poor self-rated health, (b) limiting long-standing illness, (c) coronary heart disease, (d) stroke, (e) cancer, (f) chronic lung disease, and (g) clinically relevant depressive symptoms over 12-year follow-up by baseline smoking/physical activity status, among older adults free of these conditions at baseline: excluding current smokers with low levels of nicotine dependence (<15 cigarettes/day)

For peer review only

STROBE Statement—Checklist of items that should be included in reports of ***cross-sectional studies***

	Item No	Recommendation	Page No
**Title and abstract**	1	(*a*) Indicate the study's design with a commonly used term in the title or the abstract	1
		(*b*) Provide in the abstract an informative and balanced summary of what was done and what was found	2
**Introduction**			
Background/rationale	2	Explain the scientific background and rationale for the investigation being reported	4-5
Objectives	3	State specific objectives, including any prespecified hypotheses	5
**Methods**			
Study design	4	Present key elements of study design early in the paper	6
Setting	5	Describe the setting, locations, and relevant dates, including periods of recruitment, exposure, follow-up, and data collection	6
Participants	6	(*a*) Give the eligibility criteria, and the sources and methods of selection of participants	6
Variables	7	Clearly define all outcomes, exposures, predictors, potential confounders, and effect modifiers. Give diagnostic criteria, if applicable	6-7
Data sources/ measurement	8*	For each variable of interest, give sources of data and details of methods of assessment (measurement). Describe comparability of assessment methods if there is more than one group	*6-7*
Bias	9	Describe any efforts to address potential sources of bias	7-8
Study size	10	Explain how the study size was arrived at	9
Quantitative variables	11	Explain how quantitative variables were handled in the analyses. If applicable, describe which groupings were chosen and why	6-8
Statistical methods	12	(*a*) Describe all statistical methods, including those used to control for confounding	7-8
		(*b*) Describe any methods used to examine subgroups and interactions	7-8
		(*c*) Explain how missing data were addressed	7-8
		(*d*) If applicable, describe analytical methods taking account of sampling strategy	Na
		(*e*) Describe any sensitivity analyses	8
**Results**			
Participants	13*	(a) Report numbers of individuals at each stage of study—eg numbers potentially eligible, examined for eligibility, confirmed eligible, included in the study, completing follow-up, and analysed	9
		(b) Give reasons for non-participation at each stage	Na
		(c) Consider use of a flow diagram	Na
Descriptive data	14*	(a) Give characteristics of study participants (eg demographic, clinical, social) and information on exposures and potential confounders	9
		(b) Indicate number of participants with missing data for each variable of interest	Tables
Outcome data	15*	Report numbers of outcome events or summary measures	Tables

For peer review only

Main results	16	(*a*) Give unadjusted estimates and, if applicable, confounder-adjusted estimates and their precision (eg, 95% confidence interval). Make clear which confounders were adjusted for and why they were included	Tables
		(*b*) Report category boundaries when continuous variables were categorized	Tables
		(*c*) If relevant, consider translating estimates of relative risk into absolute risk for a meaningful time period	Na
Other analyses	17	Report other analyses done—eg analyses of subgroups and interactions, and sensitivity analyses	10-11, tables
**Discussion**			
Key results	18	Summarise key results with reference to study objectives	11-12
Limitations	19	Discuss limitations of the study, taking into account sources of potential bias or imprecision. Discuss both direction and magnitude of any potential bias	13-14
Interpretation	20	Give a cautious overall interpretation of results considering objectives, limitations, multiplicity of analyses, results from similar studies, and other relevant evidence	12-13
Generalisability	21	Discuss the generalisability (external validity) of the study results	13-14
**Other information**			
Funding	22	Give the source of funding and the role of the funders for the present study and, if applicable, for the original study on which the present article is based	16

*Give information separately for exposed and unexposed groups.

Note: An Explanation and Elaboration article discusses each checklist item and gives methodological background and published examples of transparent reporting. The STROBE checklist is best used in conjunction with this article (freely available on the Web sites of PLoS Medicine at http://www.plosmedicine.org/, Annals of Internal Medicine at http://www.annals.org/, and Epidemiology at http://www.epidem.com/). Information on the STROBE Initiative is available at www.strobe-statement.org.

For peer review only

For peer review only - http://bmjopen.bmj.com/site/about/guidelines.xhtml
